# The good, the bad and the boa: An unexpected new species of a true boa revealed by morphological and molecular evidence

**DOI:** 10.1371/journal.pone.0298159

**Published:** 2024-04-17

**Authors:** Rodrigo Castellari Gonzalez, Lorena Corina Bezerra de Lima, Paulo Passos, Maria José J. Silva

**Affiliations:** 1 Museu de História Natural do Ceará Prof. Dias da Rocha, Universidade Estadual do Ceará, Pacoti, Ceará, Brazil; 2 Departamento de Vertebrados, Museu Nacional, Universidade Federal do Rio de Janeiro, Rio de Janeiro, Brazil; 3 Laboratório de Ecologia e Evolução, Instituto Butantan, São Paulo, Brazil; Laboratoire de Biologie du Développement de Villefranche-sur-Mer, FRANCE

## Abstract

Snakes of the genus *Boa* are outstanding elements of the New World biota with a broad sociological influence on pop culture. Historically, several taxa have been recognized in the past 300 years, being mostly described in the early days of binomial nomenclature. As a rule, these taxa were recognized based on a suite of phenotypic characters mainly those from the external morphology. However, there is a huge disagreement with respect to the current taxonomy and available molecular phylogenies. In order to reconcile both lines of evidence, we investigate the phylogenetic reconstruction (using mitochondrial and nuclear genes) of the genus in parallel to the detailed study of some phenotypic systems from a geographically representative sample of the cis-Andean mainland *Boa constrictor*. We used cyt-*b* only (744bp) from 73 samples, and cyt-*b*, ND4, NTF3, and ODC partial sequences (in a total of 2305 bp) from 35 samples, comprising nine currently recognized taxa (species or subspecies), to infer phylogenetic relationships of boas. Topologies recovered along all the analyses and genetic distances obtained allied to a unique combination of morphological traits (colouration, pholidosis, meristic, morphometric, and male genitalia features) allowed us to recognize *B*. *constrictor lato sensu*, *B*. *nebulosa*, *B*. *occidentalis*, *B*. *orophias* and a distinct lineage from the eastern coast of Brazil, which we describe here as a new species, diagnosing it from the previously recognized taxa. Finally, we discuss the minimally necessary changes in the taxonomy of *Boa constrictor* complex; the value of some usually disregarded phenotypic character system; and we highlight the urgency of continuing environmental policy to preserve one of the most impacted Brazilian hotspots, the Atlantic Forest, which represents an ecoregion full of endemism.

## Introduction

The constrictor snakes popularly referred to as boas are very well known both for science and for the general public since it is a common pet and also largely used in the leather and cinematographic industries [1–3]. Members of the genus *Boa* Linnaeus, 1758 are remarkably generalists with respect to several niche axes. The generalist habits of boas has led them to colonize the New World [3], with a distribution range from Mexico to Argentina [4–9], including several continental and oceanic islands [10–12]. Members of this genus are distributed over diverse vegetation types ranging from 30° of latitude north to 33° degrees latitude south, from sea level to a maximum altitude of 1,500 meters above sea level [1,6,10]. They have strong developed axial musculature used for constricting prey, and reach massive body sizes [13,14], although dwarfism has been reported on island populations [11,12,15,16]. Boas feed on birds [10,17,18], amphibians [19] mammals [20–23], and other reptiles [24].

The considerable variation in colour and patterns exhibited by these snakes has been traditionally used for the recognition of several subspecies [1]. However, the polychromatism phenomena added to the overlap in some meristic and/or morphometric data make the identification of these subspecies a hard task [3,25]. To date, no comprehensive taxonomic study grounded on hypothesis testing has been performed for the New World boas, mainly comprising the South American lineages.

Though there have been some modifications in the genus [26,27], the name *Boa constrictor* Linnaeus, 1758 has been stable for over 260 years [3,28,29]. Due to the loose boundaries among previously recognized taxa, scientists preferred to be more conservative and treat them as a single polytypic species (e.g. [5,29]), so several subspecies have been recognized as a single taxon, named *Boa constrictor* [25].

Hynkovà *et al*. [30] published the first study addressing the interrelationships for the *Boa constrictor* complex using a fragment of the cytochrome *b* mitochondrial gene (cyt *b*) from 115 individuals from many populations across the Neotropics. Such study, comprising six subspecies (*B*. *c*. *constrictor*, *B*. *c*. *imperator*, *B*. *c*. *longicauda*, *B*. *c*. *sabogae*, *B*. *c*. *amarali*, and *B*. *c*. *occidentalis*), included some "hybrid" representatives from different subspecies crossing (e.g., *B*. *c*. *constrictor* x *B*. *c*. *imperator*). Hynkovà *et al*. [30] recovered two main clades (into 67 haplotypes): one to Central America and another to South America. According to the results, there would also be genetic exchanges in border areas where individuals would have more contact, in northern Peru (subspecies *B*. *c*. *longicauda* and *B*. *c*. *ortonii*), Ecuador (subspecies *B*. *c*. *melanogaster*), Colombia and Venezuela. Hynkovà *et al*. [30] suggested that *B*. *c*. *imperator* might be elevated to full species rank, but they did not formalize this taxonomic proposal. They also recognized all populations from Central America clade under *B*. *c*. *imperator* (*sensu lato*), including *B*. *c*. *longicauda* (originally described from the Pacific coast of Peru) and *B*. *c*. *sabogae* (described from the Saboga Island in the Pacific coast of Panama). The authors recovered *B*. *c*. *occidentalis* as the most distinct haplotype within the South American Clade, being the sister group to other subspecies in this clade. Finally, still according to Hynkovà *et al*. [30], there was no molecular support to distinguish between *B*. *c*. *constrictor* and *B*. *c*. *amarali* in the South American clade.

Reynolds et al. [31] performed an extensive study using snakes of the families Boidae and Pythonidae, compiling a dataset that included 127 taxa and 11 loci (totalling up to 7561 bp per taxon). They recovered *B*. *c*. *occidentalis* as the sister group to *B*. *c*. *amarali* + *B*. *c*. *constrictor*, and *B*. *c*. *imperator* as the sister group to *B*. *i*. *sabogae*. These authors claimed to have found high support for the recognition of *B*. *imperator* in the specific rank as early proposed by Hynkovà *et al*. [30], although they admitted that a broader phylogenetic study throughout the distribution of *B*. *constrictor* (*sensu lato*) would be necessary to evaluate many of the previously proposed subspecies.

Suárez-Atilano *et al*. [32] used cyt *b* (1063 bp), Ornithine Decarboxylase (ODC—610 bp) nuclear intron and 10 microsatellite loci to infer phylogeographic structure, divergence times and historical demography of 149 individuals from *B*. *c*. *imperator* from Central America and Mexico. They recovered the dichotomy between samples from Central America/Mexico (North) and South America (South), and *B*. *c*. *occidentalis* as the sister group to other southern clade subspecies, corroborating the previous results by Hynkovà *et al*. [30]. In addition, Suárez-Atilano *et al*. [32] found two reciprocally monophyletic lineages in *B*. *c*. *imperator* associated with the main geographic barriers found in Mexico, which they named, respectively, PAC (Mexican Pacific Coast) and GYCA (Gulf of Mexico-Yucatan Peninsula-Central America), allied to five geographically differentiated genetic clusters. According to Suárez-Atilano *et al*. [32], these two *B*. *c*. *imperator* lineages have 4% genetic divergence among them and are in agreement with the genetic criteria established by Moritz [33] for recognizing evolutionary significant units (ESUs) and that, therefore, considers PAC and GYCA as ESUs. Suárez-Atilano *et al*. [32] further pointed that, although *B*. *c*. *imperator* is traditionally recognized throughout Mexico and Central America as a subspecies, the results obtained provided evidence that it can be considered as two distinct species.

Card *et al*. [11] used high-density RADseq data to identify relevant protein-coding genes that could be used for differentiating island and mainland populations of boas. According to their results, three dwarf island populations of boas would have independent origins. However, some sequences would have signs of convergence, implicating in strong connections amongst convergent phenotypes.

Currently, five species are recognized: *Boa constrictor* Linnaeus, 1758, *B*. *imperator* Daudin, 1803, *B*. *sigma* (Smith, 1943), *B*. *nebulosa* (Lazell, 1964), *B*. *orophias* Linnaeus, 1758, with some subspecies associate to *B*. *imperator* Daudin, 1803 along North and Central America (*B*. *i*. *sabogae* (Barbour, 1906)) and trans-Andean portion of South America (*B*. *i*. *eques* (Eydoux & Souleyet, 1841), *B*. *i*. *longicauda* (Price & Russo, 1991), *B*. *i*. *ortonii* (Cope, 1878)), and *B*. *constrictor* (*B*. *c*. *amarali* (Stull, 1932), *B*. *c*. *constrictor* Linnaeus, 1758 and *B*. *c*. *melanogaster* Langhammer, 1983) along mainland cis-Andean South America [3,34]. Nonetheless, despite several studies focusing on the genus diversity by using distinct molecular techniques and phylogeographic approaches [11,12,30,32,35], all of them pointed out a scenario claiming for a morphological review of the genus to understand its specific boundaries, taxa diagnosis and taxonomy [3–5,11,29,30,32,35–37].

### The Atlantic Forest, boas, and other “good” histories

When examining South-American specimens of boas, we came across a cohesive and geographically structured variation, phenotypic and molecular, found along the Brazilian Atlantic Forest populations.

The Atlantic Forest was originally a large ecosystem that covered about 1,5000.000 km^2^, mostly within Brazil. Considering its localization, the Portuguese colonizers harboured in Brazil in 1500 in the Atlantic coast, and since then the forest has been suffering deforestation for several purposes (e.g., logging, human occupation, industrialization, agricultural expansion) [38–42]. Nowadays, the Atlantic Forest contains the most populous areas in the country (70%), including the two largest Brazilian cities (São Paulo and Rio de Janeiro), so that the remaining 12% of this biome is highly fragmented [43,44]. This biome houses about 2500 vertebrates and 20,000 plant species, and hundreds of endemic species, and many others are yet to be discovered and described [45,46]. Due to its high levels of endemism and threats, the Atlantic Forest was considered a hotspot for conservation [41,47]. Nevertheless, very little has been done to prevent this habitat loss, since past Brazilian authorities simply ignore demands on conservation and also encourage further deforestation [43].

The epic spaghetti western movie ’The Good, the Bad and the Ugly’ is part of the classical Sergio Leone’s ‘Blood Money’ Trilogy, together with ‘A Fistful of Dollars’ and ‘For a Few Dollars More’. In the story, during the United States Civil War, three gunslingers know only a part of the treasure’s location and, so they decide to join forces to find the expected prize. Here, likewise the movie, we joined forces to find a better taxonomic resolution for the cis-Andean taxa from the *Boa constrictor* complex (= *Boa constrictor* Clade *sensu* Hynkovà *et al*., [30] based on the reconciliation between the ‘good’ molecular evidence and the aledgely ‘bad’ morphological evidence. We discuss the minimal changes in the taxonomy of *Boa constrictor* and allied taxa in order to accommodate the current knowledge on both monophyly and diagnosability. In addition, we highlight the urgency of continuing environmental policies to preserve what remains of the Brazilian hotspots which are largely impacted nowadays, such as Atlantic Forest, one of the hottest of the 36 global biodiversity hotspots [42].

## Material and methods

### Molecular analyses

#### Tissue samples, DNA extraction, and amplification

We have obtained tissue samples from 48 *Boa* individuals from museum collections, directly from fieldwork or from captive specimens (with known origin and without known hybridization, Table 1).

**Table 1:** Samples used in the present work for which unpublished sequences were produced, containing morphotype, voucher or collection number in the field, locality, state (available only for Brazil), country of origin, and geographical coordinates.

Sample	Taxa	Voucher	Locality	FU	Country	Latitude	Longitude	cyt b	ND4	NTF3	ODC	Map Number
1	*B*. *c*. *constrictor*	LCBL1	Rio Branco	AC	Brazil	-9.9560	-67.8670	x	x	x	x	1
2	*B*. *c*. *constrictor*	LCBL2	Senador Guiomard	AC	Brazil	-10.0696	-67.6091	x	x	x	x	2
3	*B*. *c*. *constrictor*	LCBL12	Manaus	AM	Brazil	-3.0038	-59.9187	x				3
4	*B*. *c*. *constrictor*	LCBL13	Manaus	AM	Brazil	-3.0038	-59.9187	x	x	x	x	4
5	*B*. *c*. *constrictor*	CHUNB53038	Alta Floresta d’Oeste	RO	Brazil	-11.9287	-61.9829	x				5
6	*B*. *c*. *constrictor*	H1293	Porto Velho	RO	Brazil	-8.7649	-63.8683	x	x	x	x	6
7	*B*. *c*. *constrictor*	CHUNB22028	Guajará-Mirim	RO	Brazil	-10.7741	-65.3368	x	x	x	x	7
8	*B*. *c*. *constrictor*	HJ0547	Mutum-Paraná	RO	Brazil	-9.2868	-64.5488	x				8
9	*B*. *c*. *constrictor*	HJ0203	UHE Jirau	RO	Brazil	-9.2582	-64.6498	x	x	x	x	9
10	*B*. *c*. *constrictor*	RVSS33	Nova Ubiratã	MT	Brazil	-13.0357	-55.2599	x	x	x	x	10
11	*B*. *c*. *constrictor*	UFA223	Paranaíta	MT	Brazil	-9.6731	-56.4714	x	x	x	x	11
12	*B*. *c*. *constrictor*	ALT256	Alta Floresta	MT	Brazil	-9.8680	-56.0805	x				12
13	*B*. *c*. *constrictor*	CTMZ05754	Guiratinga	MT	Brazil	-16.3463	-53.7561	x	x	x	x	13
14	*B*. *c*. *constrictor*	CTMZ06126	Itaúba	MT	Brazil	-11.0080	-55.2427	x	x	x	x	14
15	*B*. *c*. *constrictor*	MPEG24581	Belém	PA	Brazil	-1.4436	-48.4410	x	x	x	x	15
16	*B*. *c*. *constrictor*	MPEG21584	Melgaço	PA	Brazil	-1.7977	-50.7385	x	x	x	x	16
17	*B*. *c*. *constrictor*	MNRJ16818	Porto Trombetas	PA	Brazil	-1.4549	-56.3818	x	x	x	x	17
18	*B*. *c*. *constrictor*	FIT5	Santarém	PA	Brazil	-2.4406	-54.7349	x				18
19	*B*. *c*. *constrictor*	FIT8	Santarém	PA	Brazil	-2.4406	-54.7349	x	x	x	x	19
20	*B*. *c*. *constrictor*	BM326	Vitória do Xingu	PA	Brazil	-3.1277	-51.7003	x				20
21	*B*. *c*. *constrictor*	LCBL20	Xambioá	TO	Brazil	-6.4130	-48.5351	x	x	x	x	21
22	*B*. *c*. *constrictor*	QCZAR6476	Orellana (província)		Equador	-6.5826	-77.1986	x				22
23	*B*. *c*. *constrictor*	MNRJ	Falcón (State)		Venezuela	11.1085	-6.9784	x				23
24	*B*. *atlantica* sp. nov.	CHBEZ42	Rio Largo	AL	Brazil	-9.4819	-35.8394	x	x	x	x	24
25	*B*. *atlantica* sp. nov.	IBSP79031	Salvador	BA	Brazil	-12.9731	-38.4871	x	x	x	x	25
26	*B*. *atlantica* sp. nov.	**IBSP79063**	Cachoeiro do Itapemirim	ES	Brazil	-20.8616	-41.1285	x	x	x	x	26
27	*B*. *atlantica* sp. nov.	**MNRJ22936**	Cabo Frio	RJ	Brazil	-22.9218	-42.0412	x	x	x	x	27
28	*B*. *atlantica* sp. nov.	**MNRJ19564**	Rio de Janeiro	RJ	Brazil	-22.9784	-43.3050	x				28
29	*B*. *atlantica* sp. nov.	**MNRJ19740**	Rio de Janeiro	RJ	Brazil	-22.9784	-43.3050	x	x	x	x	29
30	*B*. *atlantica* sp. nov.	MNRJ20700	Teresópolis	RJ	Brazil	-22.4161	-42.9812	x				30
31	*B*. *atlantica* sp. nov.	CHBEZ1196	Caicó	RN	Brazil	-6.4669	-37.0856	x	x	x	x	31
32	*B*. *atlantica* sp. nov.	CHBEZ1254	Serra Negra do Norte	RN	Brazil	-6.6624	-37.3961	x	x	x	x	32
33	*B*. *c*. *amarali*	CTMZ06043	Barra do Garças	MT	Brazil	-15.8534	-52.2704	x	x	x	x	33
34	*B*. *c*. *amarali*	CTMZ00603	Paulínia	SP	Brazil	-22.7650	-47.1489	x	x	x	x	34
35	*B*. *c*. *amarali*	IBSP79064	Esmeraldas	MG	Brazil	-19.8072	-44.1800	x	x	x	x	35
36	*B*. *c*. *amarali*	IBSP84579	Fortaleza de Minas	MG	Brazil	-20.8484	-46.7085	x	x	x	x	36
37	*B*. *c*. *amarali*	IBSP84578	Cabreúva	SP	Brazil	-23.3051	-47.1362	x	x	x	x	37
38	*B*. *c*. *amarali*	IBSP84577	Guarulhos	SP	Brazil	-23.4259	-46.5376	x	x	x	x	38
39	*B*. *c*. *amarali*	IBSP84595	Itú	SP	Brazil	-23.2653	-47.2870	x	x	x	x	39
40	*B*. *c*. *amarali*	MPEG22195	Canaã dos Carajás	PA	Brazil	-6.5300	-49.8532	x	x	x	x	40
41	*B*. *c*. *amarali*	MJJS270	Paraúna	GO	Brazil	-16.9680	-58.3416	x				41
42	*B*. *c*. *amarali*	ESTR00015	Carolina	MA	Brazil	-7.3354	-47.4640	x				42
43	*B*. *c*. *amarali*	CHUNB44537	Buritizeiro	MG	Brazil	-17.3598	-44.9731	x	x	x	x	43
44	*B*. *c*. *amarali*	MNRJ20989	Corumbá	MS	Brazil	-19.0079	-57.6519	x	x	x	x	44
45	*B*. *i*. *imperator*	QCZAR5636	El Mango (região)	--	Equador	-2.45232	-79.62156	x				45
46	* *B*. *occidentalis*	IBSP83333	--	--	Argentina and Paraguay	--	--	x	x	x	x	46
47	* *B*. *occidentalis*	LCBL21	--	--	Argentina and Paraguay	--	--	x	x	x	x	47
48	* *B*. *occidentalis*	LCBL23	--	--	Argentina and Paraguay	--	--	x	x	x	x	48

IBSP = Instituto Butantan (São Paulo, SP, Brazil); CHUNB = Coleção Herpetológica—Universidade de Brasília (Brasília, Brazil); CHBEZ = Coleção Herpetológica do Departamento de Botânica, Ecologia e Zoologia—UFRN (Natal, Rio Grande do Norte, Brazil); CORBIDI = Centro de Ornitologia e Biodiversidad (Lima, Peru); FIT = Faculdades Integradas do Tapajós (Santarém, PA, Brazil); MBIB = Museu Biológico—Instituto Butantan (São Paulo, SP, Brazil); MNRJ = Museu Nacional (Rio de Janeiro, RJ, Brazil); MPEG = Museu Paraense Emílio Goeldi (Belém, PA, Brazil); QCZAR = Museo de Zoología, Pontificia Universidad Católica del *Ecuador*; UVM = Universidade de Medicina Veterinária (Viena, Áustria). MJJS = sample from Laboratório de Ecologia e Evolução, LEEV. LCBL were collected by Lorena Corina Bezerra de Lima; IBSP83333, LCBL21, LCBL23 from Instituto Butantan and Criadouro Jibóias Brazil were identified as *B*. *occidentalis*. ALT-256, RVSS-33, UFA 223: Donated by Dr. Felipe Franco Curcio, UFMT

* Approximate coordinates.

**Fig 1:**
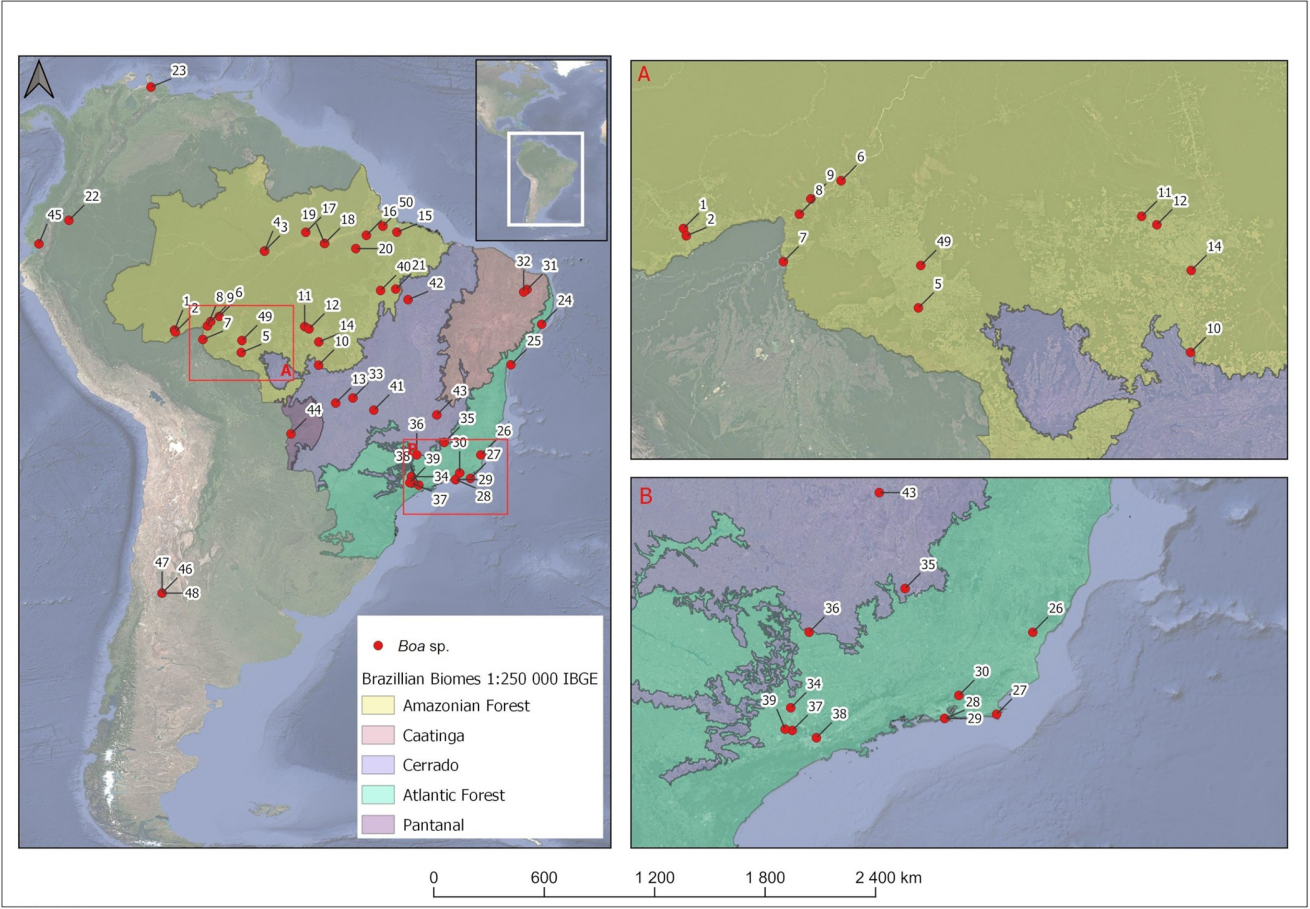
Map showing the locations of origin of the specimens whose sequences were used for phylogenetic analyses in this study (see also Tables 1 and 2, Figs 2 and S1). A) sampling in western Brazil; B) sampling in southeastern Brazil.

Genes amplified for each sample: cyt-*b* = cytochrome b; ND4 = NADH dehydrogenase subunit 4; NTF3 = neurotrophin 3; ODC = ornithine decarboxylase. The last column refers to the location number from where the samples were collected (which correspond to same number in the map of Fig 1). See material and methods for the rationale of identifications to new sequences based on voucher specimens. The paratypes of the new species are marked in bold.

Genomic DNA was extracted from muscle, liver, scales and shed skin (ecdysis) using Chelex 5% following Walsh *et al*. [48], with some minor adaptations. Some scales and shed skin were obtained from live animals by non-invasive techniques (according to the Animal Ethics Committee from the Instituto Butantan Protocol #1148/13). We used two mitochondrial genes, cytochrome b oxidase gene (*cyt-b*) and NADH dehydrogenase subunit 4 (*nd4*), and two nuclear markers, neurotrophin 3 gene (*ntf3*) and nuclear intron of the ornithine decarboxylase gene (*odc*) as molecular markers. We amplified the mitochondrial gene cyt *b* with the same primers described by Hynkovà et al. [30]: L14910 (GACCTGTGATMTGAAAACCAYCGTTGT) and H16064 (CTT TGG TTT ACA AGA ACA ATG CTT TA), based on Burbrink et al. [49]. The NADH gene was amplified using primers ND4 (CACCTATGACTACCAAAAGCTCATGTAGAAGC) and LEU (CATTACTTTTACTTGGATTTGGACCA) [50]. The NTF3 primers were NTF3 F3 (ATATTTCTGGCTTTTCTCTGTGGC) and NTF3 R4 (GCGTTTCATAAAAATATTGTTTGACCGG) [8], and for ODC we used OD-F (GACTCCAAAGCAGTTTGTCGTCTCAGTGT) and OD-R (TCTTCAGAGCCAGGGAAGCCACCACCAAT) [8]. See S1 File for detailed amplification protocols. The fragment sizes amplified are about 744 bp for cyt-*b*, 640 bp for ND4, 590 bp for NTF3 and 500 bp for ODC. In this study, we produced a total of 153 partial sequences, being 48 referred to cyt-*b*, 35 to ND4, 35 to NTF3, and 35 to ODC (Table 1). We also used 30 cyt*b* sequences obtained from the National Center for Biotechnology Information **(**NCBI)—GenBank—from *Boa* spp., including four sequences to compose the outgroup: *Charina bottae*, *Corallus hortulana*, *Epicrates cenchria*, and *Eunectes murinus* (Table 2, Fig 1).

**Table 2:** Sequences accessed and downloaded from GenBank. Morphotype identification, voucher or collection number in the field, locality, country of origin, and geographical coordinates. Genes accessed from NCBI: Cytochrome-*b* (cyt-*b*). See material and methods for the rationale of re-identifications to GenBank sequences without examination of voucher specimens.

Sample	Taxa	Voucher	Locality	FU	Country	Latitude	Longitude	Cyt-*b*
1	*B*. *c*. *constrictor*	KJ621415	Nova Brasília	Rondônia	Brazil	-11.150	-61.57	x
2	*B*. *c*. *constrictor*	EU273654	Ilha de Marajó	Pará	Brazil	-0.898*	-49.801*	x
3	*B*. *c*. *constrictor*	GQ300902	Iquitos	Loreto	Peru	-2.540	-73.226	x
4	*B*. *c*. *constrictor*	KX150374	--	--	Venezuela	6.423*	-66.589*	x
5	*B*. *c*. *constrictor*	EU273629	--	Guyana	Guyana	4.872*	-58.951*	x
6	*B*. *c*. *constrictor*	EU273630	--	--	Suriname	4.058*	-55.885*	x
7	*B*. *i*. *imperator*	KJ621416	Alamos	"Sonora"	Mexico	29.212	-110.136	x
8	*B*. *i*. *imperator*	KJ621419	Acaponeta	Sinaloa	Mexico	22.351	-103.314	x
9	*B*. *i*. *imperator*	KJ621422	Manzanillo	Colima	Mexico	19.101	-104.295	x
10	*B*. *i*. *imperator*	KJ621431	Mascota	Jalisco	Mexico	-20.204	-104.284	x
11	*B*. *i*. *imperator*	KJ621470	Huautla	Morelos	Mexico	18.448	-98.987	x
12	*B*. *i*. *imperator*	KJ621507	Cuxtal	Yucatán	Mexico	20.911	-89.611	x
13	*B*. *i*. *imperator*	EU273616	Cancún	Quintana Roo	Mexico	21.157*	-86.886*	x
14	*B*. *i*. *imperator*	KJ621529	Paraíso	Panama	Panama	9.031	-79.610	x
15	*B*. *i*. *imperator*	KJ621534	San Francisco Menéndez	Ahuachapán	El Salvador	13.867	-89.983	x
16	*B*. *i*. *imperator*	KX150377	--	Crawl Cay	Belize	16.599	-88.219	x
17	*B*. *i*. *imperator*	KX150381	--	Baja Verapaz	Guatemala	15.078*	-90.412*	x
18	*B*. *i*. *imperator*	KX150399	--	--	Honduras	15.199	-86.241	x
19	*B*. *i*. *imperator*	KX150402	--	--	Nicaragua	12.865	-85.207	x
20	*B*. *i*. *ortonii*	KF576739**	--	--	--	--	--	x
21	*B*. *i*. *sabogae*	EU273665	--	Saboga Island	Panama	8.622**	-79.06**	x
22	*B*. *i*. *longicauda*	EU273612	--	--	--	--	--	x
23	*B*. *nebulosa*	KF576731**	--	--	--	--	--	x
24	*B*. *occidentalis*	EU273651	--	--	Argentina	-34.999*	-64.923*	x
25	*B*. *occidentalis*	GQ300916	--	--	Argentina	-34.999*	-64.923*	x
26	*B*. *orophias*	KF576734**	--	--	--	--	--	x
27	*Charina bottae* *	AY099986	--	--	--	--	--	x
28	*Corallus hortulana* *	HM348868	--	--	--	--	--	x
29	*Epicrates cenchria* *	HQ399501	--	--	--	--	--	x
30	*Eunectes murinus* *	U69808	--	--	--	--	--	x

Samples EU, GQ = Hynkovà et al. [30]; KJ = Suárez-Altilano et al. [32]; KX = Card et al. [11]

*Outgroup

**Unpublished data.

Samples were identified by the examination of museum vouchers from which they were obtained. Additionally, samples that were taken from live specimens (e.g., ecdyses) were also checked for identification. For analytical purposes, the samples were considered at the subspecific level, whether they were previously identified or after our double-checking for the identification accuracy.

Regarding the GenBank sequences, the originally given number and identification was maintained in order to make repeatability easier, except in the situations detailed below. Thus, samples present in this study comprised nine of the currently recognized taxa [34]: *Boa constrictor amarali*, *B*. *c*. *constrictor*, *B*. *i*. *imperator*, *B*. *i*. *longicauda*, *B*. *i*. *ortonii*, *B*. *i*. *sabogae*, *B*. *nebulosa*, *B*. *occidentalis* and *B*. *orophias* (Tables 1 and 2).

GenBank sequences from *Boa occidentalis* were not checked for identification accuracy, since their morphological and molecular features are unambiguous (see results). The sequences EU273629 (Guyana) and EU273630 (Suriname) downloaded from GenBank were identified as *Boa constrictor constrictor* by Hynkovà *et al*. [30], however they are catalogued as *Boa constrictor imperator* in the GenBank. Since there is no record of *B*. *c*. *imperator* in the cis-Andean portion of South America, we use the identification *Boa constrictor constrictor* for these two samples, assuming a possible mistake in labelling while uploading the sequences. The sequence EU273612 from *B*. *constrictor longicauda* (here considered as *B*. *i*. *longicauda*) [30] has no exact locality associated with it. Sequence KF576731 from *B*. *nebulosa*; KF576734 from *B*. *orophias*; KF576739 from *B*. *i*. *ortonii*; and EU273665 from *B*. *imperator sabogae* are available on GenBank, and they were checked for identification as they are. Nevertheless, these sequences were used here because they represent taxa for which we have only a few or no additional samples at all. The low number of samples, in some cases, may be due to a supposed restricted endemism and CITES category [51] of these taxa. In the case of *B*. *c*. *nebulosa*, *B*. *c*. *orophias* and *B*. *c*. *sabogae* (here considered as *B*. *nebulosa*, *B*. *orophias*, and *B*. *i*. *sabogae*, respectively), this is more notorious because they are endemic to islands. Finally, for *B*. *c*. *ortonii* and *B*. *c*. *longicauda* (here considered as *B*. *i*. *ortonii* and *B*. *i*. *longicauda*, respectively) we had access to new samples from recently collected vouchers and compared with those from GenBank.

### Phylogenetic analyses and genetic distances

Sequences were edited through CodonCode Aligner 6.0.2 (LI-COR Inc) and MEGA11: Molecular Evolutionary Genetics Analysis version 11 [52]. Multiple alignment of the sequences was performed online in the MAFFT 7 [53]. The alignments were edited to eliminate or minimize gaps. We performed phylogenetic analyses using Maximum Likelihood (ML) and Bayesian inference with two matrices composed of the following datasets: (i) 73 cyt-*b* sequences (43 produced in this study) from samples of Mexico, Central America and South America; and (ii) the second matrix composed of 39 samples from South America, including 35 individuals with the four concatenated markers (cyt-*b*, ND4, NTF3, and ODC produced in this study), plus the outgroup *Charina bottae*, *Corallus hortulana*, *Epicrates cenchria*, and *Eunectes murinus* (with cyt*b* only). The ML analyses were performed using both RaxML [54] on CIPRES, using the GTRCAT evolution model, with 1000 replicates of bootstrap, and also using MEGA11 [52], in which initial tree(s) for the heuristic search were obtained automatically by applying Neighbor-Join and BioNJ algorithms to a matrix of pairwise distances estimated using the Maximum Composite Likelihood (MCL) approach, and then selecting the topology with superior log likelihood value. A discrete Gamma distribution was used to model evolutionary rate differences among sites (2 categories (+G, parameter = 1.6998)). Bayesian analyses using the same two matrices on CIPRES recovered similar topologies to ML analyses. *Boa atlantica* sp. nov. was recovered with posterior probability = 1 for both analyses (S2A and S2B Fig).

Genetic distance analysis was conducted in MEGA11 [52], using the Kimura 2-parameter model [55]. The analysis involved 73 cyt-*b* nucleotide sequences. Codon positions included were 1st+2nd+3rd+Noncoding. All positions with less than 95% site coverage were eliminated, i.e., fewer than 5% alignment gaps, missing data, and ambiguous bases were allowed at any position (partial deletion option). There were 652 positions in the final dataset.

### Morphological analyses and species boundaries

#### Morphological samples and variables

A total of 1088 specimens of all cis-Andean *Boa* spp. were examined for this study (406 *Boa c*. *amarali*, 404 *B*. *c*. *constrictor*, 9 *B*. *c*. *melanogaster*, 31 *B*. *c*. *nebulosa*, 78 *B*. *c*. *occidentalis*, 13 *B*. *c*. *orophias*, and 147 *Boa* sp.). We selected wild-caught specimens for examination, avoiding as much as possible the use of artificial breeds and captive ones. A list of all specimens studied herein and its identification is available in S2 File. The following measurements were taken on the side of each specimen, using a digital calliper, to the nearest 0.01: head length, head height, head width, distance between the eyes, distance between the nostrils, eye-mouth distance, eye-rostral distance, eye diameter, and cloacal spur length. Body length measures were taken with a flexible ruler: snout-vent length (SVL) and tail length (TL). Additionally, we also took the following meristic and categorical characters: Meristic: number of circumorbital scales; dorsal scale rows (anterior, midbody and posterior); gular scales, infralabial scales, intrasupraocular scales, preventral scales, scale rows between the nostrils, scale rows over the preocular stripe, subcaudal scales, subocular scales, supralabial scales, tail dorsal scales rows at the level of first subcaudal, ventral scales; number of dorsal spots; dorsal spot width: number of dorsal scales between the lateral margins of the dorsal spot; dorsal spot length: number of dorsal scales from the anterior margin of the dorsal spot up to its posterior margin; saddle length (number of dorsal scales between two consecutive dorsal spots); number of tail spots. And categorical: longitudinal head stripe shape (continuous or fragmented, with or without lateral projections); posterior limit of longitudinal head stripe (contacts first dorsal saddle or not); postocular stripe limits (contacts first dorsal saddle or not); dorsal colour and pattern; dorsal spot shape; progressive change in dorsal spots towards the tail; ventral colour and pattern; progressive ventral darkening towards the tail; colour of lateral ocelli; colour of tail spots; presence of tail interspots (fuzzy spots between two tail spots); cloacal blotch.

We prepared hemipenes according to protocols described by Pesantes [56], with modifications proposed by Zaher & Prudente [57]. In order to highlight hemipenial ornamentations, the organs were immersed in a solution of alizarin red and 70% ethanol during five minutes [58]. Subsequently, hemipenes were filled with a homogeneous mixture of coloured petroleum jelly to increase the contrast against the dyed calcified structures [59]; and the hemipenes were then finally stored in 70% ethanol. Hemipenial caracters follow [60–62]: hemipenial shape, sulcus spermaticus split site, sulcus spermaticus orientation, lateral projections of the margins of sulcus spermaticus, ornamentation on the base, body, lobes and apex of the hemipenis, and number of flounces.

We adopted here the General Lineage Concept of Species [75], considering species as unique evolving metapopulations lineages. We search for concordance between the topology of preferred molecular hypothesis retrieved and phenotype evidences from discrete and continuous characters, since some features such as colour patterns, morphometrics and hemipenial morphology are likely uncorrelated with each other. The correspondence among these data sources might represent independent evidence for robust species boundaries. Therefore, we consider presence of one or more exclusive diagnostic character, which distinguishes a putative taxon from the others in the *Boa constrictor* Clade, as species delimitation criteria.

#### Nomenclatural acts

The electronic edition of this article conforms to the requirements of the amended International Code of Zoological Nomenclature, and hence the new names contained herein are available under that Code from the electronic edition of this article. This published work and the nomenclatural acts it contains have been registered in ZooBank, the online registration system for the ICZN. The ZooBank LSIDs (Life Science Identifiers) can be resolved and the associated information viewed through any standard web browser by appending the LSID to the prefix “http://zoobank.org/”. The LSID for this publication is: urn:lsid:zoobank.org:pub:4FEE3F5C-9213-470D-9877-79AB8FF8D4D0. The electronic edition of this work was published in a journal with an ISSN, and has been archived and is available from the following digital repositories: LOCKSS.

## Results

### Molecular analyses

As the main aim of this study was to investigate the boas distributed along to the Atlantic Forest, the cyt-*b* matrix constructed herein has a reduced number of representatives from Mexico and Central America, although we included *B*. *imperator imperator*, *B*. *i*. *longicauda*, *B*. *i*. *saboggae*, and *B*. *i*. *ortonii* in our analyses (Tables 1 and 2). The topologies of cyt-*b* and the four-genes matrices were not completely concordant, although the same groups were recovered, and therefore the relationships between groupings will not be approached here.

**Fig 2:**
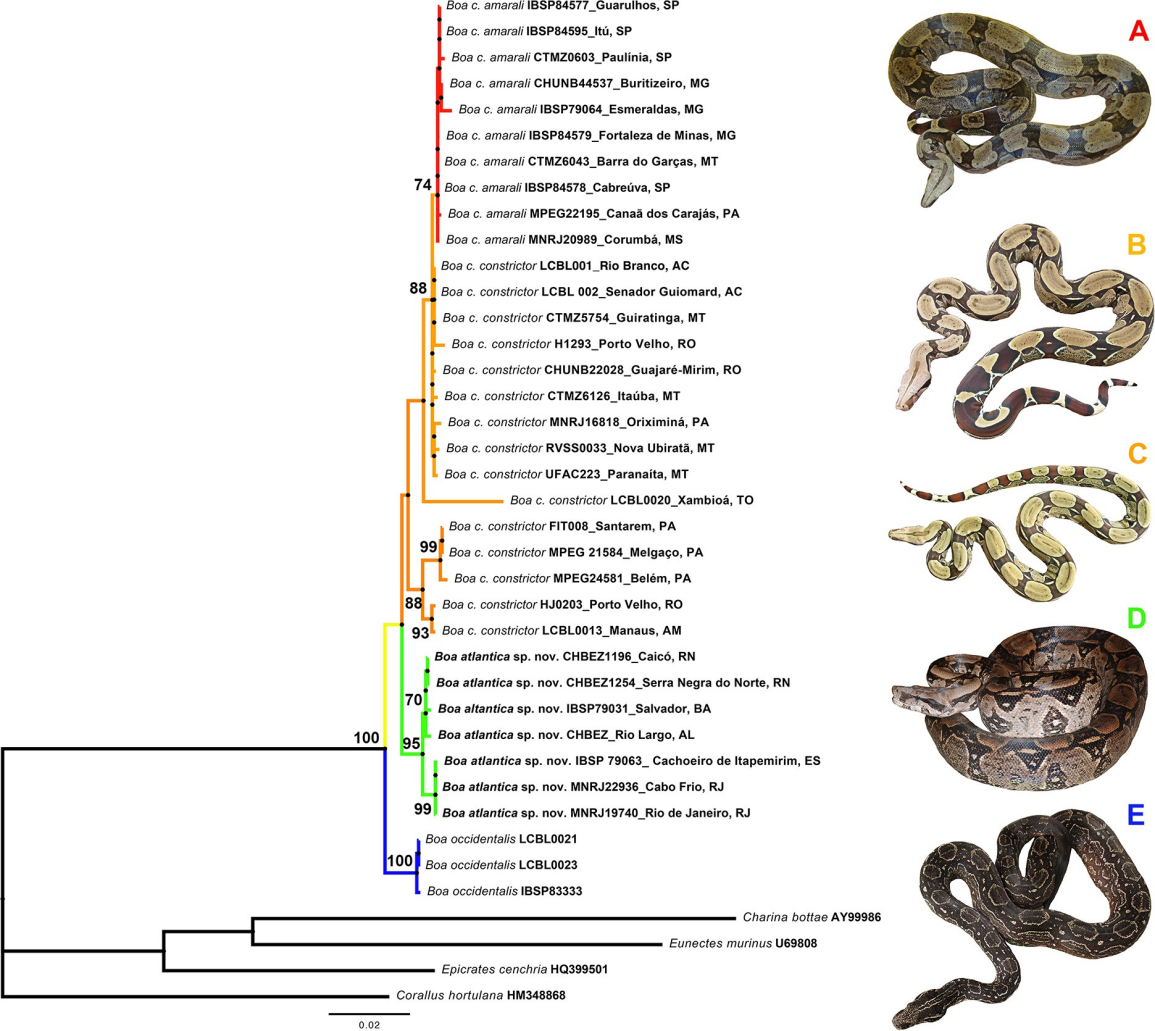
Phylogenetic tree inferred by using Maximum Likelihood and general time reversible model. The matrix is composed of 39 samples from South America, being 35 samples with the four concatenated markers (cyt-*b*, ND4, NTF3, and ODC resulting in 2305 positions in the final dataset) and the outgroup (*Charina bottae*, *Corallus hortulana*, *Epicrates cenchria*, and *Eunectes murinus*) with cyt*b* only). The percentage of trees in which the associated taxa clustered together is shown above the branches (bootstrap). The tree is drawn to scale, with branch lengths measured in the number of substitutions per site. Group colours: Black: Outgroup; blue: *B*. *occidentalis*; green: *B*. *atlantica* **sp. nov.**; orange: *B*. *constrictor* and red: *B*. *amarali*. Photographs: A) *B*. *c*. *amarali*, no locality; *B*. *c*. *constrictor*, no locality, Brazil; C) *B*. *c*. *constrictor*, from Cuiabá, Mato Grosso, Brazil; *B*. *atlantica* sp. nov., from Rio de Janeiro, Brazil; *B*. *occidentalis* from Tucumán, Argentina.

*Boa* was recovered as a monophyletic group with high support values in both analyses with cyt-*b* sequences only (bootstrap = 100%) (S1 Fig) and the concatenated matrix with the four genes (bootstrap = 100%) (Fig 2). The evolutionary history in the latter analysis was inferred by using only individuals from South America, in which *B*. *occidentalis*, in blue, (bootstrap = 100%) was recovered as the sister group to the other individuals, although the support is < 70% (52%). *Boa atlantica* **sp. nov.** (Fig 2 - in green) was recovered with a high support (95%) with two subclades: one composed of representatives from Northeastern Brazilian States (Alagoas—AL, Bahia—BA, and Rio Grande do Norte—RN) (70%), and the other one composed of individuals from Southeastern States (Rio de Janeiro—RJ, and Espírito Santo—ES) (99%). *B*. *c*. *constrictor* was not recovered as a monophyletic group (orange): individuals from the same Brazilian States (Pará and Rondônia) were recovered in two different groups—one recovered with 88%, and the other one was recovered as the sister to *B*. *c*. *amarali* with a very low support (<70%). A group encompassing *B*. *c*. *amarali* was recovered with a moderate support (74%) (in red).

The analysis using cyt-*b* recovered six main lineages based on the dataset used, and weak support values defined the relationships among them (bootstrap < 50%): *B*. *imperator* (S1 Fig—in grey) with support of 74%; *B*. *occidentalis* (blue) with 96%; one group (52%) composed of *B*. *c*. *constrictor* (orange) from Peru, Equador, Guyana, Suriname, Venezuela, and Brazil (Pará –PA, and Rondônia—RO states– 70%), which is the sister group (54%) to *B*. *c*. *constrictor* + *B*. *c*. *amarali* (in orange) from different Brazilian States and biomes; *B*. *atlantica* **sp. nov.** (green) was recovered with bootstrap of 94%; and *B*. *orophias* and *B*. *nebulosa* (pink) were recovered as closely related in a clade with support of 77%. It is worth highlighting that due to the availability of sequences in each matrix, the groups composition was not the same in the analyses with the four concatenated markers.

Genetic distances showed the main supported lineages with distances of 3 and 4% (see S3 Fig): *B*. *atlantica* **sp. nov.** diverged in 3 or 4% of *B*. *c*. *amarali*, *B*. *c*. *constrictor* (from different localities and phytophysiognomies), *B*. *occidentalis*, and *B*. *nebulosa*; 4 or 5% of *B*. *orophias*; and 6 to 9% of *B*. *imperator* (including the subspecies *B*. *i*. *imperator*, *B*. *i*. *ortonii*, *B*. *i*. *sabogae*, and *B*. *i*. *longicauda*). The same occurred with the other lineages, except *B*. *c*. *amarali* and *B*. *c*. *constrictor* in which the distances ranged from 0 to 3%.

### Taxonomy

#### *Boa atlantica* sp. nov

Gonzalez, Lima, Passos & Silva urn:lsid:zoobank.org:act:242E539F-CF4C-4C0B-A5E1-D4E143BE82A5.

*Constrictor constrictor constrictor–*Ihering, 1911 [63] (part.); Amaral, 1930 [64] (part.); Stull, 1932 [65] (part.); Stull, 1935 [66] (part.);

*Boa constrictor constrictor*–Forcart, 1951 [27] (part.); Stimson, 1969 [67] (part.); Peters & Orejas-Miranda, 1970 [68] (part.);

*Constrictor constrictor constrictor–*Amaral, 1977 [69] (part.);

*Boa constrictor–*Henderson *et al*., 1995 [6] (part.);

*Boa constrictor constrictor–*McDiarmid *et al*., 1999 [25] (part.);

*Boa constrictor–*Marques *et al*., 2001 [70]; Argôlo, 2004 [71]; Tipton, 2005 (part.) [72];

*Boa constrictor constrictor–*Bonny, 2007 (part.) [3];

*Boa constrictor–*Pontes & Rocha, 2008 (part.) [73]; Hynkovà *et al*. 2009 [30] (part.); Reynolds *et al*., 2014 [35] (part.); Wallach *et al*., 2014 [74] (part.); Card *et al*., 2016 [11] (part.);

*Boa constrictor constrictor*–Reynolds & Henderson, 2018 [34] (part.);

*Boa constrictor–*Nogueira *et al*., 2019 [9] (part.).

#### Holotype

Brazil: Rio de Janeiro, Fundação Osório, Rio Comprido, Rua Paula Ramos, 52 (22°56’03.0"S 43°12’36.9"W, datum WGS84; 93 m above sea level, hereafter asl), MNRJ 27242, adult male, collected by Sergeant Marco Aurélio da Silva on 13 September 2019. The holotype was collected on a tree, while copulating with the paratype MNRJ 27243.

#### Paratypes (n = 47)

All from Brazil: Alagoas State: Passo do Camaragibe (9°16’28.5"S, 35°28’04.8"W), 44 m asl, MNRJ 3940, female, collected by H. Silva at Passo on 18 January 1988; Bahia State: Ilhéus, CEPEC-CEPLAC (14°45’21.6"S, 39°13’53.4"W), 58 m asl, CZGB 4862, female, collected by G.A. Costa on 10 July 1996; Salobrinho, Ilhéus (14°47’S, 39°10’W), 29 m asl, MNRJ 6362, male, collected by S. Rangel in September 1987; Ituaçú (13°48’S, 41°18’W), 539.95 m asl, MNRJ 6361, female, collected by U. Caramaschi & H. Niemeyer on 07 August 1997; Espírito Santo state: Cachoeiro do Itapemerim (20°51’00.4"S, 41°06’42.9"W), 35 m asl, IBSP 79063, female, collector unknown, collected on 10 January 2011; Setiba, Guarapari (20°37’S, 40°26’W), 20 m asl, MNRJ 23361, female, collected by T.S. Soares, date of collection unknown and MNRJ 24903, male, collected by C.F.D. Rocha on 15 November 1999; Reserva Biológica de Comboios, Linhares (19°33’S, 40°03’W), 13 m asl, MNRJ 23879, male, collected by A.P. Almeida on 31 August 2007; São Mateus, Campus CEUNES/UFES (18°40’S, 39°51’W), 8 m asl, MNRJ 23882, male, collected by R.S. Bérnils on 15 March 2011; Vitória, Morro da Gamela (20°17’50.1"S, 40°18’05.5"W), 17 m asl, MNRJ 9565, male, collected by J.L. Gasparini, & F. Campagnolli on 14 January 2002; Rio de Janeiro state: Cabo Frio (22°50’S, 41°59’W), 6 m asl, MNRJ 22936, female, collected by R.R. Pinto et al on 03 May 2012; Carapebus, Parque Nacional da Restinga de Jurubatiba (22°15’S, 41°39’W), 8 m asl, MNRJ 26802, male, collected by D.S. Fernandes et al. on the 8 March 2018; Guapimirim (22°34’S, 43°0’W) 23 m asl, MNRJ 14238, female, collector unknown, collected in May 2001; Guapimirim (22°34’S, 43°0’W) 23 m asl, MNRJ 14250, female, collector unknown, collected in May 2001; Iguaba Grande (22°50’S, 42°10’W), 37 m asl, MNRJ 17353, female, date of collection and collector unknown; Itaboraí, Complexo Petroquímico do Rio de Janeiro (COMPERJ) (22°39’S, 42°51’W), 21 m asl, MNRJ 25057, female, collected by J. Creusen on 03 June 2013 and MNRJ 26324, male, collected on 28 July 2016; Itaboraí (22°39’S, 42°51’W), 134 m asl, MNRJ 25413, female, date of collection and collector unknown; Maricá (22°55’S, 42°51’W), 12 m asl, MNRJ 13111, female, collected by C.L. Prata on 12 May 2005; Itaipu, Niterói (22°57’S, 43°02’W), 7 m asl, MNRJ 11205, female, collected by F. Vieira, J.V. Camargo, & M.A. Gonçalves in May 2004; Pendotiba, Niterói (22°54’S, 43°04’W), 111 m asl, MNRJ 23573, female, collected by M.S.C. Mesquita on 19 January 2013; Niterói (22°57’S, 43°02’W), 2 m asl, MNRJ 16922, female, collected on 17 July 2008, by R.W. Kisling; Nova Iguaçu (22°42’S, 43°28’W), 34 m asl, MNRJ 24860, male, collected by A. Antunes on 13 June 2013; and MNRJ 26213, female, collector unknown, collected in 2014; Porciúncula, Fazenda Vargem Alegre (20°58’S, 42°02’W), 231 m asl, MNRJ 14172, female, collected by B. Pimenta on 08 March 2006; Rio das Ostras (22°30’S, 41°56’W), 31 m asl, MNRJ 10117, female, collected by D.S. Fernandes et al. on 17 November 2003; Rio de Janeiro, Aeroporto Internacional Tom Jobim (22°48’S, 43°15’W), 7 m asl, MNRJ 25952, female, collected by J.T. Baldine in November 2015 and MNRJ 26350, female, collector unknown, collected on 9 May 2016; Água Santa (22°54’19.0"S, 43°18’26.1"W), 221 m asl, MNRJ 25950, female, collected by D.S. Fernandes, B. Miranda, & P. Pinna 09 December 2015; Bairro Jardim Botânico, Horto Grotão (22°57’S, 43°14’W), 650 m asl, MNRJ 19740, male, collected by L. Caetano on 13 July 2010; Cosme velho (22°56’S, 43°11’W), 18 m asl, MNRJ 19412, male, collected by J.P. Pombal on 05 April 2010 and MNRJ 19564, male, collected on 18 May 2010; Del Castilho (22°52’S 43°16’W), 22 m asl, MNRJ 25953, female, collected by R. Baptista on 30 January 2016; Estrada do Rio Morto (23°00’S, 43°29’W), 3 m asl, MNRJ 23144, female, by R.L. Santos, date of collection unknown; Estrada dos Mananciais (22°55’S, 43°22’W), 21 m asl, MNRJ 26589, female, collected by D. B. Santos on 21 April 2017; Ilha do Governador (14°47’52.6"S, 39°10’35.3"W), 15 m asl, MNRJ 9449, female, collector unknown, collected on 21 March 2001 and MNRJ 26585, female, collected by A. Carneiro on 6 June 2017; Parque Nacional da Tijuca (22°56’S, 43°17’W), 360 m asl, MNRJ 26886, female, collected by C. Bueno on 13 March 2018 and MNRJ 27262, female, same collector, collected on 29 February 2019; Recreio dos Bandeirantes (23°01’S, 43°28’W), 14 m asl, MNRJ 14200, female, collected on by J.R. Gomes 27 May 2006 and MNRJ 14201, female, same collector, collected on 13 May 2006; Serra do Mendanha (22°50’S, 43°29’W), 190 m asl, MNRJ 17547, female, collected by J.A.L. Pontes, date of collection unknown; Rio de Janeiro (22°57’S, 43°18’W), 10 m asl, MNRJ 10092, male, collected by M. Mocelin, date of collection unknown and MNRJ 26796, male, collector unknown, collected on 22 August 2017; São Francisco de Itabapoana (21°14’03.0"S, 41°07’19.0"W), 4 m asl, MBML 2097, male, collected by G.L. Forreque on 10 October 2006; Teresópolis, road BR-116 km 86.5 (22°24’S, 42°58’W), 972 m asl, MNRJ 20700, female, collector unknown, collected on 25 July 2011; Fundação Osório, Rio Comprido, Rua Paula Ramos, 52 (22°56’03.0"S 43°12’36.9"W, WGS84), 93 m asl, MNRJ 27243, adult female, collected by Sgt. Marco Aurélio da Silva on 13 September 2019 [copulating with the holotype MNRJ 27242].

#### Diagnosis. *Boa atlantica* sp. nov

can be distinguished from the other congeners by the following unique combination of characters: (i) ventrals 228–243; (ii) subcaudals 47–58 in males, and 31–56 in females; (iii) anterior dorsal scale rows 51–66; (iv) midbody dorsal scale rows 69–90; (v) posterior dorsal scale rows 42–54; (vi) dorsal body spots 17–23; (vii) tail spots 4–6 in males, 2–6 in females; (viii) longitudinal head stripe usually continuous; (ix) head stripe without lateral projections; (x) elliptical, circular or double-oval dorsal interspots; (xi) posterior dorsal interspots not blotched; (xii) no change in dorsal spots towards the tail; (xiii) lateral ocelli dark brown, black or faint reddish, white bordered; (xiv) belly cream with tones of orange, brown and black, scattered of black dots and large groups of black spots; (xv) belly with progressive darkening towards the tail; (xvi) black spots on ventral surface of tail.

#### Etymology

The species epithet *atlantica* is a Latin adjective that refers to the Atlantic coast but mainly the Atlantic Forest ecoregion, the homeland of several endemic species, including this new *Boa*. The preservation of this biome is crucial for conservation, nonetheless, it has been suffering deforestation since the colonial times and only 12% of it remains standing. This situation is under serious threat since the past Brazilian policies lack empathy for conservation issues and seem to foster deforestation even further.

#### Description of the holotype

**Fig 3:**
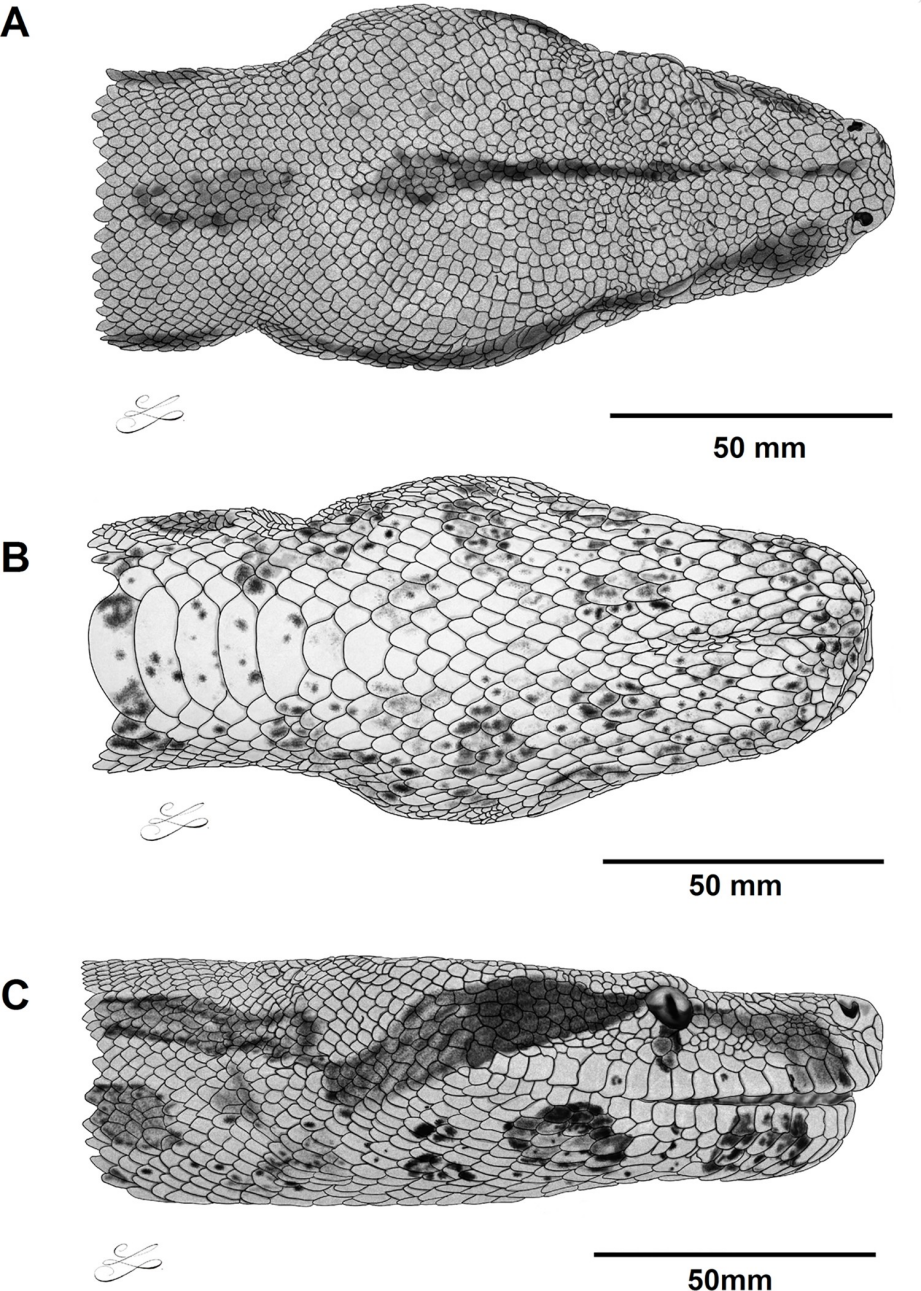
Dorsal (A), ventral (B), and lateral (C) views of the head of the holotype of *Boa atlantica* **sp. nov.** (MNRJ 27242) from Rio de Janeiro, Atlantic coast of Brazil.

**Fig 4:**
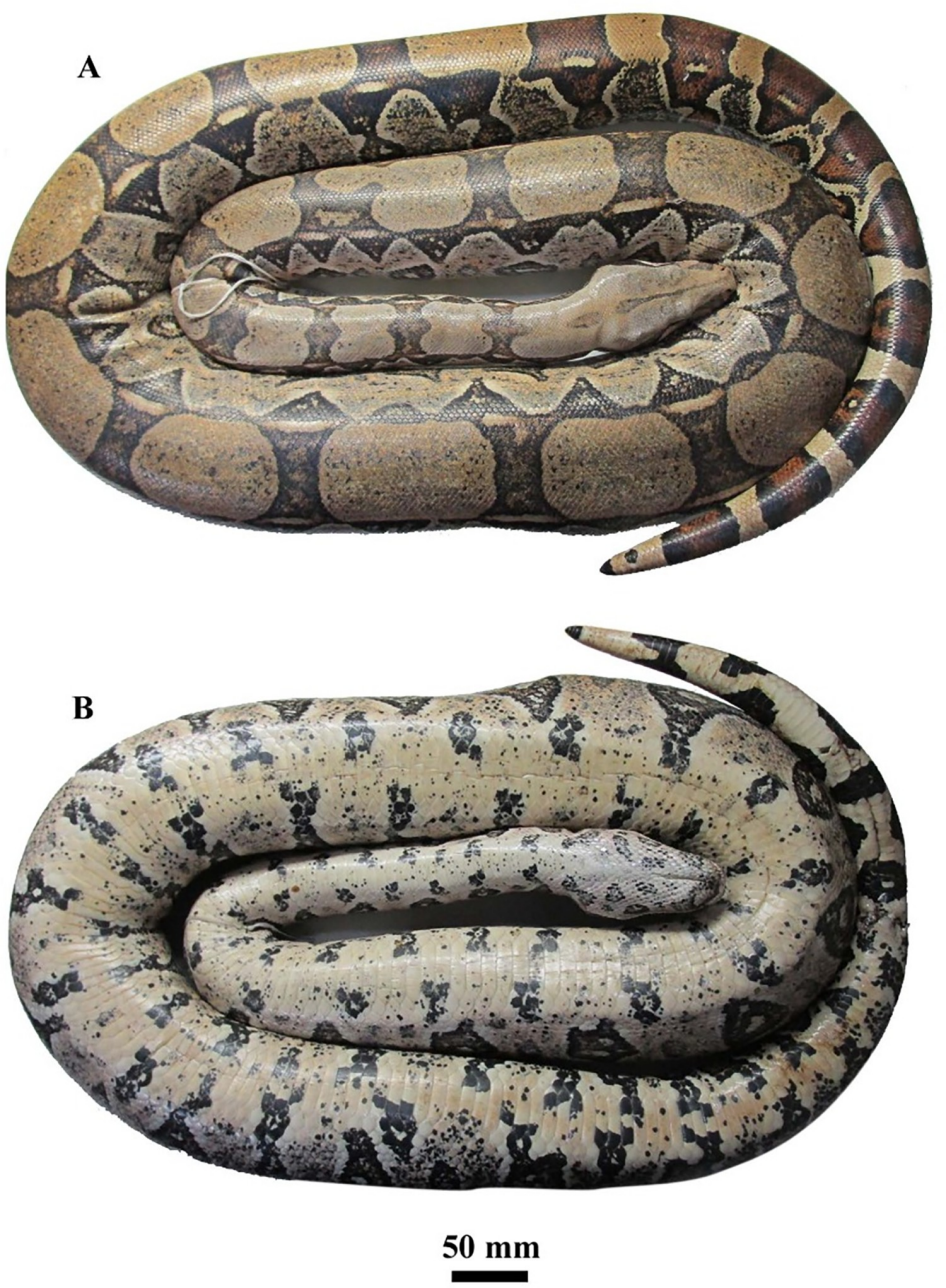
Dorsal (A) and ventral (B) views of the holotype of *Boa atlantica* **sp. nov.** (MNRJ 27242) from Rio de Janeiro, Atlantic coast of Brazil.

MNRJ 27242 (Figs 3 and 4), adult male, SVL 2016 mm, TL 294 mm, head width 27.5 mm, head length 77.9 mm, head height 27.5 mm, distance between eyes 22.9 mm, eye-rostral distance 26.3 mm, eye-mouth distance 8.2 mm, distance between nostrils 12.3 mm, cloacal spur length 7.3/6.8 mm, eye height 6.2 mm, eye length 6.5 mm, scales over the preocular stripe 9/7, circumorbitals 17/15, suboculars 1/2, supralabials 22/21, infralabials 26/26, scales between nostrils 6, intrasupraoculars 17, gulars 18/17, dorsal scale rows 64/90/50, tail dorsal scale rows 24, preventrals 2, ventrals 245, subcaudals 57. Dorsum of head light brown light brown with discrete dark grey speckles; longitudinal head stripe brown, bordered by dark brown and black, extending from internasal region, breaking in occipital region, and continued up to cervical region; lateral head surface light brown; preocular stripe faded brown; and subocular stripe brown marginally reaching supralabials; postocular stripe dark brown, connecting with first dorsal saddle; some supralabials with dark brown dots; head ventral surface cream, with grey and salmon speckles and black dots; black dots unite forming two larger pairs of infralabial spots, and another three pairs of gular spots; dorsal background light brown progressively darkens towards tail; saddle-shaped dorsal spots, 22, laterally connected to each other, delimiting oval spots, dark brown in 2/3 of body, closer to each other and brownish-red before tail; body, lateral surface 2/3 of body greyish cream with salmon speckles and dark brown ocelli, encircling creamish white spots; last body third cream, with black speckles and irregular black ocelli encircling brick-red spots; ventral body surface cream, with black speckles and larger black blotches united on the sides, almost in alternate way; salmon tones in the midline of belly; dorsal background of tail yellow with five brick-red spots, bordered by black; first three spots with a pair of yellowish ocelli; ventral surface of tail cream, with alternate black spots among lateral sides of subcaudals not contacting each other.

**Fig 5:**
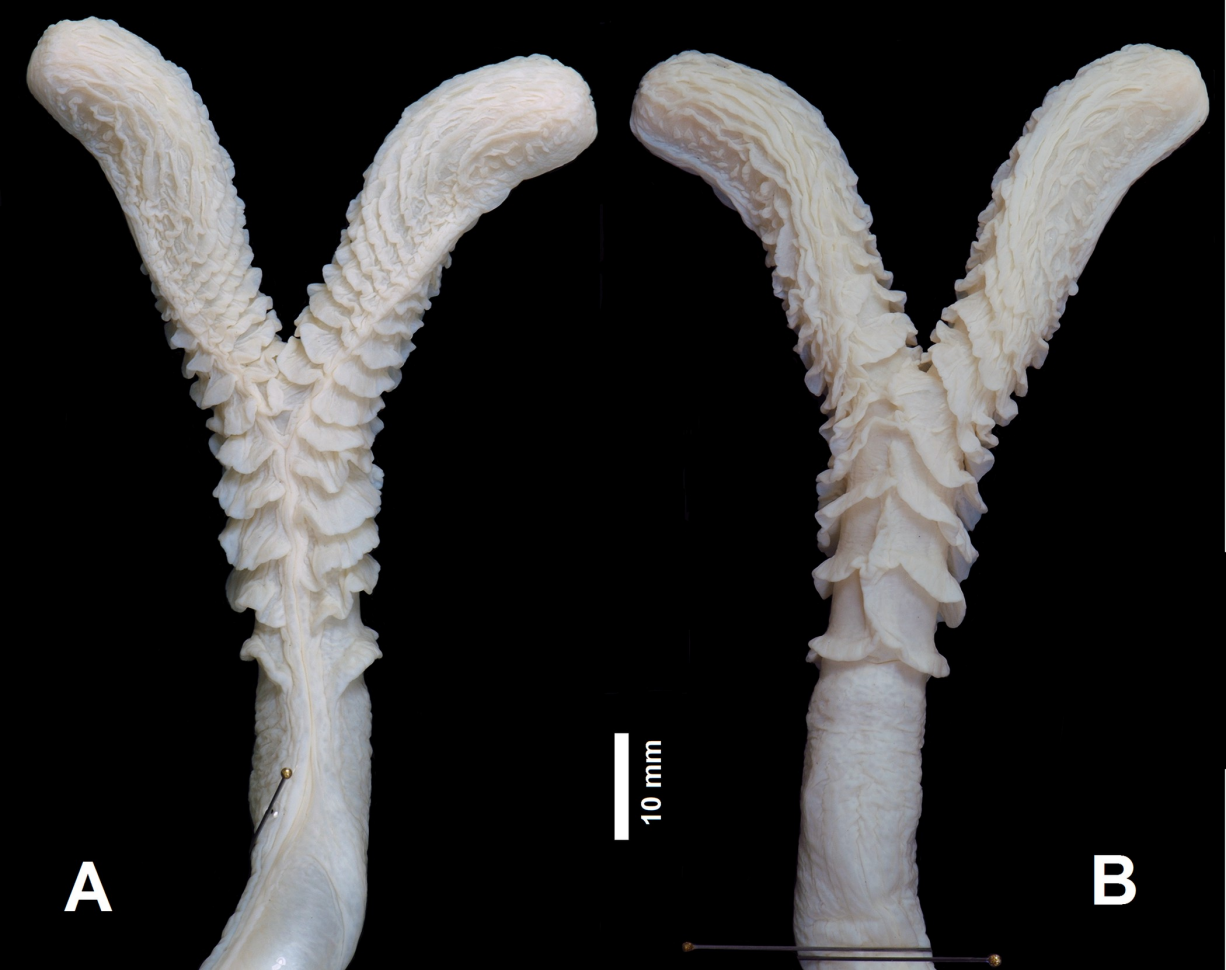
Sulcate (A) and asulcate (B) sides of hemipenis of the holotype of *Boa atlantica* **sp. nov.** (MNRJ 27242) from Rio de Janeiro, Atlantic coast of Brazil.

Hemipenis of the holotype: fully everted and maximally expanded hemipenis renders a bilobed, non-capitate, and non-calyculated organ; lobes relatively short and sub-cylindrical with rounded apices, similar size and oriented centrifugally; lobes covered by 6 transversal flounces on its basal portion; lobes naked from median to apical region; sulcus spermaticus divides on distal portion of organ; sulcus spermaticus branches centrifugally oriented running to tip of lobes; margins of sulcus spermaticus bordered by flounces on basal to most of distal portion of lobes; sulcus spermaticus expanded at apices of lobes; hemipenial body subcylindrical; distal region of hemipenial body defined by transversal series of 4 transversal flounces connected to the sulcus spermaticus; proximal region of hemipenis naked (Fig 5).

#### Variation

We refer to Tables 3 and S1 for the synthesis of quantitative variation of *Boa atlantica*. Dorsal ground colour from light to pinkish brown; flanks on the first two thirds of body lighter than dorsum, usually with grey tones; flanks darker on last third of body, usually with tones of blackish brown; longitudinal head stripe darker than background colouration, usually with brown; head stripe usually continuous, with a brown blotch of the same colour as the body; longitudinal head stripe without lateral projections, from internasal region, usually extending to occipital region; preocular and subocular pigmented with tiny brown dots; last two stripes lighter than postocular or with same colour; postocular stripe extends to quadrate-mandibular articulation or connects to first dorsal saddle; usually no gular blotch; dorsal spots 17–23, elliptical, circular or double-oval, not blotched; dorsal spots (13–28 scales long, 8–17 scales wide) light brown to pinkish brown, bordered by brown; posterior dorsal spots do not change in colour or form; saddles dark brown or black (4–13 scales long), bordered by light brown to pinkish brown, usually connected to each other; dorsal saddles usually with brown on last body third; lateral ocelli from dark red, brownish orange to blackish brown, bordered by cream; belly cream with pinkish tones and black blotches; ventral black blotches progressively increase in number and size towards last body third (= posterior ventral darkening); dorsal tail colour light brown, with 3–6 dorsal brown or black spots, without tail interspots; ventral surface of tail cream with spots generally uniformly black or light brown, bordered by black; cloacal blotch, if present, brown or black.

**Table 3:** Variations in scale counts of *Boa atlantica* sp. nov.

	Min	Max	mean	SD	n	95%-IC
**Circumorbitals**	13	20	15.9	1.4	122	0.25
**Suboculars**	0	2	0.4	0.6	123	0.11
**Supralabials**	18	25	20.8	1.5	122	0.27
**Intrasupraoculars**	13	21	16.7	1.5	123	0.27
**Infralabials**	20	29	24.5	1.5	123	0.27
**Gulars**	12	22	16.2	1.6	121	0.29
**Preventrals**	0	6	1.3	1.1	120	0.20
**Ventrals**	225	248	235.2	4.1	115	0.75
**Subcaudals**	31	59	51.5	4	120	0.72
**Anterior dorsal scale rows**	48	70	58.7	4	119	0.72
**Midbody dorsal scale rows**	67	93	79.5	5	120	0.89
**Posterior dorsal scale rows**	38	58	47.9	3.4	124	0.60
**Tail dorsal scale rows**	17	26	20.9	2	122	0.35

95%-CI = 95% confidence interval; max = maximum; min = minimum; n = number of samples; and SD = standard deviation.

#### Sexual secondary dimorphism

Despite the size of the lateral cloacal spurs, which is larger in males than in females, we found evidence of sexual dimorphism in *Boa atlantica* sp. nov., as confirmed by the MANOVA (F = 1038; p<0.03). The variables in which we found sexual differences were: number of subcaudal rows (F_1,28_ = 6.49; p<0.02), number of tail spots (F_1,28_ = 6.19; p<0.02), eye-mouth distance (F_1,28_ = 4.88; p<0.04), head width (F_1,2_ = 4.24; p<0.05). Comparing the central tendency measures and 95% confidence intervals (Table 4), females have broader heads and have larger eye-mouth distance than males, and males have more subcaudals and more tail spots than females. The largest male is from São Fidelis, state of Rio de Janeiro (IBSP 4620) 2114 mm SVL, 290 mm TL; largest female from Floresta da Tijuca, Rio de Janeiro, state of Rio de Janeiro (MNRJ 26886) 1.808 mm SVL, 245 mm TL.

**Table 4:** Basic descriptive statistics for the sexual dimorphic characters of *Boa atlantica*. max = maximum; min = minimum; n = number of samples; SD = standard deviation.

	Sex	Min	max	Mean	SD	n
Head width (mm)	M	22.9	57.6	35.3	8.1	21
	F	29.8	52.7	39.7	6.6	26
Distance Eye-Mouth (mm)	M	3.5	7.4	5.3	1.3	20
	F	4.3	8.8	6.5	1.4	26
Number of subcaudal rows	M	47	58	52.9	2.8	26
	F	31	56	48.0	5.0	25
Number of tail spots	M	4	6	5.0	0.6	26
	F	2	6	4.3	0.8	25

Abbreviations: F = female; M = male.

#### Comparison with South American mainland congeners

**Fig 6:**
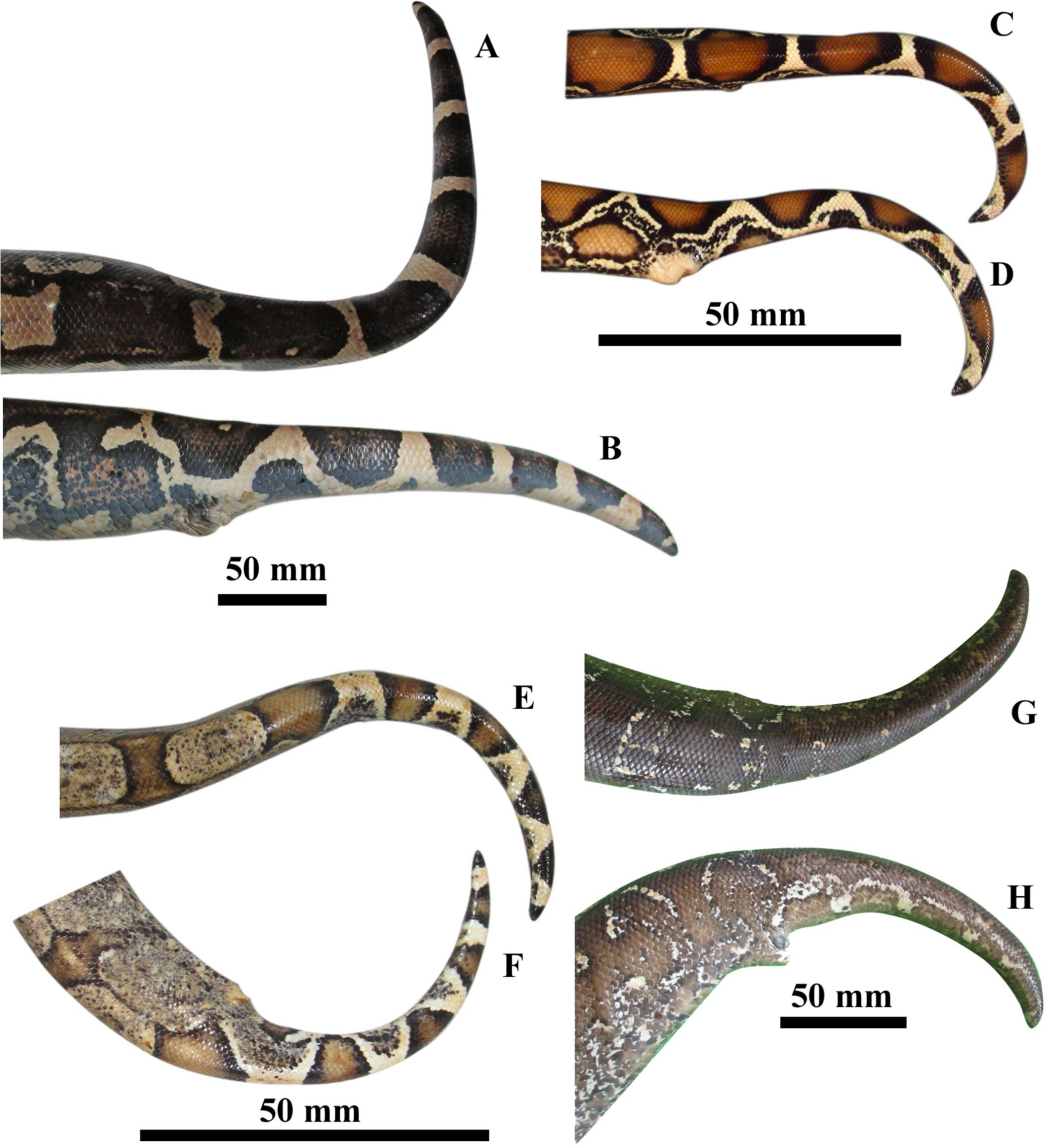
Pre-cloacal region and tails of mainland South-American boas. *B*. *atlantica* sp. nov. (MNRJ 27243): a) superior view, b) lateral view; *B*. *constrictor* (USNM 566533): c) superior view, d) lateral view; *B*. *amarali* (UFG 134): e) superior view, f) lateral view; *B*. *occidentalis* (FML28405): g) superior view, h) lateral view.

*Boa atlantica* **sp. nov.** differs from *B*. *constrictor amarali* (in parenthesis) by having belly cream with tones of orange, brown and black, scattered of black dots and large groups of black spots (vs. heavily pigmented with black and brown); posterior dorsal spots not blotched (vs. blotched); and tail interspots absent (vs. fuzzy) (Fig 6). *Boa atlantica* **sp. nov.** differs from *B*. *c*. *constrictor* (in parenthesis) by having posterior dorsal saddle spots shape similar to the anterior spots (vs. last dorsal saddles comprising large red polygons different from the anterior in *B*. *c*. *constrictor*); last lateral ocelli dark brown, black or dark red (vs. blood-red in *B*. *c*. *constrictor*) (Fig 6); tail spots black (vs. blood-red in *B*. *c*. *constrictor*) (Fig 6). *Boa atlantica* **sp. nov.** differs from *B*. *nebulosa* (in parenthesis) by having 228–243 ventrals (vs. 256–269 in *B*. *nebulosa*), 69–90 midbody dorsal scale rows (vs. 59–69 in *B*. *nebulosa*), subocular stripe present (vs. absent in *B*. *nebulosa*), posterior dorsal spots and saddles do not change shape (vs. posterior saddles fuse with lateral ocelli forming brown bands in *B*. *nebulosa*), and belly with shades of brown and black with and randomly distributed black dots, and larger spots concentrated on the paraventral region (vs. belly heavily pigmented with black, brown dots and shades in *B*. *nebulosa*). *Boa atlantica* **sp. nov.** differs from *B*. *orophias* (in parenthesis) by having 228–243 ventrals (vs. 262–280 in *B*. *orophias*), 44–59 subcaudals (vs. 63–69 in *B*. *orophias*), 17–23 dorsal spots (vs. 25–30 in *B*. *orophias*), 3–6 tail spots (vs. 6–9 in *B*. *orophias*), longitudinal head stripe usually continuous with regular borders and no lateral projections (vs. longitudinal head stripe usually fragmented, with carved borders and none, two or multiple lateral projections in *Boa orophias*). *Boa atlantica* **sp. nov.** differs from *B*. *c*. *occidentalis* (in parenthesis) by having 17–23 dorsal spots (vs. 23–29 in *B*. *c*. *occidentalis*); general colour light to dark brown (vs. dark brown in *B*. *c*. *occidentalis*); longitudinal head stripe with no lateral projections (vs. two or more projections in *B*. *c*. *occidentalis*); lateral ocelli dark brown, black or faint reddish (vs. cream in *B*. *c*. *occidentalis*); belly cream with tones of orange, brown and black, scattered of black dots and large groups of black spots (vs. heavily variegated with brown, yellow, black and white blotches in *B*. *c*. *occidentalis*); belly progressively darker towards tail (vs. no changes in belly pattern in *B*. *c*. *occidentalis*); Tail spots black (vs. cream in *B*. *c*. *occidentalis*) (Fig 6). Additionally, *B*. *occidentalis* was always recovered as monophyletic on the molecular analyses, although grouped either as the sister group to the South American representatives or as the sister group to the Mexico + Central America Clade, depending on the markers and the dataset used. We refer to S1–S4 Tables for the meristic and morphometric data on *B*. *atlantica*, *B*. *occidentalis*, *B*. *orophias* and *B*. *nebulosa* (respectively).

#### Hemipenial morphology of the sympatric congeners

The hemipenes of the sympatric *Boas* are similar in several aspects: organ bilobed, non-capitate, and non-calyculate; lobes relatively short and sub-cylindrical with rounded apices, similar in size and oriented centrifugally; sulcus spermaticus divides on distal portion of hemipenial body below sulk bifurcation; sulcus spermaticus branches centrifugally oriented running to tip of lobes; margins of sulcus spermaticus bordered by flounces on basal to most of distal portion of lobes; sulcus spermaticus expanded at apices of lobes; hemipenial body subcylindrical; proximal region of hemipenis naked.

Nevertheless, the hemipenes of *Boa atlantica* **sp. nov.** (Fig 5) differ from those of *B*. *c*. *constrictor* and *B*. *c*. *amarali* (in parentheses) by having lobes bifurcated at about 70–74% of the total length (vs. at 54–79% in *B*. *c*. *constrictor* and 65–76% in *B*. *c*. *amarali*), lobes covered by 4–6 transversal flounces on its basal portion (vs. 6–8 flounces in *B*. *c*. *amarali*), lobes naked from median to apical region (vs. with shallow flounces in *B*. *c*. *constrictor* and *B*. *c*. *amarali*); distal region of hemipenial body with 5–7 transversal flounces connected to the sulcus spermaticus (vs. 4–6 in *B*. *c*. *constrictor* and *B*. *c*. *amarali*).

#### Distribution

**Fig 7:**
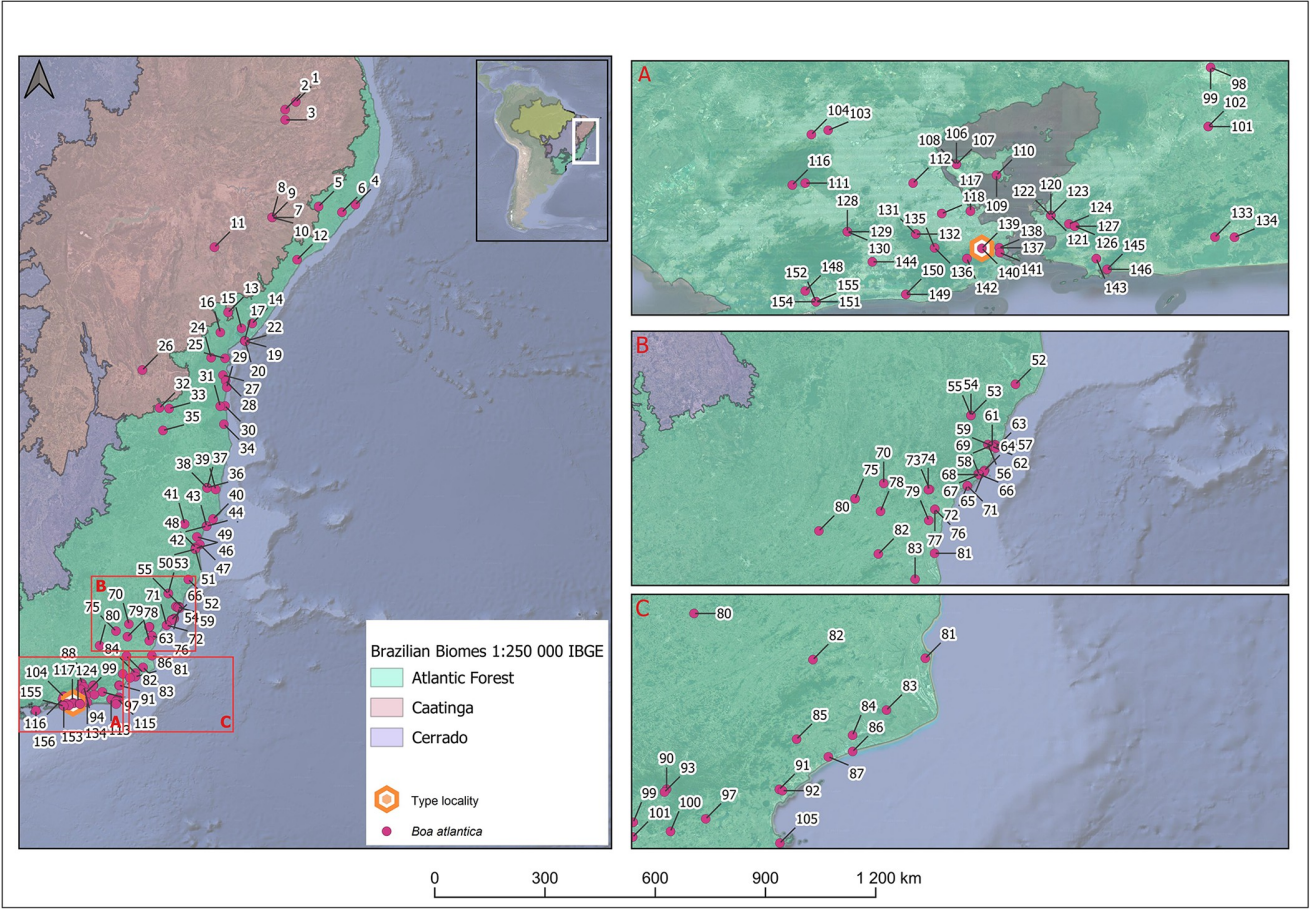
Distribution of *Boa atlantica* sp. nov. based on the examined vouchers and tissue samples. A) Detailment in Rio de Janeiro City, including type locality. B) records in northern Rio de Janeiro and Espírito Santo States, C) records in Rio de Janeiro State. See also S4 Table.

*Boa atlantica* **sp. nov.** is found along the coastal Atlantic Forest along eastern Brazil from Caicó (6°27’23.2"S 37°06’05.8"W; Fig 7 and S5 Table, point 1) in Rio Grande do Norte State to Ilha Grande (23°08’48.4"S, 44°13’40.3"W; Fig 7 and S5 Table, point 156) in Rio de Janeiro State, which is also de westernmost point in the southern distribution. The westernmost point in the northern distribution is Ituaçú (13°48’26.7"S 41°18’39.5"W; Fig 7 and S5 Table point 26) in Bahia State.

In this heterogeneous environment, *B*. *atlantica* **sp. nov.** is found from the sea level up to 906 m a.s.l. (Fig 7).

## Discussion

### Phylogeny and systematics of the genus *Boa*

The genus *Boa* has been rather taxonomically stable since its original description in 1758 [3,28,29]. Otherwise, because there was no study focused on the taxonomy for the entire genus, at least through the application of rigorous (statistical or phylogenetic) methodology, several lineages have been traditionally recognized as subspecies [5,25,29,74]. Bonny [3] presented a review of the names in the genus, even though his study does not present a replicable methodology, examination of available type material, nor is based on large number of specimens to allow statistical confidence. The renewed systematic interest in the genus launched with the study of Hynkovà *et al*. [30]. Since then, several studies have been performed with molecular data, in order to understand the phylogenetic relationships within the genus *Boa* [11,12,31,32].

Despite the rearrangements proposed in previous studies, Reynolds & Henderson [32] published a checklist of the Family Boidae, in which they recognized five species for the genus, as such: (i) *Boa constrictor* (with four subspecies; *B*. *c*. *constrictor*, *B*. *c*. *longicauda*, *B*. *c*. *occidentalis* and *B*. *c*. *ortonii*), (ii) *Boa imperator* (with two subspecies; *B*. *i*. *imperator* and *B*. *i*. *sabogae*), (iii) *B*. *nebulosa*, (iv) *B*. *orophias* and (v) *B*. *sigma*. In this framework, only one species corresponds to the South American cis-Andean taxa: *Boa constrictor*, even though two of its subspecies do not belong to the “*B*. *constrictor* Clade” (*sensu* Hynkovà *et al*. [30]) nor they are cis-Andean (e.g., the trans-Andean *B*. *c*. *longicauda* and *B*. *c*. *ortonii—*here considered as *B*. *i*. *longicauda* and *B*. *i*. *ortonii*).

Actually, the species delimitation into the entire genus, mainly along the cis-Andean populations (= *Boa constrictor* Clade *sensu* Hynkovà *et al*. [30]), has never flourished due for a series of reasons taken alone or in combination, as such: (i) large animals with many samples available in disparate and distant scientific collections allied to taxa with wide geographic distribution, implying a great logistic investment and personal commitment for the examination of representative samples; (ii) recently diverged taxa, usually presenting similar external morphology, at least under first inspection, without a more detailed approach establishing geographical boundaries for each taxa; (iii) very scarce or limited samples (morphological or molecular) for several taxa with very restricted distribution like islands (e.g., *B*. *nebulosa*, *B*. *orophias*, *B*. *i*. *sabogae*, and *B*. *sigma*) or remote trans-Andean regions (e.g., *B*. *i*. *longicaudata* and *B*. *i*. *ortonii*); (iv) most species and subspecies are listed as CITES [51], consequently there are difficulties for new collections and loaning the available material; (v) superficial analyses and/or disregarding colour pattern, meristic and morphometric characters as a valid source of taxonomic evidence; (vi) reduced genetic divergence among some taxa and several short branches along the phylogenetic structure along the genus; and (vii) difficulty in amplifying some genes for new samples using primers available in the literature.

Molecular phylogenies obtained herein always recovered *Boa c*. *occidentalis* and *Boa atlantica* **sp. nov.** with high supports, independently on the markers and the dataset used. *B*. *nebulosa* and *B*. *orophias* also were recovered in a clade with relatively high support. Our decision in describe *Boa atlantica* **sp. nov.** in the specific category is broadly founded in conceptual framework (e.g., [75]). For instance, the use of subspecific rank within a historical context of phylogenetic inference is severely restricted, demanding the same kind of evidence (i.e., unambiguous diagnostic features) needed to recognize a species [76]. In addition, as recently pointed out by Burbrink and colleagues, ontologically, the rank of subspecies is either identical to that of species or undefined terminal in the context of evolutionary lineages representing spa-tiotemporally defined individuals [77]. In following such ideas, we realized that *Boa c*. *occidentalis* also displaying robust morphological diagnosability in combination with solid molecular support and divergence (*sensu* Vences *et al*., [78]. Genetic distances of 3%, 4% and more are robust supports for diagnosis of species in snakes (Figs 2 and S3). For instance, *Boa c*. *occidentalis* has always been retrived with high support values in a basal position within the *Boa constrictor* Clade [30,35,79], and presents several diagnostic features regarding its congeners [3,80]. Therefore, in order to maintain the reciprocal monophyly between the previously recognized taxa (Fig 2), we propose here the elevation of *Boa occidentalis* to full specific rank. On the other hand, a comprehensive phylogeography study is required in order to make a robust taxonomic decision about the validity (or not) of remained subspecies of the *Boa constrictor* Clade (i.e., *B*. *c*. *amarali* and *B*. *c*. *melanogaster*) and we refrain here to take any hasty decision in this respect.

### The new boa from the Atlantic Forest

*Boa atlantica* **sp. nov.** occurs in the most populous area in Brazil, where the Portuguese colonization began in the XVI Century through an occupancy plan called ‘Capitanias Hereditárias’, which gave rise—with many boundary shifts across time—to the current political division of the country. The herpetofauna is well-known along the Brazilian coast, at least if compared to most inland areas, partially because this region concentrates the largest capitals of the country, including most of the research centres, scientists and natural history collections. On other hand, it is remarkable that early naturalist travelling to Brazil (e.g., [81]) or even nowadays (e.g., [73]), reported on such coastal population of boas, without noting its morphological distinction with respect to Amazon (i.e., *B*. *c*. *constrictor*) or Cerrado (i.e., *B*. *c*. *amarali*) populations.

Nevertheless, as stated before, there has never been a comprehensive taxonomic review of the genus to establish specific boundaries accurately, mainly considering cis-Andean taxa. In addition, several studies did not mention explicitly the presence of the genus *Boa* in the Atlantic Forest [68,69,72,82]. By contrast, the few mentions of boas in the Atlantic Forest regard it as *Boa constrictor* without consistent application of trinomial nomenclature comprising subspecific ranks [3,6,9,70,71,73,74,83–85].

*Boa atlantica* **sp. nov.** is very common in its distribution range, being found in lowland primary and secondary rainforests [71,73], and even in large and populous cities with very anthropized environment, such as Rio de Janeiro (see Figs 7 and S2 File). *Boa atlantica* **sp. nov.** may occur in sympatry with nominal species *B*. *c*. *constrictor* in its northernmost area of distribution in Passo do Camaragibe, state of Alagoas, Brazil, since the area is too close to the Caatinga. This can be explained considering the Brazilian Atlantic Forest has once been enormous, occupying a vast territory especially in the Atlantic coast [86–89]. Since the discovery and colonization of Brazil, this biome has been widely destroyed (due to farming, large cities, wood-market, and so on), with less than 12% of the forest currently remaining [87–90].

This situation is more overwhelming in the northeastern region of Brazil, where the remaining forest is patched or transformed into monocultures or pastures [86–89]. The destruction of the forest has had a rough and direct impact on the fauna distribution, since it is dependent on the local resources [91]. Likewise, the forest fragmentation and advancement of the Caatinga and Cerrado, respectively, into Northeast and Southeast Brazilian regions might explain the areas where *B*. *atlantica* **sp. nov.** has contact with *B*. *c*. *amarali* (Espírito Santo and possibly western Bahia States), and *B*. *c*. *constrictor* (Alagoas and western Bahia State, Brazil). The southernmost record is for Ilha Grande (Rio de Janeiro State, Brazil). In this case, there is a large gap between the records of *B*. *c*. *amarali* from São Paulo City and *Boa atlantica* **sp. nov.** in southern Rio de Janeiro State, since *B*. *c*. *amarali* does not occur in the coast of the state of São Paulo, Brazil.

The present study formally describes a new species from a population that has been known by scientists in the past 200 years. In a certain way, we can assume that *Boa atlantica* **sp. nov.** has been widely ignored by majority of taxonomist, since it has always been regarded as distinct from subspecies *Boa c*. *constrictor* or *B*. *c*. *amarali*, being usually referred only to as *Boa constrictor* without a consistent trinomial usage. Here we reinforce the importance of paying attention to the most common species, which, if not studied, may hide cryptic diversity. Just like our example here, Feinberg *et al*. [92] described a new species of *Rana* (*R*. *kauffeldi*) inside New York City. Although such discoveries may be unexpected in densely populated urban parts of the world, detailed comparative studies demonstrated that new species can still be found periodically even in well-studied places rarely associated with undocumented biodiversity [92]. We also stress the importance of protecting such species, since it is endemic to the Brazilian Coastal Atlantic Forest, a largely impacted and threatened ecoregion. Therefore, this new species was already threatened by habitat loss even and other threats even before it was formally described by science.

### Is there cryptic diversity in the genus *Boa*?

Currently, cryptic species could represent a substantial fraction of biodiversity and, consequently, such phenomena are calling attention and being discovered at a speeding pace [92,93]. Nevertheless, it is known that the diversity of cryptic species is still underestimated [94] or even misunderstood [95]. For instance, at first the case of *Boa* spp. seems to fit well to general processes associated with concentration of cryptic diversity, such as: recent divergence in combination with a certain level of morphological stasis. Notwithstanding, many of currently recognized species present apparently fixed diagnostic features mostly based on external morphology (e.g., colour tones and patterns). As a rule, such character systems have been disregarded if compared to other sources of phenotypic characters (e.g., male geniatlia and osteological features) in several groups of snakes (see [96]). However, for all other extant boid the colour patterns have proven to hold interesting phylogenetic signals [97–100]. By following definitions of cryptic species [95], we believe that most taxa currently recognized as full species along the *Boa constrictor* Clade (i.e., *Boa atlantica* **sp. nov.**, *Boa nebulosa*, and *Boa orophias*) are not cryptic at all, since, even if tenuous, they present unambiguous morphological diagnosis from each other congener, including the nominal form. In fact, our results suggest that a plethora of phenotypic data examined here are broadly congruent with the molecular evidence, not the contrary, in an integrative taxonomy approach, despite recent diversification revealed by short branches recovered within the group. Therefore, we highlight the importance of studying several sources of phenotypic evidence, even if there is a suggestion of high levels of polymorphism. However, as pointed out by Lee & Palci [101] if morphology is to be employed to its full potential, biologists need to start scrutinising phenotypes in a more objective fashion, models of phenotypic evolution need to be improved, and approaches for analysing phenotypic traits together with genomic data need to be refined.

## Supporting information

S1 Fig Phylogenetic tree inferred by using Maximum Likelihood on CIPRES (*GTR+IO+G* model), based on 744 partial sequences of cyt-*b*. The matrix is composed of 73 samples from Mexico, Central and South America (including the outgroup—*Charina bottae*, *Corallus hortulana*, *Epicrates cenchria*, and *Eunectes murinus*). The bootstrap value is shown above the branches. Group colours: black: outgroup; blue: *B*. *occidentalis*; green: *B*. *atlantica* sp. nov.; grey: *B*. *imperator*; orange: *B*. *constrictor* + *B*. *amarali*; pink = *B*. *orophias + B*. *nebulosa*.
(TIF)

S2 Fig Phylogenetic trees. Phylogenetic tree inferred by using Bayesian Inference on CIPRES, based on (A) 73 samples from Mexico, Central and South 744 partial sequences of cyt-b, and (B) the four concatenated markers (cyt-*b*, ND4, NTF3, and ODC) resulting in 2305 positions in the final dataset. Outgroup is composed of *Charina bottae*, *Corallus hortulana*, *Epicrates cenchria*, and *Eunectes murinus* with cyt*b* only. Posterior Probabilities are indicated on the branches.
(ZIP)

S3 Fig Estimates of evolutionary divergence between sequences based on cyt-*b* partial mitochondrial gene. (TIF)

S1 Table Meristic and morphometric data of the examined specimens of *Boa atlantica* sp nov. (PDF)

S2 Table Meristic and morphometric data of the examined specimens of *Boa occidentalis*. (PDF)

S3 Table Meristic and morphometric data of the examined specimens of *Boa orophias*. (PDF)

S4 Table Meristic and morphometric data of the examined specimens of *Boa nebulosa*. (PDF)

S5 Table Pinpoint of vouchers and samples used to compose the distribution map of *Boa atlantica* sp nov (Fig 6). Holotype is marked in bold. (PDF)

S1 File Polymerase chain reaction (PCR) protocol for four genes amplification. (PDF)

S2 File Specimens examined for morphology. (PDF)
