# Correction: The good, the bad and the boa: An unexpected new species of a true boa revealed by morphological and molecular evidence

**DOI:** 10.1371/journal.pone.0359138

**Published:** 2026-09-25

**Authors:** Rodrigo Castellari Gonzalez, Lorena Corina Bezerra de Lima, Paulo Passos, Maria José J. Silva

The data underlying some of the results in this article [1] are missing from the list of Supporting Information. The authors have provided the data as Supporting Information files S6 and S7 Tables. With this correction, all relevant data are now provided.

The Data Availability statement is incorrect. The correct Data Availability statement is: All relevant data are within the paper and its Supporting Information files. The DNA sequences can be obtained from the National Center for Biotechnology Information GenBank database using the accession numbers in S7 Table.

## Supporting information

S6 Table Qualitative and quantitative variability on the hemipenial morphology for Brazilian species and subspecies of the genus *Boa.* (XLSX)

S7 Table GenBank accession numbers for the Cyt*b*, ND4, NTF3, and ODC sequences. (XLSX)
