# Supplementary figures and images for: The good, the bad and the boa: An unexpected new species of a true boa revealed by morphological and molecular evidence
Source: PLoS One. 2024 Apr 17;19(4):e0298159. doi: 10.1371/journal.pone.0298159 (PMC11023597; doi:10.1371/journal.pone.0298159)

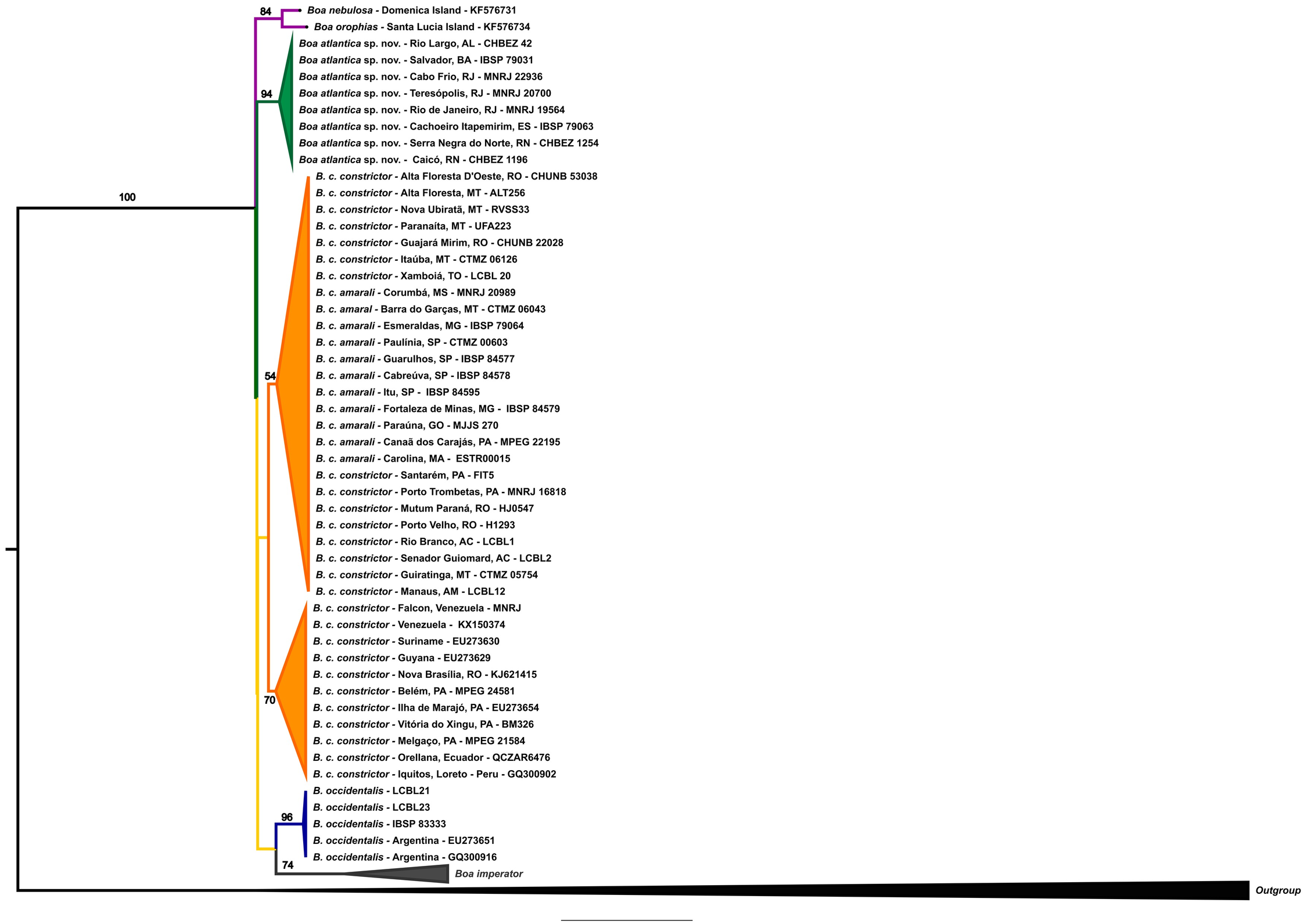

Supplement: S1 Fig — The matrix is composed of 73 samples from Mexico, Central and South America (including the outgroup—Charina bottae, Corallus hortulana, Epicrates cenchria, and Eunectes murinus). The bootstrap value is shown above the branches. Group colours: black: outgroup; blue: B. occidentalis; green: B. atlantica sp. nov.; grey: B. imperator; orange: B. constrictor + B. amarali; pink = B. orophias + B. nebulosa. (TIF) [file pone.0298159.s001.tif]

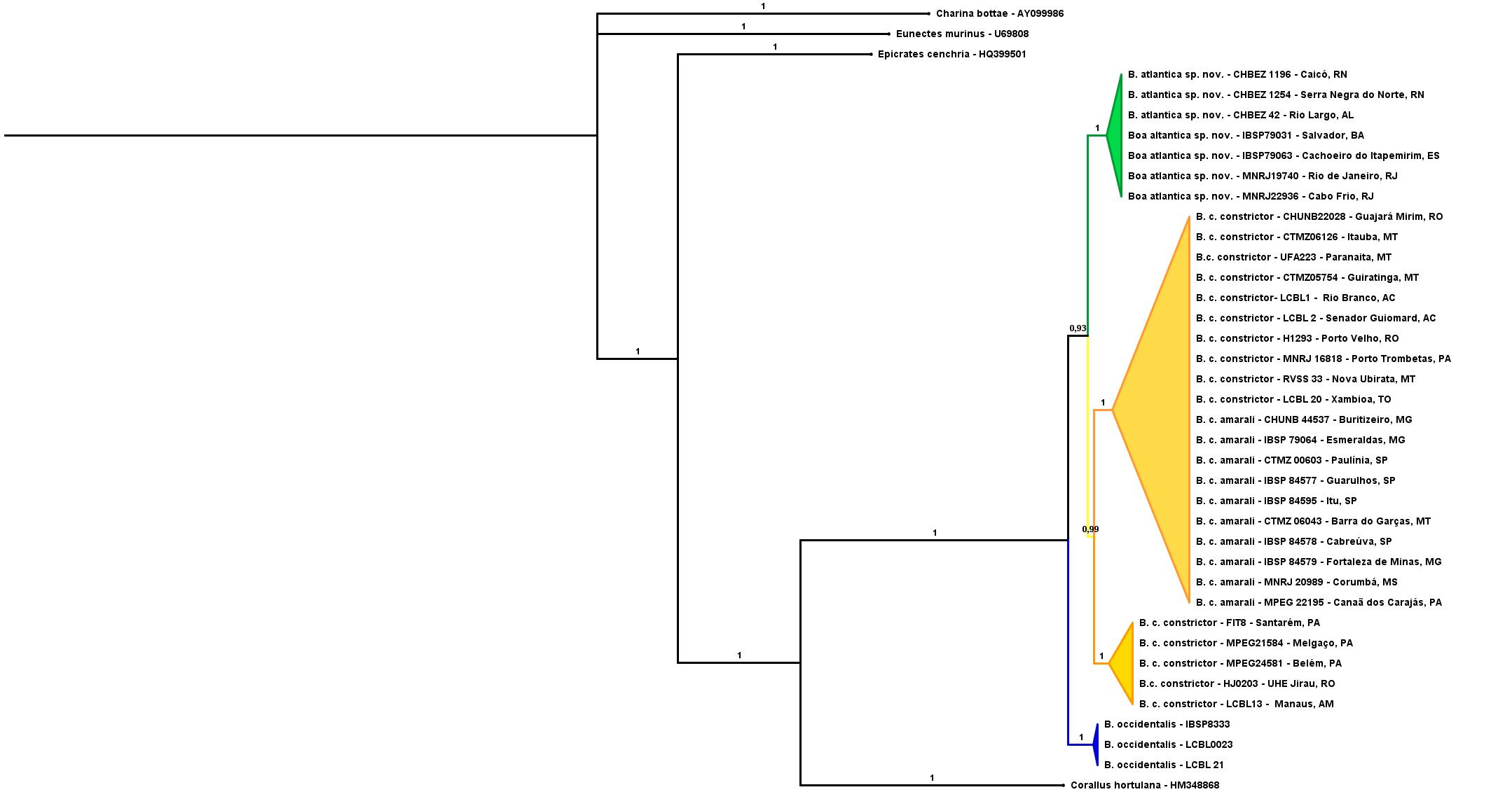

Supplement: S2 Fig — Phylogenetic tree inferred by using Bayesian Inference on CIPRES, based on (A) 73 samples from Mexico, Central and South 744 partial sequences of cyt-b, and (B) the four concatenated markers (cyt-b, ND4, NTF3, and ODC) resulting in 2305 positions in the final dataset. Outgroup is composed of Charina bottae, Corallus hortulana, Epicrates cenchria, and Eunectes murinus with cytb only. Posterior Probabilities are indicated on the branches. (ZIP) [file pone.0298159.s002.zip › S2a_Fig.tif]

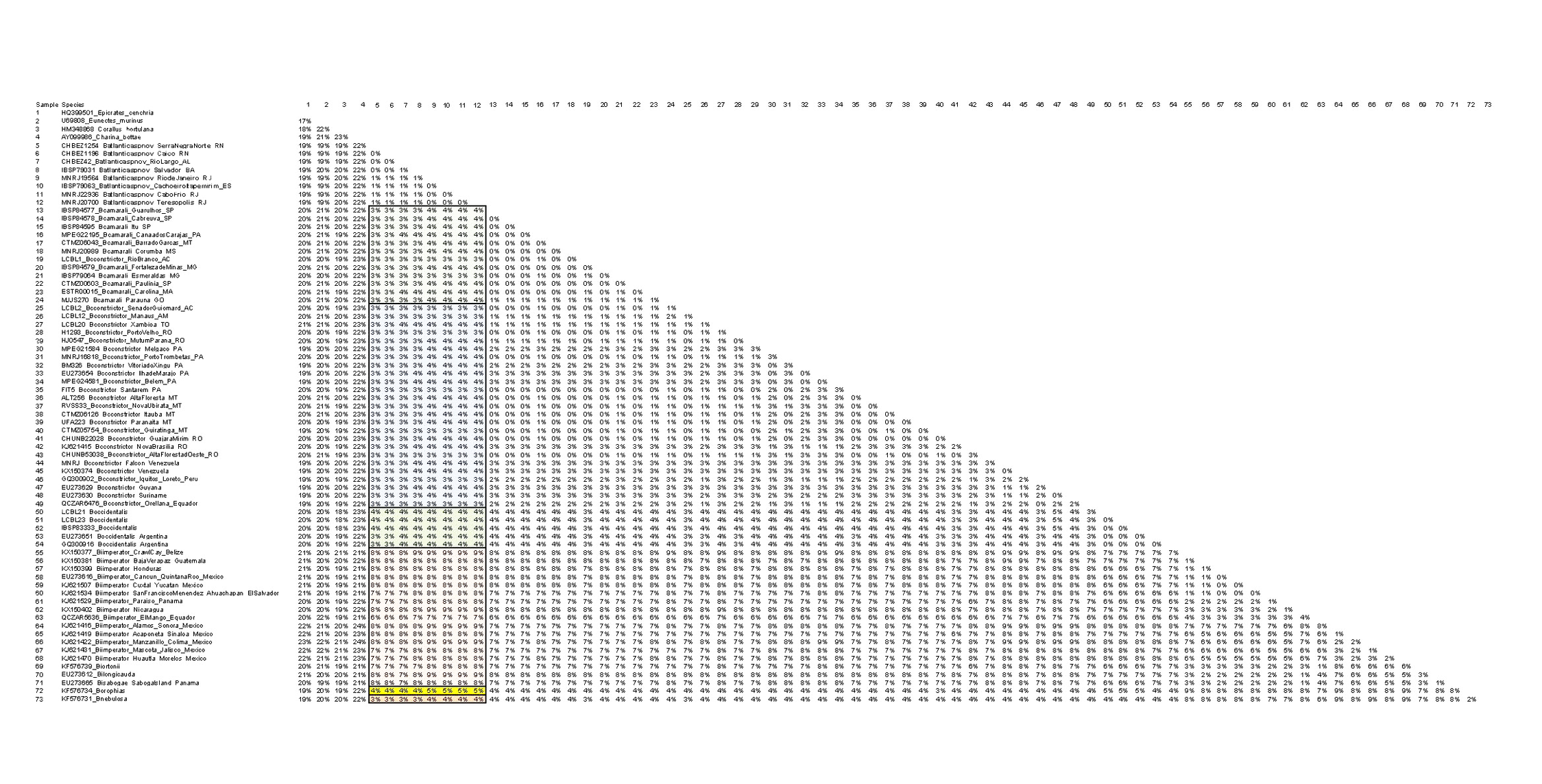

Supplement: S3 Fig — (TIF) [file pone.0298159.s003.tif]
