# Supplementary material for: The good, the bad and the boa: An unexpected new species of a true boa revealed by morphological and molecular evidence
Source: PLoS One. 2024 Apr 17;19(4):e0298159. doi: 10.1371/journal.pone.0298159 (PMC11023597; doi:10.1371/journal.pone.0298159)
Supplement: S1 Table — (PDF) [file pone.0298159.s004.pdf]

S6 Meristic and morphometric data of the examined specimens of *Boa atlantica* sp nov.

AD = anterior dorsal rows; CIRC = number of circumorbital scales; EMD = eye-mouth distance; F = female; GUL = Gulars; HH = head height; HL = head length; HW = head width; IL = Infralabials; INTR = intrasupraocular scales; M = male; MD = midbody dorsal rows; NS = number of saddles; NTS = number of tail spots; PD = posterior dorsals rows; PV = pre-ventrals; SC = subcaudals; SL = supralabial scales; SUB = subocular scales; SVL = snout-vent length; TD = tail dorsals; TL = tail length; V = ventral.

Acronym	Number	SE-	SVL	TL	HW	HL	HH	EMD	CIRC	SUB	SL	INTR	IL	GUL	PV	V	SC	AD	MD	PD	TD	NS	NTS
CHUFV	2267	M	1,819.00	269.00	42.40	55.90	21.10	5.70	14	0	21	16	24	19	3	232	53	54	84	42	20	22	6
CZGB	705	F	992.00	112.00	32.79	53.33	15.59	5.35	13	0	21	19	25	18	2	230	48	62	82	50	21	21	4
CZGB	1291	M	1,915.00	272.00	42.99	69.39	22.42	7.31	17	2	20	17	24	16	2	239	57	65	83	48	23	18	5
CZGB	1315	M	632.00	92.00	21.37	37.92	12.85	3.14	17	1	23	16	29	17	2	237	46	63	82	53	20	18	5
CZGB	2712	F	1,451.00	142.00	30.03	62.82	20.37	6.18	17	1	21	16	26	17	2	245	46	65	90	50	21	20	4
CZGB	2713	F	1,765.00	164.00	41.57	67.17	24.56	6.45	17	1	22	16	26	17	0	235	47	63	93	54	22	21	4
CZGB	2820	M	1,297.00	219.00	-	-	-	-	-	-	-	19	-	-	-	-	51	68	77	51	22	22	5
CZGB	3051	M	622.00	94.00	19.78	33.89	11.45	3.19	16	2	23	16	25	15	2	234	54	61	76	46	21	18	4
CZGB	4862	F	826.00	114.00	24.57	45.71	15.25	4.60	16	2	22	15	24	17	1	239	51	64	88	48	25	19	4
CZGB	7949	M	461.00	66.00	17.40	31.18	11.16	2.58	17	1	22	18	27	17	2	236	50	70	85	54	25	21	4
CZGB	8389	F	549.00	71.00	20.77	36.57	12.38	2.94	17	0	22	18	26	16	2	234	52	62	78	56	26	24	4
CZGB	8514	M	2,003.00	249.00	-	-	-	-	19	0	23	18	25	18	1	231	56	58	82	48	22	18	5
IBSP	4620	M	2,114.00	290.00	-	-	-	-	-	-	-	-	-	-	0	235	50	-	-	44	22	-	5
IBSP	47431	M	-	-	33.40	61.40	26.30	7.10	16	1	19	17	24	14	0	225	-	-	-	45	-	-	-
IBSP	52164	-	-	-	16.90	28.90	9.80	3.20	13	0	21	17	24	16	-	-	-	59	-	-	-	-	-
IBSP	52165	-	-	-	-	-	-	-	-	-	-	-	-	-	-	-	-	-	-	-	-	-	-
IBSP	79033	F	1,127.00	119.00	29.80	48.90	14.90	4.40	16	0	21	20	24	15	1	234	50	58	86	54	20	19	4
IBSP	79063	F	714.00	85.00	19.80	35.80	13.90	2.80	16	0	19	16	23	16	1	233	49	58	83	47	20	17	4

IBSP	79086	F	871.00	94.00	23.50	40.00	12.70	3.45	19	0	22	18	26	17	6	239	48	61	81	54	22	19	4
IBSP	79163	F	1,676.00	161.00	40.30	65.60	22.00	6.30	16	0	21	20	20	16	1	239	47	63	85	55	21	23	4
IBSP	79481	M	439.00	64.00	13.40	25.70	9.10	2.30	16	0	19	16	25	12	0	231	51	55	75	45	19	17	6
IVB	2953	M	1,012.00	132.00	-	-	-	-	17	1	23	19	24	16	2	241	58	63	84	50	23	20	5
IVB	3379	F	482	62	16.04	27.64	10.43	2.11	17	1	19	15	22	15	2	237	50	53	76	43	19	23	4
IVB	3381	M	650	79	21.33	34.02	10.9	2.34	13	1	22	16	24	15	2	239	58	58	70	48	22	22	5
IVB	3422	F	1450	174	43.24	74.31	23.83	7.95	17	1	20	16	24	16	1	240	38	60	81	45	23	18	5
MBML	58	F	468.00	68.00	16.39	30.29	10.35	2.45	14	0	20	17	25	17	0	232	-	59	73	47	23	17	5
MBML	1932	-	-	-	33.83	57.01	18.69	4.24	15	0	22	19	25	16	3	225	-	64	80	58	-	18	-
MBML	1933	M	1,706.00	258.00	57.60	87.70	24.40	6.80	17	0	22	16	24	16	2	235	55	59	74	50	26	20	5
MBML	1934	M	1,407.50	135.00	-	-	-	-	14	0	23	18	26	15	1	233	48	62	79	49	21	20	4
MBML	1935	F	940.00	129.00	29.91	51.10	15.98	5.11	16	0	22	18	26	16	0	228	51	63	81	49	23	21	5
MBML	1936	F	2,201.00	200.00	45.00	74.15	29.50	7.80	17	2	21	19	25	17	3	235	51	60	82	47	23	24	5
MBML	1937	F	1,072.00	112.00	36.84	50.19	-	4.38	13	0	20	18	24	18	0	236	49	55	72	48	22	21	5
MBML	2001	F	481.00	56.00	16.00	28.80	9.30	2.20	15	1	20	17	25	17	1	234	47	53	73	46	18	19	4
MBML	2013	F	805.00	91.00	23.00	41.30	12.90	3.40	15	0	20	16	24	19	1	239	51	59	76	52	22	23	5
MBML	2097	M	1,005.00	109.00	22.90	45.10	-	3.90	16	0	19	16	24	19	1	231	47	54	71	46	20	22	5
MBML	2178	F	481.00	65.00	15.20	27.20	9.70	2.50	15	1	21	17	25	16	0	235	47	50	68	45	22	16	5
MBML	2179	F	1,320.00	145.00	30.10	66.40	21.00	6.60	17	0	20	15	25	17	2	226	48	58	82	43	20	20	4
MBML	2180	F	578.00	80.00	17.80	30.80	11.00	1.70	16	1	22	20	26	16	0	238	58	59	83	45	22	15	5
MBML	2183	M	1,109.00	140.00	26.40	49.70	16.70	4.20	15	1	21	16	25	16	1	237	53	55	78	46	21	20	5
MBML	2217	M	444.00	61.00	14.30	26.10	9.50	-	-	-	-	-	-	-	0	-	-	56	75	48	-	23	5
MBML	2218	F	431.00	68.00	17.90	27.60	8.90	3.70	17	1	20	16	25	16	1	237	50	61	80	48	21	17	6
MBML	2220	F	431.00	58.00	15.60	25.20	9.20	2.40	16	0	20	16	25	17	-	-	48	58	86	54	23	20	5
MBML	2257	F	845.00	101.00	25.00	42.60	14.10	3.60	15	0	22	18	26	17	1	229	49	62	74	52	23	22	6
MBML	2258	M	1,739.00	229.00	39.40	65.50	20.50	4.60	15	0	19	16	23	16	1	233	52	52	75	46	20	22	5
MBML	2315	F	604.00	74.00	19.40	32.50	10.10	2.80	16	0	20	16	27	20	1	232	48	62	82	49	20	-	5
MBML	2853	M	1,832.00	236.00	37.60	60.80	-	-	16	1	21	17	26	16	1	236	52	59	78	49	21	23	5

MBML	2855	F	2,215.00	-	43.00	77.10	27.60	8.20	15	1	24	17	27	15	2	236	-	55	80	49	23	22	-
MCN (RS)	5708	M	2,066.00	262.00	45.50	70.10	22.70	5.80	18	0	22	16	25	15	0	239	54	60	89	48	24	21	5
MCNR (puc-mg)	4828	M	1,352.00	195.00	-	-	-	-	17	1	18	15	27	17	5	229	52	55	79	40	19	22	6
MCP (RS)	2544	F	1,404.00	155.00	38.60	61.30	18.20	5.70	17	0	19	16	23	16	1	237	51	57	81	49	18	20	5
MCP (RS)	2558	M	1,344.00	203.00	33.80	58.30	17.80	5.90	14	0	20	16	23	17	1	235	55	57	80	47	21	18	5
MCP (RS)	2948	M	1,228.00	187.00	28.40	55.40	17.70	4.00	15	0	21	17	24	15	1	237	54	55	75	46	20	20	5
MCP (RS)	3021	M	1,041.00	131.00	29.80	48.20	15.90	3.90	16	0	19	17	25	16	0	236	55	59	84	48	20	22	5
MCP (RS)	13737	M	1,048.00	118.00	27.30	42.20	15.90	3.50	16	0	23	16	24	17	0	-	56	59	82	50	23	22	6
MHNCI	1454	F	534.00	68.00	10.70	29.70	11.10	2.30	13	0	18	15	22	14	2	231	51	52	80	45	18	19	4
MHNCI	1922	M	604.00	78.00	16.50	31.60	11.50	2.20	15	0	18	17	23	14	0	234	54	55	77	38	19	19	4
MHNCI	3097	F	658.00	86.00	20.30	33.70	11.50	3.00	17	0	20	15	22	18	1	236	53	60	89	48	23	22	4
MNRJ	400	F	1,857.00	149.00	39.20	70.10	24.90	7.30	15	0	21	14	24	18	1	-	43	56	80	45	17	22	4
MNRJ	3940	F	1,099.00	110.00	30.30	46.70	15.80	4.40	15	1	25	15	28	17	1	236	52	55	84	49	22	19	4
MNRJ	6361	F	1,754.00	168.00	39.70	69.50	21.90	9.70	16	1	23	16	25	18	1	238	53	57	75	47	20	22	5
MNRJ	6362	M	959.00	122.00	26.20	44.10	13.50	3.70	17	1	21	17	23	18	2	236	58	65	86	51	24	23	4
MNRJ	6364	F	1,386.00	161.00	39.60	66.00	22.20	5.50	16	0	19	15	21	15	1	240	51	58	84	47	19	21	5
MNRJ	8340	M	1,105.00	138.00	33.00	52.80	15.80	5.20	18	1	22	17	24	18	2	230	48	64	79	47	19	20	4
MNRJ	9449	F	453.00	60.00	17.35	26.93	9.24	2.44	15	0	22	16	25	17	0	232	50	63	72	45	22	21	5
MNRJ	9565	M	1,488.00	163.00	35.08	55.34	18.89	4.69	17	1	22	19	25	15	2	232	50	60	81	50	24	23	5
MNRJ	10092	M	1,604.00	228.00	34.08	61.10	19.33	5.19	16	1	20	16	24	16	2	234	53	-	-	43	20	18	4
MNRJ	10117	F	2,185.00	192.00	42.20	83.60	26.50	6.60	14	0	21	17	25	16	1	235	47	58	81	45	19	24	4
MNRJ	11205	F	2,164.00	290.00	45.60	81.80	26.90	8.80	16	0	21	18	25	15	3	236	51	58	87	51	21	21	4
MNRJ	12692	F	1,269.00	145.00	40.85	61.78	19.40	4.91	15	1	20	21	26	16	0	226	49	54	83	48	25	16	4
MNRJ	13111	F	485.00	68.00	15.63	27.36	10.65	2.70	16	1	19	17	23	13	2	234	54	60	80	47	20	20	4
MNRJ	13177	F	594.00	87.00	20.18	31.62	10.60	2.79	15	0	22	16	26	15	5	229	56	60	81	45	21	20	5
MNRJ	14200	F	531.00	66.00	15.75	29.20	11.08	2.72	17	1	22	17	25	15	0	243	51	68	87	52	21	21	5
MNRJ	14201	F	563.00	74.00	20.35	29.91	10.51	3.20	16	0	21	17	25	15	2	237	50	61	76	48	21	19	5
MNRJ	14202	F	505.00	74.00	15.79	27.07	10.77	3.04	14	0	21	16	22	15	3	238	56	54	84	48	20	21	5

MNRJ	14238	F	1,898.00	194.00	52.70	77.10	22.50	8.10	15	0	20	15	24	16	1	232	49	53	75	45	21	20	4
MNRJ	14250	F	476.00	70.00	14.40	26.20	11.00	2.60	14	0	21	18	23	14	1	233	56	56	72	54	20	20	5
MNRJ	15198	F	2,818.00	225.00	47.20	85.20	26.40	8.40	15	1	19	17	25	14	1	240	51	54	78	47	19	22	3
MNRJ	15199	M	1,109.00	304.00	31.30	68.10	25.80	6.80	17	0	20	16	25	17	1	230	52	53	78	42	21	21	5
MNRJ	16436	M	613.00	86.00	18.40	33.40	10.90	2.70	16	1	19	18	23	16	0	238	55	59	75	43	18	17	4
MNRJ	16575	-	-	-	-	-	-	-	15	1	22	13	24	14	-	-	45	-	-	-	-	21	5
MNRJ	17353	F	482.00	68.00	13.06	27.20	9.28	2.76	17	0	19	16	24	-	-	-	55	-	-	-	-	21	5
MNRJ	17846	M	484.00	69.00	-	-	-	-	-	-	-	-	-	-	2	234	54	55	76	46	18	18	5
MNRJ	18269	M	1,159.00	209.00	28.20	50.50	16.30	3.90	14	0	18	14	24	15	2	234	53	57	80	41	19	20	4
MNRJ	18294	F	467.00	64.00	11.60	24.80	9.10	2.10	14	0	-	17	23	-	-	-	50	51	71	-	17	20	5
MNRJ	18535	M	475.00	67.00	15.00	27.70	9.70	2.20	16	1	18	16	23	15	1	232	53	58	80	46	21	22	5
MNRJ	18960	F	1,848.00	105.00	46.70	73.70	27.70	7.30	15	1	20	18	23	17	1	235	31	59	82	44	19	21	2
MNRJ	19412	M	529.00	71.00	16.50	28.70	10.30	2.30	15	0	19	16	22	15	3	236	50	57	80	43	20	19	5
MNRJ	19564	M	514.00	72.00	16.60	28.20	9.80	2.60	17	0	19	16	24	15	1	237	53	54	67	47	20	17	4
MNRJ	19594	F	-	192.00	46.00	69.30	26.30	7.70	14	0	20	17	26	20	-	-	48	-	-	-	20	-	4
MNRJ	19740	M	2,000.00	220.00	46.20	72.50	25.70	7.40	14	0	21	15	24	17	1	237	53	58	79	49	22	20	6
MNRJ	22704	F	393.00	73.00	10.00	27.70	9.40	2.10	17	0	19	18	25	15	0	237	58	53	73	50	19	22	5
MNRJ	22705	F	511.00	69.00	18.10	28.90	18.20	2.60	19	0	23	18	25	19	2	-	53	60	78	42	20	17	5
MNRJ	22936	F	637.00	86.00	19.69	32.35	11.17	3.29	16	0	22	16	25	14	0	237	59	57	82	46	20	20	6
MNRJ	22963	F	521.00	72.00	13.80	29.30	9.80	2.30	17	0	20	16	24	13	0	241	57	58	78	45	20	18	5
MNRJ	23144	F	2,058.00	201.00	41.00	76.00	25.20	7.90	17	1	19	14	24	18	3	239	50	57	83	45	20	22	4
MNRJ	23361	F	1,271.00	141.00	36.90	57.20	18.70	5.90	15	1	19	17	24	17	2	233	49	60	82	49	20	15	4
MNRJ	23573	F	2,038.00	200.00	49.60	78.90	28.60	8.40	15	0	21	18	24	15	2	234	53	56	83	51	23	21	5
MNRJ	23879	M	840.00	104.00	25.60	38.10	13.10	3.20	16	0	21	16	26	16	1	237	54	60	74	50	21	18	5
MNRJ	23880	F	484.00	62.00	-	-	-	-	-	-	-	-	-	-	-	-	-	-	-	-	-	-	5
MNRJ	23881	M	655.00	78.00	20.80	35.10	10.80	2.60	15	0	22	19	25	15	0	-	54	-	-	50	22	-	5
MNRJ	23882	M	985.00	118.00	22.90	43.80	15.80	4.00	16	0	20	15	22	15	3	238	55	59	70	46	22	17	5
MNRJ	24860	F	522.00	68.00	16.60	29.10	-	2.40	17	0	21	15	24	14	1	233	49	-	76	49	19	14	4

MNRJ	24903	M	841.00	101.00	27.50	40.80	13.80	3.50	16	0	20	16	25	15	0	235	53	61	78	49	19	17	5
MNRJ	25057	F	351.00	54.00	12.60	23.70	8.30	1.90	17	0	23	18	25	14	1	240	53	56	74	48	18	21	4
MNRJ	25413	F	1,086.00	139.00	30.38	56.00	17.26	4.27	16	0	22	18	24	18	2	230	44	56	77	52	18	20	5
MNRJ	25950	F	945.00	110.00	25.60	39.10	13.48	3.61	16	0	24	16	28	18	2	235	51	60	-	47	20	20	5
MNRJ	25951	M	958.00	118.00	26.60	45.50	14.20	3.10	16	0	20	15	24	15	1	234	52	-	77	45	18	-	5
MNRJ	25952	F	1,156.00	132.00	31.10	51.30	16.40	5.10	15	0	20	16	25	16	1	237	50	58	80	50	19	21	5
MNRJ	25953	F	730.00	92.00	20.10	36.60	13.30	3.20	15	0	19	17	25	15	1	238	55	57	86	50	20	16	4
MNRJ	25954	F	656.00	83.00	-	-	-	-	-	-	-	-	24	16	-	-	53	-	82	49	20	18	4
MNRJ	26213	F	437.00	63.00	15.60	38.20	8.50	1.80	17	0	22	16	25	15	1	235	50	54	71	50	20	16	5
MNRJ	26324	M	890.00	110.00	24.80	41.60	16.10	4.10	-	0	19	-	-	-	2	234	59	54	71	42	19	22	5
MNRJ	26350	F	594.00	85.00	19.40	32.20	11.10	2.70	15	0	20	15	26	17	2	231	51	61	79	48	21	21	5
MNRJ	26802	M	1460	180	38.32	59.97	19.93	5.59	18	0	20	15	22	16	6	231	54	56	79	47	21	20	5
MNRJ	26886	F	1808	245	46.93	87.15	36.08	9.84	13	0	20	15	21	13	3	238	54	52	79	43	19	21	5
MNRJ	27242	M	2616	294	42.5	77.9	27.5	8.2	17	1	22	17	26	18	2	245	57	64	90	50	24	22	4
MNRJ	27243	F	2650	265	56.9	83.3	33.7	10.2	0	18	22	16	26	16	1	255	59	63	87	47	23	21	5
MZUESC	4696	M	778.00	85.00	23.19	37.36	13.78	3.55	18	1	22	19	26	18	0	244	-	64	86	49	-	19	4
MZUESC	6310	M	1,028.00	169.00	34.80	54.90	10.01	4.69	14	0	21	16	25	17	2	243	54	62	84	47	22	22	4
MZUFBA	379	M	555.00	76.00	17.20	31.60	10.40	2.80	20	1	22	18	25	16	2	237	51	58	78	50	22	19	5
MZUFBA	1165	M	528.00	79.00	15.20	31.00	10.10	2.30	17	1	22	20	27	-	0	243	-	62	84	50	24	15	6
MZUFBA	1166	M	921.00	109.00	22.70	45.20	3.90	3.60	17	1	25	19	18	18	0	242	54	67	83	58	24	19	5
MZUFBA	1167	M	579.00	74.00	14.80	30.50	10.80	2.40	18	2	20	19	25	16	1	239	52	68	89	53	23	18	4
MZUFBA	1168	F	935.00	95.00	25.40	43.50	14.20	3.90	14	2	24	20	26	20	2	236	48	71	88	53	23	15	5
MZUFBA	1514	M	1,190.00	175.00	30.60	59.60	18.30	5.80	14	0	22	17	24	19	1	230	55	64	78	48	24	14	6
MZUFBA	1968	M	795.00	99.00	-	-	-	-	14	1	23	16	27	19	2	248	49	67	81	54	18	17	4
MZUFBA	2218	M	633.00	86.00	18.70	35.20	15.00	2.80	15	1	24	15	25	18	2	235	58	61	75	50	24	16	5
MZUFBA	2397	F	665.00	71.00	17.80	32.40	10.50	3.10	16	1	23	19	28	17	1	244	-	63	83	53	23	22	-
MZUFBA	2398	F	536.00	73.00	16.10	31.00	10.10	2.20	17	1	22	20	24	16	1	238	53	63	82	44	22	15	4
MZUFBA	2434	F	512.00	66.00	15.60	27.20	9.40	2.20	17	1	19	17	21	16	2	226	50	59	76	49	23	17	6

MZUSP	3118	M	566.00	85.00	23.30	37.20	13.80	3.40	16	0	20	17	22	16	0	238	53	53	75	50	17	21	5
MZUSP	9012	M	739.00	97.00	-	-	-	-	-	-	-	-	-	17	0	232	54	61	79	47	23	20	4
UFRRJ	2638	M	907.00	140.00	-	-	-	-	15	0	19	16	25	-	-	-	52	60	78	49	20	17	5
UFRRJ	3728	M	481.00	65.00	16.96	28.66	10.34	1.99	16	0	21	20	23	16	0	231	48	55	77	48	22	20	5
UFRRJ	7027	M	649.00	-	20.10	34.76	13.35	3.48	18	0	19	16	23	22	2	236	-	59	83	48	21	21	-
ZUFRJ	422	F	1,371.00	157.00	34.80	58.30	28.10	5.30	17	1	21	16	24	14	1	238	56	56	81	45	18	22	6
ZUFRJ	800	M	-	-	9.40	28.00	10.20	2.20	17	1	21	18	25	17	2	238	54	67	89	51	24	14	4
ZUFRJ	963	F	457.00	69.00	16.10	26.30	9.30	2.50	16	0	20	13	24	15	0	235	55	48	78	46	24	21	6
ZUFRJ	1509	F	662.00	79.00	-	33.60	-	-	20	0	24	17	25	20	1	238	51	63	89	48	19	16	4
