# Supplementary material for: The good, the bad and the boa: An unexpected new species of a true boa revealed by morphological and molecular evidence
Source: PLoS One. 2024 Apr 17;19(4):e0298159. doi: 10.1371/journal.pone.0298159 (PMC11023597; doi:10.1371/journal.pone.0298159)
Supplement: S2 Table — (PDF) [file pone.0298159.s005.pdf]

S7 Meristic and morphometric data of the examined specimens of *Boa occidentalis*.

AD = anterior dorsal rows; CIRC = number of circumorbital scales; EMD = eye-mouth distance; F = female; GUL = Gulars; HH = head height; HL = head length; HW = head width; IL = Infralabials; INTR = intrasupraocular scales; M = male; MD = midbody dorsal rows; NS = number of saddles; NTS = number of tail spots; PD = posterior dorsals rows; PV = pre-ventrals; SC = subcaudals; SL = supralabial scales; SUB = subocular scales; SVL = snout-vent length; TD = tail dorsals; TL = tail length; V = ventral.

Acronym	Number	SE-	SVL	TL	HW	HL	HH	EMD	CIRC	SUB	SL	INTR	IL	GUL	PV	V	SC	AD	MD	PD	TD	NS	NTS
AMNH R-	141664	M	578.00	66.00	16.13	30.77	10.83	2.52	17	1	21	17	22	20	0	254	47	65	83	52	21	26	2
FML	40	M	1,749.00	164.00	35.50	59.30	24.80	5.10	16	1	22	14	23	18	0	255	45	62	80	49	20	25	-
FML	63	M	1,989.00	194.00	38.10	65.10	25.30	6.40	13	1	21	16	25	16	2	253	43	63	77	49	21	27	-
FML	225	F	862.00	91.00	22.30	37.90	12.70	3.70	16	1	19	17	26	18	0	256	43	63	86	48	20	30	-
FML	1700	F	1,364.00	129.00	35.70	46.90	16.90	3.80	16	1	19	16	23	15	1	252	42	60	78	47	19	26	5
FML	1905	F	1,556.00	138.00	32.60	51.40	28.60	4.70	15	1	21	15	24	17	0	-	42	53	78	47	18	30	3
FML	2196	M	842.00	98.00	25.40	35.80	12.70	3.10	16	2	20	16	25	16	0	245	47	62	80	46	22	20	4
FML	2210	F	2,538.00	206.00	67.50	92.30	25.20	8.80	15	1	21	16	24	17	0	251	43	63	79	46	18	24	4
FML	2227	M	1,296.00	141.00	49.10	17.80	5.60	16.10	17	0	18	17	24	18	1	248	51	64	81	48	20	26	-
FML	2261-1	M	642.00	74.00	17.40	31.90	11.40	2.70	16	1	21	15	23	16	2	247	47	60	85	49	22	26	2
FML	2261-2	F	644.00	68.00	17.70	31.80	10.20	2.90	18	1	21	17	26	16	2	244	43	66	82	55	23	27	5
FML	2261-3	F	632.00	74.00	13.80	32.40	12.60	2.10	17	1	20	18	25	17	2	250	45	65	84	52	20	27	2
FML	2262-2	F	627.00	67.00	17.40	31.60	11.70	2.40	17	1	23	16	27	18	2	245	46	67	83	51	20	26	3
FML	2262-3	F	627.00	68.00	17.80	30.60	11.30	2.20	16	1	21	16	24	17	2	253	45	68	82	49	23	25	4
FML	2262-4	F	608.00	60.00	15.30	30.30	10.30	2.10	16	0	18	17	24	16	0	252	41	59	82	46	20	24	4
FML	2278	F	1,355.00	115.00	28.60	51.10	18.10	4.80	17	1	20	18	24	18	1	250	43	63	80	49	19	20	2
FML	2279	M	1,867.00	228.00	-	-	-	-	14	1	22	17	25	16	3	249	48	66	84	45	23	26	4
FML	2290	F	1,729.00	145.00	35.20	57.30	18.80	5.70	17	1	21	15	23	15	1	248	41	56	80	48	20	28	3

FML	6410	M	1,424.00	134.00	34.50	58.30	20.90	5.70	16	1	22	17	25	18	0	250	50	62	79	48	22	25	2
FML	6527	M	1,495.00	-	31.10	50.30	20.40	4.90	14	1	21	14	26	17	2	247	45	64	78	46	20	27	1
FML	7360	M	1,819.00	219.00	40.60	60.00	23.30	5.30	14	1	20	14	23	16	0	250	47	61	75	46	20	27	5
FML	9470	M	1,363.00	152.00	27.00	51.10	17.70	3.90	18	1	21	16	25	15	1	-	49	61	61	48	22	26	3
FML	9614	F	2,335.00	174.00	44.80	79.60	27.50	7.10	17	1	19	15	25	17	1	247	43	67	85	49	20	26	4
FML	14016	M	1,996.00	239.00	43.10	67.70	26.70	6.40	16	1	21	17	25	17	4	245	48	62	78	49	20	23	0
FML	14303	M	1,614.00	149.00	33.00	56.20	25.90	6.70	15	1	18	16	24	20	1	243	48	62	86	-	24	27	2
FML	15889	M	2,105.00	234.00	44.50	66.50	25.30	6.50	13	1	22	14	24	16	2	247	48	61	77	45	21	28	4
FML	17629	F	958.00	97.00	13.60	41.10	12.70	2.70	16	1	18	17	25	16	1	252	44	66	85	49	18	26	3
FML	17929	M	1,794.00	201.00	38.00	57.90	21.90	5.10	15	1	21	15	22	19	0	250	46	65	85	49	20	24	2
FML	18372	F	885.00	93.00	22.50	41.10	13.10	2.40	15	1	20	18	25	19	2	251	44	63	83	48	20	27	5
FML	23193	M	524.00	69.00	-	-	-	-	15	1	-	17	-	14	2	246	44	62	78	50	20	24	3
FML	24503	M	621.00	72.00	19.50	34.10	12.30	2.20	17	1	18	16	23	20	0	247	48	64	82	52	24	23	3
FML	28405	F	1,814.00	132.00	42.60	59.40	23.90	5.70	16	1	20	17	27	18	1	245	44	63	83	52	21	26	3
IBSP	10139	M	500.00	62.00	17.11	29.76	1.94	2.45	17	0	19	16	25	18	1	245	44	58	78	46	21	25	4
IBSP	72731	M	1,459.00	189.00	39.23	69.09	27.49	7.53	16	1	20	16	25	19	3	244	44	65	73	50	20	24	-
IBSP	78246	F	1,300.00	138.00	-	-	-	-	17	1	22	16	23	18	1	248	40	62	80	47	19	26	2
IBSP	78325	F	1,061.00	135.00	35.40	69.70	23.50	6.61	14	1	20	17	27	17	1	246	41	65	77	46	21	26	2
IBSP	78352	M	1,449.00	152.00	41.36	73.50	23.29	7.06	16	1	21	16	24	16	3	247	41	67	79	48	21	24	4
IBSP	83333	F	2,024.00	215.00	51.00	83.80	29.90	10.30	-	1	21	17	27	17	0	252	47	61	80	50	23	27	5
MACN	7612	M	569.00	63.00	16.40	28.10	7.90	2.40	16	1	21	17	24	21	2	250	44	55	80	48	21	28	0
MACN	8444	M	676.00	79.00	19.30	31.80	11.30	2.30	17	1	22	19	25	18	1	248	47	58	79	46	19	26	5
MACN	9452	F	-	-	-	-	-	-	17	1	23	15	24	17	0	252	-	59	81	47	18	27	0
MACN	23731	M	1,918.00	216.00	44.10	71.70	23.10	6.50	17	1	19	17	24	19	1	248	46	65	80	47	20	26	5
MACN	34347	F	577.00	60.00	16.30	27.40	9.80	2.40	17	1	20	19	25	17	1	245	41	56	80	48	19	-	3
MACN	34421	-	-	-	-	-	-	-	12	1	19	15	22	19	-	-	-	59	83	48	-	28	0
MACN	34425	-	-	-	-	-	-	-	16	1	22	15	27	15	-	-	-	61	74	43	18	28	-
MACN	35449	F	739.00	77.00	20.10	34.60	11.60	2.40	10	1	22	16	26	14	1	245	42	62	76	45	19	26	3

MACN	39720	-	-	-	-	-	-	-	14	1	-	16	23	20	-	247	45	63	81	51	20	26	2
MACN	39744	M	1,653.00	197.00	41.20	64.70	22.90	6.40	17	1	21	17	25	16	0	250	49	60	79	45	19	24	1
MACN	39749	F	1,551.00	220.00	48.20	74.40	27.50	-	16	1	19	18	24	18	0	253	42	65	88	47	20	26	3
MACN	39850	M	1,646.00	243.00	58.00	75.90	38.40	7.20	17	1	20	17	26	19	1	241	49	66	77	50	20	25	3
MACN	40025	-	-	-	-	-	-	-	-	-	-	-	-	-	-	-	-	-	-	-	-	-	-
MACN	40026	F	-	-	-	-	-	-	-	-	-	-	-	-	-	246	-	-	-	-	-	-	-
MACN	45425	M	-	-	-	-	-	-	16	1	19	14	24	17	-	246	47	59	72	-	22	27	5
MACN	47708	F	658.00	78.00	19.60	30.50	10.10	3.20	16	1	20	15	21	16	2	245	44	59	77	48	21	30	5
MACN	47709	F	1,284.00	119.00	26.60	47.80	14.80	3.40	17	1	21	18	25	17	1	253	46	61	78	49	20	27	6
MACN	47710	M	807.00	88.00	22.80	36.10	11.90	2.50	15	1	20	15	26	17	2	247	47	60	82	52	19	26	2
MACN	48667	F	1,771.00	171.00	34.60	62.40	21.80	5.90	15	1	23	16	23	17	1	248	44	61	77	49	21	27	5
MACN	48669	M	1,454.00	143.00	33.70	48.50	16.90	4.60	18	1	19	14	27	-	2	248	47	57	81	50	21	26	3
MACN	48670	F	2,046.00	165.00	43.10	78.80	25.40	5.70	18	1	20	13	24	16	2	248	44	60	82	45	21	27	5
MACN	48671	M	2,069.00	-	46.10	73.00	26.80	6.60	17	1	21	15	22	16	-	240	-	60	75	47	22	23	-
MACN	48674	F	2,899.00	239.00	60.20	81.60	34.20	11.40	14	1	22	14	22	16	1	248	44	59	77	49	19	27	4
MACN	48675	F	679.00	71.00	23.90	33.60	12.20	3.90	16	1	19	15	24	21	1	246	43	62	84	52	21	28	4
MACN	48676	F	79.00	76.00	20.50	31.10	11.80	2.30	16	0	20	14	23	15	1	246	47	62	81	45	20	25	3
MCN	10046	M	901.00	113.00	22.60	42.10	14.20	3.60	19	1	22	17	26	18	0	240	51	62	87	53	24	29	6
MCP	17643	F	1,864.00	160.00	38.30	68.90	21.70	7.10	16	1	18	16	25	16	1	246	42	60	78	52	18	26	4
MCP	18303	F	2,261.00	179.00	46.20	79.70	30.50	6.60	18	1	18	18	25	19	0	253	41	66	85	49	21	28	0
MNHNP	246	F	650.00	79.00	21.40	30.30	12.30	3.30	20	1	20	17	26	17	2	246	46	66	84	50	21	24	3
MNHNP	2986	F	2,282.00	-	55.50	85.80	25.50	7.90	15	1	19	-	25	18	1	250	-	59	76	44	17	24	4
MNHNP	3789	M	1,635.00	163.00	41.80	58.80	17.50	6.10	17	1	21	17	24	15	2	244	46	57	77	46	16	25	4
MNHNP	3979	M	1,958.00	234.00	46.90	71.90	20.20	6.00	15	1	22	20	23	19	1	250	46	-	-	-	20	-	0
MNHNP	4045	M	662.00	83.00	20.70	34.10	9.50	2.90	17	1	20	18	25	18	1	243	47	63	79	48	19	25	4
MNHNP	5132	F	2,035.00	196.00	39.40	73.50	25.80	8.50	14	1	22	18	24	15	2	252	44	61	77	47	20	28	4
MNHNP	5134	F	528.00	68.00	17.20	28.10	10.10	2.90	14	1	19	15	24	16	1	247	46	56	72	47	20	25	4
MNHNP	5135	M	544.00	68.00	15.40	25.50	10.10	2.40	17	1	22	17	26	19	1	243	49	69	84	47	20	24	3

MNHNP	7241	F	2,856.00	150.00	39.90	64.10	18.00	6.70	14	1	20	15	23	20	2	247	42	58	75	52	23	25	1
MNHNP	9434	F	1,901.00	191.00	41.20	71.50	21.20	5.90	18	0	22	21	25	15	0	252	44	60	69	50	20	29	0
MVZ	128174	M	1,782.00	160.00	38.67	64.37	22.59	5.97	18	1	23	18	25	21	2	246	48	65	84	52	22	27	5
UMMZ	94098	M	502.00	63.00	16.58	27.40	10.06	2.38	16	1	21	19	25	18	1	241	44	57	83	46	21	21	4
