# Supplementary material for: The good, the bad and the boa: An unexpected new species of a true boa revealed by morphological and molecular evidence
Source: PLoS One. 2024 Apr 17;19(4):e0298159. doi: 10.1371/journal.pone.0298159 (PMC11023597; doi:10.1371/journal.pone.0298159)
Supplement: S3 Table — (PDF) [file pone.0298159.s006.pdf]

S8 Meristic and morphometric data of the examined specimens of *Boa orophias*.

AD = anterior dorsal rows; CIRC = number of circumorbital scales; EMD = eye-mouth distance; F = female; GUL = Gulars; HH = head height; HL = head length; HW = head width; IL = Infralabials; INTR = intrasupraocular scales; M = male; MD = midbody dorsal rows; NS = number of saddles; NTS = number of tail spots; PD = posterior dorsals rows; PV = pre-ventrals; SC = subcaudals; SL = supralabial scales; SUB = subocular scales; SVL = snout-vent length; TD = tail dorsals; TL = tail length; V = ventral.

Acronym	Number	SE-	SVL	TL	HW	HL	HH	EMD	CIRC	SUB	SL	INTR	IL	GUL	PV	V	SC	AD	MD	PD	TD	NS	NTS
USNM	418	M	1,662.00	260.00	28.82	59.10	19.05	5.04	17	0	19	15	22	18	0	262	66	56	71	42	19	28	7
KU	260009	M	1,453.00	280.00	31.57	58.35	19.24	5.05	16	0	21	15	22	15	0	274	68	58	75	46	20	26	7
KU	260010	M	1,603.00	275.00	36.28	66.03	20.95	5.86	16	0	21	14	25	20	-	275	65	58	73	44	20	29	8
KU	260011	F	1,640.00	235.00	37.98	70.18	23.05	6.87	16	0	20	17	24	17	1	272	64	57	74	44	20	26	8
KU	260012	M	671.00	103.00	17.99	30.21	9.88	2.46	18	0	21	17	24	18	1	276	69	53	76	48	22	29	9
KU	260013	F	1,669.00	225.00	40.57	69.05	19.17	5.98	15	0	20	17	23	16	0	275	63	58	72	45	18	29	7
MCZ R-	6659	M	1,388.00	201.00	26.73	51.94	19.23	4.87	17	0	20	17	24	18	3	280	63	56	70	42	20	30	7
MCZ R-	6711	M	1,347.00	230.00	29.44	44.94	15.73	3.88	15	0	19	16	22	18	1	270	68	56	72	43	21	28	7
MCZ R-	75842	F	1,830.00	218.00	32.61	59.31	19.31	5.94	14	0	22	17	24	17	0	265	66	60	76	46	19	31	6
MCZ R-	75843	F	1,403.00	171.00	24.73	51.68	15.99	4.29	15	0	19	17	23	16	2	270	64	55	72	44	19	29	8
MCZ R-	75847	M	2,372.00	304.00	38.47	72.09	22.97	6.95	17	0	21	16	22	16	1	265	68	60	74	44	20	28	7
MCZ R-	75848	F	2,370.00	252.00	40.70	68.24	25.13	7.37	14	0	20	16	24	19	0	277	63	57	73	44	18	30	7
NMR	S/N	M	1,375.00	249.00	29.23	51.11	23.57	3.71	18	0	19	14	21	-	-	274	65	57	70	42	-	-	6
