# Supplementary material for: The good, the bad and the boa: An unexpected new species of a true boa revealed by morphological and molecular evidence
Source: PLoS One. 2024 Apr 17;19(4):e0298159. doi: 10.1371/journal.pone.0298159 (PMC11023597; doi:10.1371/journal.pone.0298159)
Supplement: S4 Table — (PDF) [file pone.0298159.s007.pdf]

S9 Meristic and morphometric data of the examined specimens of *Boa nebulosa*.

AD = anterior dorsal rows; CIRC = number of circumorbital scales; EMD = eye-mouth distance; F = female; GUL = Gulars; HH = head height; HL = head length; HW = head width; IL = Infralabials; INTR = intrasupraocular scales; M = male; MD = midbody dorsal rows; NS = number of saddles; NTS = number of tail spots; PD = posterior dorsals rows; PV = pre-ventrals; SC = subcaudals; SL = supralabial scales; SUB = subocular scales; SVL = snout-vent length; TD = tail dorsals; TL = tail length; V = ventral.

Acronym	Number	SE-	SVL	TL	HW	HL	HH	EMD	CIRC	SUB	SL	INTR	IL	GUL	PV	V	SC	AD	MD	PD	TD	NS	NTS
CAS	113542	F	1,973.00	170.00	26.20	56.30	21.10	4.10	18	0	20	14	24	17	2	265	57	51	68	40	22	0	5
USNM	145440	F	2,063.00	182.00	37.48	67.89	21.85	5.36	19	0	21	14	25	18	1	264	57	55	66	39	20	18	5
USNM	153847	M	1,529.00	176.00	27.72	50.86	16.44	4.97	20	0	19	13	23	17	2	262	57	56	61	42	20	21	5
USNM	153848	F	1,051.00	112.00	23.59	40.52	15.79	3.72	19	0	20	13	21	17	-	-	56	50	66	41	20	-	5
USNM	153849	F	735.00	88.00	20.76	35.24	12.89	2.95	16	0	20	16	22	18	2	261	55	53	64	39	19	17	6
USNM	153850	F	1,553.00	154.00	-	-	-	-	-	0	20	-	21	16	0	267	56	51	59	38	20	0	5
USNM	156875	M	1,058.00	128.00	22.20	40.30	14.30	3.80	13	0	20	15	21	17	1	261	56	55	69	43	19	19	5
USNM	160226	F	705.00	98.00	17.50	28.10	10.20	2.60	17	0	21	14	21	18	2	265	56	55	68	39	20	31	6
USNM	160620	F	1,500.00	166.00	46.61	80.20	26.05	-	17	0	22	15	23	15	3	262	55	57	67	42	20	0	6
USNM	161004	F	1,965.00	186.00	34.70	65.60	21.30	6.03	17	0	22	16	23	16	-	-	54	55	-	39	20	18	4
USNM	161005	-	1,431.00	202.00	23.40	49.10	18.10	5.10	16	0	20	12	21	17	0	261	56	51	64	40	21	20	6
USNM	161007	-	1,954.00	175.00	39.40	62.10	17.80	5.40	20	1	22	18	23	-	-	257	52	-	66	41	21	0	0
USNM	161008	F	2,080.00	178.00	40.26	70.77	20.69	7.11	14	1	20	15	22	17	0	260	52	54	66	40	21	16	5
USNM	161009	-	1,480.00	210.00	29.30	52.80	16.90	4.40	17	0	21	14	23	16	2	256	58	54	65	40	21	18	8
MVZ	211940	M	1,160.00	172.00	26.54	50.67	15.97	4.04	18	1	20	11	22	15	1	264	52	54	64	38	20	18	5
MVZ	211941	M	1,302.00	224.00	26.80	55.73	17.43	4.08	15	0	22	16	20	17	0	264	59	53	64	36	19	19	5
KU	260001	F	936.00	110.00	23.23	38.67	12.48	3.24	19	0	21	18	22	20	1	263	58	55	69	41	21	20	5
KU	260004	F	1,282.00	168.00	-	-	-	-	18	0	21	17	23	18	0	263	56	56	69	42	18	20	6

KU	260005	F	1,696.00	211.00	-	-	-	-	17	0	20	-	22	15	-	-	58	-	64	42	18	0	5
KU	260006	M	1,363.00	213.00	-	-	-	-	18	0	20	15	21	18	1	261	59	53	65	40	20	27	6
KU	260007	F	959.00	-	25.23	38.47	13.76	3.32	19	0	20	15	22	18	2	256	-	60	67	40	21	17	-
KU	260008	M	887.00	117.00	21.53	36.90	11.76	3.02	21	0	19	18	22	16	1	267	60	53	64	38	21	23	6
UMMZ	203783	M	554.00	79.00	15.73	25.93	10.74	2.08	20	0	22	16	23	19	3	269	63	54	67	40	22	30	6
MCZ R-	6106	M	569.00	83.00	16.02	27.84	10.93	1.98	19	0	22	15	23	17	3	262	60	57	66	42	22	29	7
MCZ R-	58772	F	1,508.00	164.00	26.32	56.07	18.32	5.43	20	0	21	17	22	18	1	269	55	55	63	38	19	22	4
MCZ R-	65492	F	1,764.00	210.00	34.93	67.17	19.27	5.50	16	0	21	16	22	16	1	264	58	55	68	38	21	0	6
MCZ R-	65493	F	1,714.00	177.00	34.57	55.72	19.54	5.14	16	0	19	15	22	17	3	263	58	54	66	39	19	0	5
MCZ R-	65494	F	1,637.00		35.20	52.77	16.27	5.09	19	0	23	16	23	16	1	267		52	65	39	19	0	
MCZ R-	65495	F	2,184.00		49.31	71.96	21.60	7.08	17	0	22	18	23	20	1	256		58	61	41	19	18	
MCZ R-	74371	M	1,473.00	217.00	14.03	50.75	14.66	3.02	19	0	19	16	21	18	2	265	59	54	62	37	20	12	6
MCZ R-	176903	F	1,901.00	195.00	34.64	62.93	20.30	5.43	21	0	21	20	23	18	1	265	62	58	69	41	21	0	4
