# Supplementary material for: The good, the bad and the boa: An unexpected new species of a true boa revealed by morphological and molecular evidence
Source: PLoS One. 2024 Apr 17;19(4):e0298159. doi: 10.1371/journal.pone.0298159 (PMC11023597; doi:10.1371/journal.pone.0298159)
Supplement: S5 Table — (PDF) [file pone.0298159.s008.pdf]

S10 Pinpoint of vouchers and samples used to compose the distribution map of *Boa atlantica* sp nov (Fig 6). Holotype is marked in bold.

ACRONYM	NUMBER	STATE/PROVINCE	MUNICIPALITY/DEPARTMENT	LOCALITY	LATITUDE	LONGITUDE	Map Number
CHBEZ	1196	RN	Caicó		-6.456440105	-37.10161976	1
CHBEZ	1254	RN	Serra Negra do Norte		-6.667064603	-37.394818	2
MCP (RS)	13737	PB	Santa Gertrudes		-6.94912771	-37.39454377	3
MNRJ	3940	AL	Passo do Camaragibe	Fazenda Santa Justina	-9.27458100	-35.46799400	4
IVB	2953	AL	Quebrângulo	Pedra Talhada	-9.32354706	-36.47722208	5
CHBEZ	42	AL	Rio Largo		-9.482133048	-35.83668461	6
MZUFBA	1165	AL	Piranhas	UHE - Xingó	-9.62355920	-37.75039291	7
MZUFBA	1166	AL	Piranhas	UHE - Xingó	-9.62355920	-37.75039291	8
MZUFBA	1167	AL	Piranhas	UHE - Xingó	-9.62355920	-37.75039291	9
MZUFBA	1168	AL	Piranhas	UHE - Xingó	-9.62355920	-37.75039291	10
CZGB	7949	BA	Monte Santo		-10.44015774	-39.33270595	11
MZUSP	9012	SE	Santo Amaro das Brotas		-10.78333330	-37.06666670	12
IBSP	79163	BA	Feira de Santana		-12.22928425	-38.96003310	13
MZUFBA	1968	BA	Mata de São João		-12.52997079	-38.29729932	14
MZUFBA	379	BA	São Francisco do Conde	Sítio Madrugada	-12.66545400	-38.59003500	15

MZUESC	4696	BA	Conceição do almeida		-12.77802032	-39.17094645	16
IBSP	79031	BA	Salvador		-13.00491501	-38.50712429	17
CZGB	3051	BA	Salvador		-13.01477191	-38.48806148	18
MZUFBA	2218	BA	Salvador	FACOM, Campus Ondina - UFBA	-13.01477191	-38.48806148	19
MZUFBA	2397	BA	Salvador	Pituaçu	-13.01477191	-38.48806148	20
MZUFBA	2398	BA	Salvador	Instituto de Farmácia, Campus Ondina - UFBA	-13.01477191	-38.48806148	21
MZUFBA	2434	BA	Salvador	Ondina	-13.01477191	-38.48806148	22
IBSP	79033	BA	Salvador		-13.01477191	-38.48806148	23
MZUESC	6310	BA	Presidente Tancredo Neves		-13.47084177	-39.42405983	24
CZGB	2713	BA	Valença		-13.48760340	-39.04325436	25
MNRJ	6361	BA	Ituaçú		-13.80740715	-41.31098153	26
CZGB	1315	BA	Camamu		-13.94707634	-39.10056972	27
CZGB	2712	BA	Marau		-14.10553067	-39.02495176	28
IBSP	79086	BA	Itacaré		-14.27824929	-38.99484155	29
CZGB	4862	BA	Ilhéus		-14.79332027	-39.04318429	30
MNRJ	6362	BA	Ilhéus	Salobrinho	-14.80117900	-39.17600200	31
MCN (RS)	5708	BA	Vitória da Conquista		-14.84800481	-40.83980954	32
CZGB	8514	BA	Barra do Choça		-14.86427304	-40.57637299	33

CZGB	1291	BA	Uma		-15.29420806	-39.07471289	34
CZGB	2820	BA	Ribeirão do Largo		-15.46405963	-40.74385976	35
MNRJ	12692	BA	Itamaraju	Guarani	-17.03333300	-39.53333300	36
CZGB	705	BA	Itamaraju		-17.04233760	-39.53303988	37
MZUFBA	1514	BA	Itamaraju	Estrada Guarani - Parque Nacional do Descobrimento	-17.04233760	-39.53303988	38
MNRJ	16575	BA	Prado	Parque Nacional do Descobrimento	-17.08333300	-39.30000000	39
MBML	1934	BA	Nova Viçosa	Estrada Mucuri-Nova Viçosa	-17.89339178	-39.37201154	40
MBML	1937	ES	Pedro Canário	Fazenda Alegria	-18.03027800	-40.15055600	41
MBML	2257	BA	Mucuri		-18.08638900	-39.55083300	42
CZGB	8389	BA	Mucuri		-18.08853974	-39.54754589	43
MBML	1932	BA	Mucuri	Cruzelândia. Fazenda Pombal. Próximo ao Rio Mucuri. Em uma derrubada de cachoeira. Solo arenososo.	-18.08853974	-39.54754589	44
MBML	1935	BA	Mucuri	Rio Mucuri. Fazenda Martinica. 3KM do rio abaixo da ponte. Em furca de árvore a uns dois metros da água.	-18.08853974	-39.54754589	45
MNRJ	23880	ES	Conceição da Barra	Floresta Nacional do Rio Preto	-18.38333300	-39.81666700	46
MBML	1933	ES	Conceição da Barra	Rodovia Pinheiro-BR 101. Próximo a localidade de	-18.59333300	-39.73222200	47

				Sayonara			
MNRJ	23882	ES	São Mateus	Campus CEUNES/ UFES, Bairro de Litorânea	-18.66666700	-39.85000000	48
MNRJ	23881	ES	São Mateus	Estrada para Guriri, próximo ao Pontal	-18.70000000	-39.85000000	49
IBSP	52164	ES	São Mateus		-18.71844697	-39.85820060	50
IBSP	52165	ES	São Mateus		-18.71844697	-39.85820060	51
MNRJ	23879	ES	Linhares	Reserva Biológica de Comboios	-19.55000000	-40.05000000	52
MBML	2013	ES	Santa Teresa	Valsugana Velha	-19.93555600	-40.60027800	53
MBML	2853	ES	Santa Teresa	Rua Horlando Bonfim, Vila Nova.	-19.93560000	-40.60030000	54
MBML	2855	ES	Santa Teresa		-19.93560000	-40.60030000	55
IBSP	47431	ES	Cariacica		-20.29467780	-40.39055910	56
MNRJ	9565	ES	Vitória	Morro da Gamela, Santa Lúcia	-20.29730300	-40.30140000	57
MNRJ	25413	ES	Vitória	Gascav	-20.29938700	-40.32792600	58
MBML	2217	ES	Vitória	Fonte Grande. Mirante da cidade.	-20.31274900	-40.33722400	59
MBML	1936	ES	Vitória	Santa Lúcia. Morro da Santa Lúcia	-20.31944400	-40.33777800	60
MBML	2218	ES	Vitória	Fonte Grande. Casa do Sr Domingos.	-20.31944400	-40.33777800	61
MBML	2220	ES	Vitória	Fonte Grande	-20.31944400	-40.33777800	62
MBML	58	ES	Vila Velha		-20.33837410	-40.29395664	63

MNRJ	23361	ES	Guarapari	Setiba, Rodosol - Rodovia que cruza o Parque Estadual Paulo César Vinha	-20.61666700	-40.43333300	64
MNRJ	24903	ES	Guarapari	Setiba	-20.63319900	-40.44051800	65
MBML	2180	ES	Guarapari	Rodovia do Sol	-20.65800000	-40.51100000	66
MBML	2001	ES	Guarapari	Restinga	-20.66666700	-40.49750000	67
MBML	2178	ES	Guarapari	Rodovia do Sol	-20.66666700	-40.49750000	68
MBML	2179	ES	Guarapari	Rodovia do Sol	-20.66666700	-40.49750000	69
MBML	2183	ES	Guaçuí		-20.77560000	-41.67940000	70
MBML	2315	ES	Anchieta	Gasotudo Ramal - GASCAV UTG- Sul Capixaba	-20.80580000	-40.64560000	71
MBML	2258	ES	Anchieta	Unidade de Tratamento de Gás Sul Capixaba.	-20.80583300	-40.64555600	72
IBSP	79063	ES	Cachoeiro do Itapemerim		-20.85011235	-41.11191881	73
IBSP	79063	ES	Cachoeiro do Itapemirim		-20.85332115	-41.12904923	74
MNRJ	14172	RJ	Porciúncula	Fazenda Vargem Grande	-20.96666700	-42.03333300	75
UFRRJ	2638	ES	Presidente Kennedy		-21.09656700	-41.04596900	76
UFRRJ	3728	ES	Presidente Kennedy		-21.09656700	-41.04596900	77
MCNR (puc-mg)	4828	RJ	Bom Jesus de Itabapoana	PCH Calheiros	-21.12099000	-41.71870700	78
MBML	2097	RJ	São Francisco de Itabapoana		-21.23416700	-41.12194400	79

MZHUFV	2267	MG	Laranjal		-21.36362961	-42.48067429	80
IBSP	79481	RJ	São João da Barra		-21.64045775	-41.05034815	81
IBSP	4620	RJ	São Fidelis		-21.64891700	-41.74563100	82
MNRJ	400	RJ	Campos dos Goytacazes	Lagoa Feia. 12 km ao sul de Campos dos Goytacazes	-21.96085800	-41.29070200	83
MNRJ	19594	RJ	Quissamã	Fazenda Trindade	-22.11666700	-41.50000000	84
MNRJ	16436	RJ	Conceição de Macabu	Hotel Fazenda Carrapeta	-22.14088900	-41.84673600	85
MNRJ	18269	RJ	Carapebus	Parque Nacional da Restinga de Jurubatiba	-22.21666700	-41.50000000	86
MNRJ	26802	RJ	Carapebus	Parque Nacional da Restinga de Jurubatiba	-22.25000000	-41.65000000	87
MNRJ	20700	RJ	Teresópolis	road BR-116 km 86.5	-22.40000000	-42.96666700	88
MNRJ	20700	RJ	Teresópolis		-22.43035356	-42.97324944	89
MNRJ	15198	RJ	Cachoeiras de Macacu	Parte alta da serra	-22.45000000	-42.65000000	90
MNRJ	10117	RJ	Rio das Ostras		-22.45123900	-41.95335300	91
UFRRJ	7027	RJ	Rio das Ostras		-22.45917100	-41.93378500	92
IVB	3422	RJ	Cachoeiras de Macacu		-22.46757087	-42.66316436	93
MNRJ	18535	RJ	Guapimirim	Rodovia BR116 km 100,1 - Pista Além Paraíba - Teresópolis	-22.50000000	-42.91666700	94
MNRJ	14238	RJ	Guapimirim		-22.56666700	-43.00000000	95
MNRJ	14250	RJ	Guapimirim		-22.56666700	-43.00000000	96

ZUFRJ	963	RJ	Silva Jardim	Distrito Gaviões	-22.633723	-42.408408	97
MNRJ	25057	RJ	Itaboraí	Complexo Petroquímico do Rio de Janeiro (COMPERJ)	-22.65457100	-42.85669959	98
MNRJ	26324	RJ	Itaboraí	Complexo Petroquímico do Rio de Janeiro (COMPERJ)	-22.65457100	-42.85669959	99
MZUSP	3118	RJ	Rio Bonito		-22.71127150	-42.62599784	100
MHNCI	1454	RJ	Itaboraí	Venda das Pedras	-22.74586302	-42.86034343	101
MHNCI	1922	RJ	Itaboraí	Venda das Pedras	-22.74586302	-42.86034343	102
MNRJ	26213	RJ	Nova Iguaçu	Av. Dr. Barros Jr	-22.75158900	-43.44792700	103
MNRJ	24860	RJ	Nova Iguaçu	Jardim Nova Era	-22.75820600	-43.47388800	104
MNRJ	14202	RJ		Estrada Cabo Frio - Búzios	-22.78333300	-41.95000000	105
MNRJ	25951	RJ	Rio de Janeiro	Ilha do Governador	-22.80429500	-43.24954073	106
MNRJ	25952	RJ	Rio de Janeiro	Ilha do Governador. Aeroporto Tom Jobim	-22.80429500	-43.24954073	107
MNRJ	26350	RJ	Rio de Janeiro	Ilha do Governador. Aeroporto Tom Jobim	-22.80429500	-43.24954073	108
MNRJ	9449	RJ	Rio de Janeiro	Ilha do Governador, Instituto de Pesquisas da Marinha	-22.82088500	-43.18756400	109
MNRJ	26585	RJ	Rio de Janeiro	Ilha do Governador	-22.82088500	-43.18756400	110
MNRJ	17547	RJ	Rio de Janeiro	Parque Natural Municipal da Serra do Mendanha	-22.83333300	-43.48333300	111
MNRJ	15199	RJ	Rio de Janeiro	Irajá	-22.83333300	-43.31666700	112

MNRJ	17353	RJ	Iguaba Grande	Entorno do Núcleo Experimental de Iguaba Grande (NEIG-UFF)	-22.83333300	-42.16666700	113
MNRJ	18294	RJ	Iguaba Grande	Núcleo Experimental de Iguaba Grande (NEIG-UFF)	-22.83333300	-42.16666700	114
MNRJ	22936	RJ	Cabo Frio	Dunas	-22.83333300	-41.98333300	115
MNRJ	13177	RJ	Rio de Janeiro	Parque Natural Municipal da Serra do Mendanha, Bico do Padre	-22.83636100	-43.50336100	116
IVB	3379	RJ	Rio de Janeiro	Deodoro	-22.87665212	-43.22787512	117
MNRJ	25953	RJ	Rio de Janeiro	Del Castilho	-22.88033700	-43.27223600	118
MNRJ	6364	RJ	Cabo Frio		-22.88076467	-42.02007480	119
MCP (RS)	2544	RJ	Niterói		-22.88330000	-43.10360000	120
MCP (RS)	2558	RJ	Niterói		-22.88330000	-43.10360000	121
MCP (RS)	2948	RJ	Niterói		-22.88330000	-43.10360000	122
MCP (RS)	3021	RJ	Niterói		-22.88330000	-43.10360000	123
IVB	3381	RJ	Niterói		-22.89645239	-43.07582317	124
MNRJ	22936	RJ	Cabo Frio		-22.89986224	-42.03351261	125
MNRJ	23573	RJ	Niterói	Pendotiba, Estrada de Pendotiba	-22.90000000	-43.06666700	126
MNRJ	16922	RJ	Niterói	Pendotiba, Estrada de Pendotiba	-22.90000000	-43.06666700	127
MNRJ	10092	RJ	Rio de Janeiro		-22.90845800	-43.41817100	128

MNRJ	26589	RJ	Rio de Janeiro		-22.90845800	-43.41817100	129
MNRJ	26796	RJ	Rio de Janeiro		-22.90845800	-43.41817100	130
MNRJ	8340	RJ	Rio de Janeiro	Água Santa, Rua Monteiro da Luz	-22.91206200	-43.31245700	131
MNRJ	25950	RJ	Rio de Janeiro	Água Santa	-22.91206200	-43.31245700	132
MNRJ	13111	RJ	Maricá	Loteamento Don Felipe Laguna (Itapeba), São José do Imbassaí	-22.91666700	-42.85000000	133
MHNCI	3097	RJ	Maricá	Barra de Maricá, Lagoa	-22.91695534	-42.81982411	134
MNRJ	26886	RJ	Rio de Janeiro	Parque Nacional da Tijuca	-22.93333300	-43.28333300	135
MNRJ	27262	RJ	Rio de Janeiro	Parque Nacional da Tijuca	-22.93333300	-43.28333300	136
MNRJ	19412	RJ	Rio de Janeiro	Cosme velho	-22.93333300	-43.18333300	137
MNRJ	19564	RJ	Rio de Janeiro	Cosme velho	-22.93333300	-43.18333300	138
**MNRJ**	**27242**	**RJ**	**Rio de Janeiro**	**Rio Comprido**	**-22.93416700**	**-43.21025000**	**139**
MNRJ	27243	RJ	Rio de Janeiro	Rio Comprido	-22.93416700	-43.21025000	140
MNRJ	25954	RJ	Rio de Janeiro	Esquinas da rua Farani com Pinheiro Machado, Botafogo	-22.94079800	-43.18254300	141
MNRJ	19740	RJ	Rio de Janeiro	Bairro Jardim Botânico, Horto Grotão	-22.95000000	-43.23333300	142
MNRJ	11205	RJ	Niterói	Itaipu	-22.95000000	-43.03333300	143
ZUFRJ	1509	RJ	Rio de Janeiro	Jacarepaguá (Anil)	-22.95494200	-43.37957700	144
MNRJ	17846	RJ	Niterói	Parque Estadual da Serra da Tiririca	-22.96666700	-43.01666700	145

MNRJ	18960	RJ	Niterói	Parque Estadual da Serra da Tiririca, Itaipú, Córrego dos Colibris	-22.96666700	-43.01666700	146
ZUFRJ	422	RJ	Arraial do Cabo		-22.96763828	-42.02833597	147
MNRJ	23144	RJ	Rio de Janeiro	Estrada Rio Morto	-23.00000000	-43.48333300	148
MNRJ	19564	RJ	Rio de Janeiro		-23.00535296	-43.32784696	149
MNRJ	19740	RJ	Rio de Janeiro		-23.0054308	-43.32784694	150
MNRJ	14200	RJ	Rio de Janeiro	Recreio dos Bandeirantes	-23.01666700	-43.46666700	151
MNRJ	14201	RJ	Rio de Janeiro	Recreio dos Bandeirantes	-23.01666700	-43.46666700	152
MNRJ	22704	RJ	Rio de Janeiro	Recreio dos Bandeirantes	-23.01666700	-43.46666700	153
MNRJ	22705	RJ	Rio de Janeiro	Recreio dos Bandeirantes	-23.01666700	-43.46666700	154
MNRJ	22963	RJ	Rio de Janeiro	Recreio dos Bandeirantes	-23.01666700	-43.46666700	155
ZUFRJ	800	RJ	Ilha Grande		-23.14677500	-44.22784700	156
