# Supplementary material for: The good, the bad and the boa: An unexpected new species of a true boa revealed by morphological and molecular evidence
Source: PLoS One. 2024 Apr 17;19(4):e0298159. doi: 10.1371/journal.pone.0298159 (PMC11023597; doi:10.1371/journal.pone.0298159)
Supplement: S1 File — (PDF) [file pone.0298159.s009.pdf]

S1 Polymerase chain reaction (PCR) protocol for four genes amplification.

Gene Sequence	Initial denaturation	Denaturation	Annealing	Extension	#Cicles	Final Extension
Cyt-*b*	94°C/5min	94°C/40sec	54°C/30sec	72°C/60sec	35	72°C/10min
ND4	94°C/3min	94°C/30sec	48°C/45sec	72°C/30sec	40	72°C/10min
NTF3	94°C/3min	94°C/30sec	46°C/45sec	72°C/30sec	40	72°C/10min
ODC	94°C/2,5min	94°C/30sec	62°C/30sec	72°C/90sec	5	72°C/10min
	94°C/2,5min	94°C/30sec	58°C/30sec	72°C/90sec	10	
	94°C/2,5min	94°C/30sec	55°C/30sec	72°C/90sec	20	
