# Supplementary material for: The good, the bad and the boa: An unexpected new species of a true boa revealed by morphological and molecular evidence
Source: PLoS One. 2024 Apr 17;19(4):e0298159. doi: 10.1371/journal.pone.0298159 (PMC11023597; doi:10.1371/journal.pone.0298159)
Supplement: S2 File — (PDF) [file pone.0298159.s010.pdf]

S2 Specimens examined for morphology.

Details of specimens belonging to different *Boa* taxa examined for the description of *Boa atlantica*. Acronyms are according to Sabaj-Perez MH. Standard symbolic codes for institutional resource collections in herpetology and ichthyology: an online reference. American Society of Ichthyologists and Herpetologists. 2016; 5(3):802–832, except for: CHIFCE = Coleção Herpetológica do Instituto Federal do Ceará, Acaraú, Brazil; CZDP = Coleção Zoológica Delta do Parnaíba da Universidade Federal do Piauí, Parnaíba, Brazil; CZPB/UFAM = Coleção Zoológica Professor Paulo Buhrnheim, Universidade Federal do Amazonas, Manaus, Brazil.

Boa atlantica. **BRAZIL:** Unknown locality: MNRJ 23144. <u>Alagoas:</u> Passo do Camaragibe: Santa Justina Farm: MNRJ 3940; Piranhas: Xingó Hydroelectric Power Plant: MZUFBA 1165–68; Quebrângulo: Pedra Talhada: IVB 2953. <u>Bahia:</u> Barra do Choça: CZGB 8514; Camamu: CZGB 1315; Conceição do almeida: MZUESC 4696; Feira de Santana: IBSP 79163; Ilhéus: CZGB 4862, Salobrinho: MNRJ 6362; Itacaré: IBSP 79086; Itamaraju: CZGB 705, Estrada Guarani, Parque Nacional do Descobrimento: MZUFBA 1514, Guarani: MNRJ 12692; Ituaçú: MNRJ 6361; Marau: CZGB 2712; Mata de São João: MZUFBA 1968; Monte Santo: CZGB 7949; Mucuri: CZGB 8389, MBML 2257, Cruzelândia, Pombal Farm, next to Mucuri River: MBML 1932, Mucuri River, Martinica Farm: MBML 1935; Nova Viçosa: Mucuri-Nova Viçosa Road: MBML 1934; Prado: Parque Nacional do Descobrimento: MNRJ 16575; Presidente Tancredo Neves: MZUESC 6310; Ribeirão do Largo: CZGB 2820; Salvador: CZGB 3051, IBSP 79033, Faculdade de Comunicação, Campus Ondina, Universidade Federal da Bahia (UFBA): MZUFBA 2218, Instituto de Farmácia,

Campus Ondina, UFBA: MZUFBA 2398, 2434; Pituaçu: MZUFBA 2397; São Francisco do Conde: Sítio Madrugada: MZUFBA 379; Uma: CZGB 1291; Valença: CZGB 2713; Vitória da Conquista: MCN 5708. Espírito Santo: Anchieta: Gasotudo Ramal GASCAV Unidade de Tratamento de Gás Sul Capixaba: MBML 2258, 2315; Cachoeiro do Itapemerim: IBSP 79063; Cariacica: IBSP 47431; Conceição da Barra: Floresta Nacional do Rio Preto: MNRJ 23880, Pinheiro-BR 101 Road, next to Sayonara: MBML 1933; Guaçuí: MBML 2183; Guarapari: Restinga: MBML 2001, Rodovia do Sol: MBML 2178, MBML 2179, MBML 2180, Setiba: MNRJ 24903, Setiba, Rodovia do Sol, road that goes across Parque Estadual Paulo César Vinha: MNRJ 23361; Linhares: Reserva Biológica de Comboios: MNRJ 23879; Pedro Canário: Alegria Farm: MBML 1937; Presidente Kennedy: UFRRJ 2638, UFRRJ 3728; Santa Teresa: MBML 2855, Horlando Bonfim street, Vila Nova: MBML 2853, Valsugana Velha: MBML 2013; São Mateus: Campus Universidade Federal do Espírito Santo, Litorânea: MNRJ 23882, Guriri Road, next to Pontal: MNRJ 23881, IBSP 52164, IBSP 52165; Vila Velha: MBML 58; Vitória: Fonte Grande: MBML 2218, 2220, Fonte Grande observatory: MBML 2217, Gascav: MNRJ 25413, Morro da Gamela, Santa Lúcia Reservation: MNRJ 9565, Morro da Santa Lúcia, Santa Lúcia Reservation: MBML 1936. Minas Gerais: Laranjal: MZUFV 2267. Paraíba: Santa Gertrudes: MCP 13737. Rio de Janeiro: Arraial do Cabo: ZUFRJ 422; Bom Jesus de Itabapoana: Calheiros small hydroelectric power plant: MCNR 4828; Cabo Frio: MNRJ 6364, 22936; Cabo Frio road to Búzios: MNRJ 14202; Cachoeiras de Macacu: IVB 3422, MNRJ 15198; Campos dos Goytacazes: Lagoa Feia. 12 km S of Campos dos Goytacazes: MNRJ 400; Carapebus: Parque Nacional da Restinga de Jurubatiba: MNRJ 18269, 26802; Conceição de Macabu: Carrapeta Farm hotel: MNRJ 16436; Guapimirim: MNRJ 14238, 14250, km 100 of the BR116 Highway, road from Além

Paraíba to Teresópolis: MNRJ 18535; Iguaba Grande: surroundings of Núcleo Experimental de Iguaba Grande from Universidade Federal Fluminense: MNRJ 17353, 18294; Ilha Grande: ZUFRJ 800; Itaboraí: Complexo Petroquímico do Rio de Janeiro (COMPERJ): MNRJ 25057, 26324; Venda das Pedras: MHNCI 1454, 1922; Maricá: Barra de Maricá Lake: MHNCI 3097; Loteamento Don Felipe Laguna (Itapeba), São José do Imbassaí: MNRJ 13111; Niterói: IVB 3381, MCP 2544, MCP 2558, 2948, 3021, MNRJ 16922; Itaipú: MNRJ 11205, Parque Estadual da Serra da Tiririca: MNRJ 17846, Córrego dos Colibris: MNRJ 18960; Pendotiba, road Niterói to Pendotiba: MNRJ 23573; Nova Iguaçu: Avenue Dr. Barros Jr: MNRJ 26213, Jardim Nova Era: MNRJ 24860; Porciúncula: Vargem Alegre Farm: MNRJ 14172; Quissamã: Trindade Farm: MNRJ 19594; Rio Bonito: MZUSP 3118; Rio das Ostras: MNRJ 10117, UFRRJ 7027; Rio de Janeiro: MNRJ 10092, 26796, Água Santa: MNRJ 25950, Monteiro da Luz street: MNRJ 8340, Jardim Botânico, Horto Grotão: MNRJ 19740, Cosme Velho: MNRJ 19412, 19564, Del Castilho: MNRJ 25953, Deodoro: IVB 3379, Botafogo, crossroad of Farani and Pinheiro Machado Streets: MNRJ 25954, Mananciais Road: MNRJ 26589, Ilha do Governador: MNRJ 25951, 26585, Instituto de Pesquisas da Marinha: MNRJ 9449, Tom Jobim Airport: MNRJ 25952, 26350, Irajá: MNRJ 15199, Jacarepaguá (Anil): ZUFRJ 1509, Parque Nacional da Tijuca, MNRJ 27262, 26886, Parque Natural Municipal da Serra do Mendanha: MNRJ 17547, Bico do Padre: MNRJ 13177, Recreio dos Bandeirantes: MNRJ 14200, 14201, 22704, 22705, 22963, Rio comprido: MNRJ 27242, 27243; São Fidelis: IBSP 4620; São Francisco de Itabapoana: MBML 2097; São João da Barra: IBSP 79481; Silva Jardim: Distrito Gaviões: ZUFRJ 963; Teresópolis: km 86 from BR-116 Highway: MNRJ 20700. Sergipe: Santo Amaro das Brotas: MZUSP 9012.

Boa constrictor amarali. **BOLIVIA:** Beni: La Cooperativa, Mamoré River, 4 km W: AMNH R-101918; Exaltacíon: Mamoré River, 8 km N: AMNH R-101917; San Joaquin: FMNH 161541. Buena Vista: locality unknown: MACN 39; Santa Cruz de La Sierra: MACN 1794, MACN 3861. Santa Cruz: Buena Vista: UMMZ 68005, UMMZ 68007; Chiquito: Serrania Santiago: FMNH 195915; Sara: CM R2709. **BRAZIL:** locality unknown: CEPB 2877, AMNH R-14549. Bahia: Itambé: MZUESC 4728; Mucuri: Hydroelectric Power Plant Santa Clara: MCN 1720; Goiás: Barro Alto: IBSP 52452, Anglo American: ZUFG 134; Bela Vista de Goiás: CEPB 2104, 2878, MCP 15831; Bonfinópolis: CEPB 734; Caldas Novas: Corumbá I Hydroelectric Power Plant: CEPB 3918–20, 8540–41; Formosa: MZUSP 12544; Goiânia: CEPB 1154; Goianira: CEPB 2100; Guapó: CEPB 3096; Ipameri: IBSP 23359; Jataí: CEPB 1866, CEPB 1867, 2876, 3447; Luziânia: Corumbá IV Hydroelectric Power Plant: CEPB 7208, 7398; Minaçu: Cana Brava Hydroelectric Power Plant: CEPB 5737, 5741, 5744, 5747, 5748, 5752, 5753, 8391, MCP 13030–31, 13036–39, 13438; Nerópolis: CEPB 8599; Niquelândia: IBSP 84445, Serra da Mesa Hydroelectric Power Plant: MZUSP 16743; Palminópolis: CEPB 2101–02; Rio Quente: ZUFG 779; Santa Terezina de Goiás: IBSP 25807; São Francisco de Goiás: CEPB 1827; São Simão: IBSP 15282; Senador Canedo: Vargem Bonita: CEPB 206; Serranópolis: CEPB 2103, 3010; Uruaçu: MCP 1503, Santo Angelo Farm: MCP 1517; Uruana: CEPB 246. Mato Grosso: road between Ernesto and Pau-a-Pique: UFMT 11497, Alto do Sapé: MZUSP 8674. Araputanga: right bank of Jauru River, Peixe River: UFMT 5159; Ombreiras Small Hydroelectric Power Plant: UFMT 6041; Aripuanã: UFMT 5137; Barra do Garças: Pindaíba: MNRJ 6367; Cáceres: MZUSP 1327, ZUEC 46; Cláudia: Iracema Farm: MZUSP 11239; Chapada dos Guimarães: APM Manso Hydroelectric Power Plant: UFMT 623–24, 723, 8871, Road to aeronautics base: UFMT 6679, km 56 from MT 251 Road: UFMT 2637; Cuiabá:

IBSP 46155, UFMT 2609, 10515, 11778, Avenida da Feb, Trevo do Lagarto: UFMT 2606, Santa Cruz: UFMT 7469, Campus of the Universidade Federal de Mato Grosso, Chácara São Sebastião: 7985; Ribeirão do Lipa: UFMT 5472, in front of Residencial Santa Cruz: UFMT 2613, 4891; Km 5 from the Santo Antonio do Leverger Road: UFMT 8560–61; Gaúcha do Norte: IBSP 69285, 74954; Ilha Solteira: Ilha Solteira Hydroelectric Power Plant: IBSP 36184, 37104, 37110, 37105–06, 37839, 37874, 38442, ,38448; Paraná River: IBSP 38814, 38816; Itiquira: MHNCI 14086, UFRGS 6189; Jaciara: Km 531 from the BR-364 Highway: UFMT 6281; Jauru: access road to Module 1: UFMT 9255; Juína: MZUSP 20758; Lambari D'Oeste: MNRJ 24305, Madeira Electrical Transmission Brach: MZUSP 19835; Nossa Senhora do Livramento: UFMT 11768; Nova Ubiratã: right bank of Von den Steinen River; Small Hydroelectric Central ARS Santo A. R. Bonito: UFMT 5227, 5340, road between Small Hydroelectric Central PCH ARS and Santo Antônio do Rio Bonito: UFMT 5443; Pontes e Lacerda: IBSP 49756, MCN 4889; Porto Estrela: Serra das Araras Ecological Station: UFMT 8727; Querência MPEG 22005, Tanguro: MPEG 21670; São Domingos: IBSP 12834; São Félix do Araguaia: IBSP 12044; São José do Rio Claro: MZUSP 11331; Sinop: IBSP 51442, 55163, 3887; Várzea Grande: Marechal Rondon International Airport: UFMT 9119. Mato Grosso do Sul: Aparecida do Taboado: Taboado Port: IBSP 37962, 38675; Aquidauana: Limão Verde Village: ZUFMS 1666, FMNH 9197 (paratype), ZUFMS 1855; Bataiporã: Primavera Farm: MZUSP 10647; Campo Grande: ZUFMS 1854, Parque dos Poderes: MHNCI 6539, Chácara Três Irmãos (Florestal Avenue): ZUFMS 951–52, 987; Corumbá: UFMG 2245, Centro de Controle de Zoonoses: ZUFMS 1135, MS-228 Highway, next to the junction to BR 262 Highway: MNRJ 20989; Serra do Amolar, RPPN Acurizal: UFMT 1422; Miranda: Guaycurus: MCP 592; Ponta Porã: IBSP 31459; Rio Pardo: MZUSP 11598; Santa Terezinha: Barra do

Tapirapé: MNRJ 401; Sidrolândia: MCN 6; Sonora: Ponte de Pedra Hydroelectric Power Plant: CEPB 7186; Terenos: IBSP 10011, USNM 100725; Três Lagoas: IBSP 14167, 21656, 21898, 22068; Urucum: FMNH 9198 (paratype). Minas Gerais: Araguari: IBSP 6915; Berilo: FUNED 2316; Boa Esperança: IBSP 21698; Campina Verde: Viva Cambota Farm: IBSP 70028; Conquista: Mendonça Power Plant, Sugar Cane Plantation: FUNED 378; Coromandel: FUNED 461; Formiga: Capão Grande Farm: FUNED 16; Formoso: Grande Sertão Veredas National Park: MZUSP 14470; Fortaleza de Minas: IBSP 84579; Fronteira: IBSP 52212, Furnas: IBSP 69234; Jacutinga: Gas pipeline Paulínia-Jacutinga: MZUSP 17625; Jaíba: Mocambinho: ZUFRJ 1108; João Pinheiro: Cantagalo Farm, Ruralminas I: MNRJ 15157, Esplanada, border of BR 040 Highway: MNRJ 15365; Veredas Farm, Rural Minas I: MNRJ 19999; Lassance: USNM 98784; Mateus Leme: IBSP 86711; Montes Claros: IBSP 32722, IBSP 32779; Nanuque, Serra dos Aimorés (MG); Paranaíba River: IBSP 46151; Patos de Minas: Cascata Farm: MNRJ 17294, Agricultural School: MNRJ 18805, 20264; Patrocínio: MZUSP 18988; Pirapora: MZUFV 325; Prata: IBSP 56102, 56204; Jaíba Project: FUNED 1464; Sacramento: IBSP 83540, 84538, 84580, 86703; Santa Fé de Minas: UFMG 1837; Santa Luzia: FUNED 2522; Santana do Riacho: IBSP 79052, 79053; Serra do Cipó: MCN 2943, Jaboticatubas: FUNED 1568, 1732; Três Marias: FUNED 2708; Uberaba: IBSP 68380; Uberlândia: Tenda dos Morenos Farm: MCP 2722; Irapé Hydroelectric Power Plant: MCN 4381, Cachoeira do Bananal Farm, left margin of Reservoir: MCN 1620 Cristália: Pc 7 Barreiro Farm, left margino of Reservoir: MCN 1833, Grão Mogol: MCN 2361; Unaí: Queimado Hydroelectric Power Plant: FUNED 1968, MCN 2208, 670; Veríssimo: IBSP 57145. Pará: Conceição do Araguaia: IBSP 17259, Santana do Araguaia: IBSP 49973, 49975. Paraná: Vila Alta: Parque Nacional de Ilha Grande, Ilha Bandeirantes: MHNCI 9303. Rondônia:

Chupinguaia: UFMT 11570; Forte Príncipe da Beira: MZUSP 3788. São Paulo: UMMZ 63009 (**paratype**), 63010 (**paratype**), 63011 (**paratype**), MCZ R-16700 (**holotype**); Aguaí: Chácara Primavera: IBSP 75469; Águas de São Pedro: IBSP 55812; Agudos: IBSP 52633; Altinópolis: IBSP 31835; Americana: IBSP 68865; Araçariguama: IBSP 62121, 62550, 67383; Araçatuba: IBSP 15177; Araras: Loteamento Jardins de Samantha IV: MZUSP 18778; Artur Nogueira: IBSP 10823; Barra Bonita: Iguatemi Train Station: IBSP 1615; Bebedouro: MCP 588, 590–91, 660; Boituva: IBSP 86349, MCP 16028; Boracéia: IBSP 50896; Botucatu: MNRJ 21661, Rio Bonito, Mina: MNRJ 20498, Vila Real da Barra: 24 Street: MNRJ 25955; Cabreuva: IBSP 84578; Cajamar: IBSP 86699; Cajuru: IBSP 48719; Campinas: IBSP 15434, Mata de Santa Genebra: Barão Geraldo: ZUEC 3667, Estiva Farm, Viracopos: ZUEC 47; Capivari: IBSP 62278, 85855; Casa Branca: ZUEC 2885; Catanduva: MCN 9978; Cristais Paulista: IBSP 55838; Descalvado: IBSP 28814, Rio do Pantano: MZUSP 1957; Dois Córregos: IBSP 32307; Echaporã: IBSP 30833; Elias Fausto: IBSP 70621; Emas: MZUSP 2669; Embaúba: IBSP 21623; Fernandópolis: IBSP 41828–29, 41836, 41888, 42065–66, 42069, 42072; Franca: MZUSP 1330–31, ZUEC 2887: Next to the Engenho Queimado Stream, ca. 100m from DINFRA: ZUEC 2871; Garça: IBSP 86025; Guarulhos: IBSP 84577; Hortolândia: ZUEC 2680; Ilha Solteira: 10–12 Km from Ilha Solteira Reservoir: IBSP 37122, 14–16 Km from Ilha Solteira Reservoir: IBSP 37118, 14/22 Km from Ilha Solteira Reservoir: IBSP 36039; Itapetininga: Campo Largo Farm: MZUSP 2690; Itapevi: IBSP 87951; Itapira: Paulínia-Jacutinga Gas Pipeline: MZUSP 17626, 17842; Itatinga: IBSP 55319; Itirapina: IBSP 75833, ZUEC 2794; Itu: IBSP 30563, 70937, 84007, 84595, 85866–67, 86725–26, 86915–16, 86942, 87146, MNRJ 12742, ZUEC 3772; Itupeva: IBSP 70101; Jaboticabal: IBSP 83949; Jaguariúna: ZUEC 48; Jundiaí: Serra do Japi: ZUEC 3737; Limeira: IBSP 62307; Lins: IBSP 45451; Louveira: IBSP

86024; Luiz Antônio: IBSP 82806–07; Maracai: IBSP 27248; Marília: MBML 292; Matão: IBSP 26659, Toriba Train Station: IBSP 10769; Mogi das Cruzes: IBSP 53824; Morro Agudo: IBSP 55355; Motuca: IBSP 11828; Nova Aliança: IBSP 53115–16; Olímpia: IBSP 50899; Oriente: IBSP 52352; Paraguaçu Paulista: IBSP 84178; Paulínia: MZUSP 13027; Perobal: MCP 587; Piracicaba: IBSP 86187, MZUSP 1329, Barra Bonita Reservoir: Anhumas: ZUEC 49; Pirassununga: IBSP 52217; Pitangueiras: Est? Plínio Prado: MCP 659; Porto Feliz: IBSP 11297; Presidente Epitácio: IBSP 12897, 60074, 60261; Ribeirão Preto: IBSP 7148; Rincão: Timbira Road: IBSP 15620; Rinópolis: IBSP 51443–44; Rio Claro: IBSP 30202, MHNCI 7480; Rio do Peixe: MZUSP 11599; Rubinéia: IBSP 38951; Sales de Oliveira: IBSP 30114, 55311; Salto: MCN 14802, 15366; Santa Adélia: IBSP 7903; Santa Clara do Oeste: IBSP 15974; Santa Cruz do Rio Pardo: IBSP 45450; Santa Ernestina: IBSP 10902; Santa Fé do Sul: IBSP 38544; Ponte Pensa: IBSP 38754; Santa Maria da Serra: IBSP 45452; Santana de Parnaíba: IBSP 51904; São Carlos: IBSP 30058, 52651; São João da Boa Vista: IBSP 55989; São João do Pau D'Alho: IBSP 53267; São Joaquim da Barra: IBSP 82497–98, Retiro Small Hydroelectric Central: MHNCI 15562; São José do Rio Preto: IBSP 75882, MZUSP 5477; São Paulo IBSP 86159, Vila das Mercês: IBSP 87952; São Pedro: IBSP 58263, 60160, 61216, 75193; São Simão: IBSP 75490; Sorocaba: IBSP 1536, Cajuru do Sul, political boundary with Itu: MHNCI 4489; Sumaré: IBSP 86663; Hortolândia: ZUEC 50; Tambaú: IBSP 15400; Taquaritinga: IBSP 8428; Tatuí: IBSP 83539; Teodoro Sampaio: Parque Estadual do Morro do Diabo: MZUSP 15298; Tietê: Sítio Pitangueiras: IBSP 66753; Turiba do Sul: IBSP 15371, IBSP 15372; Uberaba: IBSP 61121; Valinhos: IBSP 86653; Vargem Grande do Sul: Sítio Dois Irmãos: MHNCI 4073, 4075; Vinhedo: IBSP 45455; Vista Alegre do Alto: MCP 589; Votuporanga: MHNCI 4265. Tocantins: Colinas do Tocantins: CEPB 218; Guaraí:

MZUSP 12678; Macauba: Ilha do Bananal: MPEG 36; Palmas: IBSP 66337, 66453, 66458, MZUSP 12076, Luís Eduardo Magalhães Hydroelectric Power Plant: IBSP 66344, 66448; Peixe: IBSP 74235, 74443; Santa Terezinha: Ilha do Bananal: MZUSP 3149; Peixe Angical Hydroelectric Power Plant: MZUSP 15559. **PARAGUAY:** Amambay: Bella Vista: Estancia San Juan, 20 Km N de Bella Vista: CM 109101, Route III, 20 km N Route V: MNHNP 6636; Ascunción: Asunción: AMNH R-75500, Primavera: AMNH R-99160; Canindeyú: Colônia Indígena M'boi Jaguá: MNHNP 2985, Reserva Natural del Bosque Mbaracayú: MNHNP 3196; Concepción: Yby Jaú: MNHNP 2493, 95 km E from Concepción: MNHNP 5133, 30 km N Estancia San Fernando: MNHNP 5155, Parque Nacional Serranía San Luís: MNHNP 6635; San Pedro: III, 40 km N Río Aguaray Guazú: MNHNP 2494.

Boa constrictor constrictor. Unknown locality: NMR 10 (**syntype**), UUZM 79 (**syntype**), IBSP 8641. **BOLIVIA:** Unknown locality: IBSP 33289; Beni: Guayaramerin: next to Guayaramerin: AMNH R- 101915, Santa Rosa: Mamoré River: AMNH R- 101914, Siete Islas: Mamoré River: AMNH R- 101916; Santa Cruz: MVZ 9314, Sara: Buena Vista: CM R2937, UMMZ 149648, Surutu River, W of Buena Vista: CM R2843. **BRAZIL:** Unknown locality: IBSP 17428, MNRJ 22962. Acre: Cruzeiro do Sul: ZUEC 106, 176; Marechal Thaumaturgo: Boca do Tejo River; Alto Juruá: ZUEC 1588; Rio Branco: MZUSP 2490; MNRJ 8884, Senador Guimoard: Catuaba Experimental Farm: IBSP 87725; Porto Walter: 5 km N of Porto Walter, Juruá River: MPEG 19117. Alagoas: Olho d'Água Casado: ZUFRJ 609; Piranhas: Xingó Hydroelectric Power Plant: MZUFBA 886. Amapá: Almeriana: IBSP 25481; Laranjal do Jari: Iratapuru Village: Santo Antônio do Jari Hydroepectric Power Plant: INPA 32814; Macapá: Fazendinha: MNRJ 8875; Serra do Navio: ICOMI Village: MPEG

18349. Amazonas: AM-070 Manaus-Macapuru Highway: INPA (uncatalogued): low Mataurá River, Madeira Tributary: MZUSP 5914; Meduini Lake, left margin of Negro River: INPA 2186; Parque Nacional de Anavilhanas: INPA 10957; Prainha: Aripuanã River: MZUSP 5195; Reserva do Gavião: INPA 988; Amazonas River: IBSP 15219; Serrinha: Japurá River: MZUSP 6591; Tapajós: CZPB-RP 621; Madeira River: Left bank: INPA 13165; Balbina: INPA 19766; Beruri: MZUSP 5780; Km 350 from BR-319 Highway: INPA 32045; Careiro da Várzea: km 12 from Road to Autazes: MPEG 19542; Coari: Porto Urucu: MPEG 21156, CZPB-RP 623; Humaita: MCP 2963, MNRJ 19781, 21627, 23145; Itacoatiara: AM 010 Highway: Wilmadeiras Farm: INPA 34281; Igarapé Marajá: MPEG 23780; Urubu river: MNRJ 18941; Lábrea: Madeirera Scheffer: Ituxi River: MPEG 20400; Lindóia: INPA 31456; Manacapuru: Marapuru Road: MNRJ 21018; Manaus: Industrial zone: MHNCI 6407, road to the airport: INPA 36502, km 25 from BR 174 Highway: INPA 34096; Reman Petrobrás: INPA 17719; Tupé Sustainable Development Reserve: INPA 16063; Adolpho Ducke Reservation: INPA 20151, 21650; Universidade Federal do Amazonas: INPA 16073, IBSP 14359, 21091, 22926, 33024; Manicoré: Km 300 from BR-319 Highway: INPA 25722; Maués: Osório: Paraconi River: MPEG 23825; São Tomé: Paraconi River: MPEG 23834; Novo Airão: Jaú National Park: CZPB-RP 622, 624, IBSP 80555; Itacoatiara: MNRJ 17942. Bahia: Unknown municipality: MNRJ 2272; Andaraí: MZUESC 5785; Barra: MNRJ 2271; Bom Jesus da Lapa: MNRJ 2270, 2273–74; Feira de Santana: IBSP 79093; Igaporã: MZUESC 13086; Ituaçu: MZUESC 7538; Morro do Chapéu: Alagoas Farm: MZUESC 13775; Mucugê: MZUESC 5419; Itaparica: Paulo Afonso Hydroelectric Power Plant: MZUFBA 304–07. Ceará: Acaraú: CHIFCE 88, 221, 265; Fortaleza: Mucuripe: MNRJ 1991–92, 1966. Goiás: Ituaçu: Ordália: MCP 8946; BR-153 Highway: entrance to Pirenóplois, 14 km N of Interlândia: MHNCI 10144. Maranhão: Arari: Gancho do

Arari: BR-222 Highway, between Miranda do Norte and Arari: MPEG 13504, 25987; Bacabal: BR-316 Highway, 3 km from Bacabal, left margin of Mearim River: MPEG 17795; Barra do Corda: Aldeia Sapucaia (Guajajara): BR-226 Highway: MPEG 16123; Carolina Estreito Hydroelectric Power Plant: MPEG 24391, MZUSP 18746; Imperatriz: Suzano Papel e Celulose Industry: MHNCI 14124; Junco do Maranhão: Nova Vida: BR-316 Highway 25 km from the Gurupi River: MPEG 10227, 10232; Pindaré-Mirim: Puraqueú: BR-222 Highhway: MPEG 15699; Santa Luzia do Paruá: Paruá: BR-316 Highhway: MPEG 13614, 14225; São Luís: Porto de Itaqui: Suzano: MHNCI 15015, Tegram: MHNCI 15028. Mato Grosso: Alta Floresta: road to Juara: Apiacás Small Hydroelectric Central: UFMT 5490; Araputanga: Greenhouse, Ombreiras Small Hydroelectric Central: UFMT 6040; Aripuanã: UFMT 7222, MZUSP 11386, MZUFV 1443, 1512; Cláudia: Continental Farm: UFMT 3858; Cotriguaçu: Castanhal Road, São Nicolau Farm: UFMT 8598; Guiratinga: MZUSP 17481; Juína: Diagen Base: UFMT 4863; Nova Ubiratã: road between ARS Small Hydroelectric Central and Santo Antonio do Rio Bonito: UFMT 5360, 5372, 5441; Paranaíta: Balsa Nova border: UFMT 7767; Casa Verde: UFMT 7779; Casa Branca Road: UFMT 7773; Foz do Apiacás Hydroelectric Power Plant: UFMT 7114; Teles Pires Hydroelectric Power Plant: UFMT 7711, Tapajós River: MNRJ 6368; Sinop: IBSP 51959; Vila Bela da Santíssima Trindade: Madeira Electric Ramal Branch: MZUSP 19825; Vila Rica: MZUSP 11437. Pará: Unknown municipality: Xingu: Juruá River: MZUSP 9329: Jacaré Lake: Trombetas River: MZUSP 3825; PA-70 (current BR-222) Highway, next to Serra do Gradaús: MPEG 21264; Socego, Tributary of Tracuateua River: MZUSP 7664; Acará: Km 16 from Road to Acará: MPEG 10946, 15492; Almeirim: Pacanari Reservation: Jarí: MPEG 20104, Jari Florestal e Agropecuaria, 45 km W of Monte Dourado: MZUFV 690; Altamira: Belo Monte Hydroelectric Power Plant: MPEG 22398;

Ananindeua: CEPLAC area, BR-316 Highway: MPEG 17602, Seminário Pio X: MPEG 265; Arari: IBSP 14623; Aveiro: Uricurituba: MPEG 25019; Barcarena: MPEG 18441–42; Belém: MCP 1512, 16354, 2533, IBSP 79094–97, MCN 6048, 7691, MNRJ 8876, UFRGS 1590, ZUFRJ 1341; EMBRAPA: MPEG 16819; Ilha de Cotijuba: MPEG 18979; Ilha do Mosqueiro: MPEG 15521, Terra Firme: MPEG 25551; Benevides: Pratinha, Road to Genipauba: MPEG 7576, Pratinha Road to Genipauba, old road to Açucareiro: MPEG 13303; Bragança: Bom Jesus: MPEG 11411, 20251; Cachoeira do Arari: Teso dos Bichos: Ilha do Marajó: MPEG 18018; Canaã dos Carajás: MPEG 26020, 26463; Serra do Sossego Mining: FUNED 2837, MCN 1721; Canindé: Gurupi River: MZUSP 4250; Capitão Poço: Boca Nova: MPEG 1628; São Pedro: MPEG 9650–51; Conceição do Araguaia: IBSP 17252, 17262, 21987; Concórdia do Pará: Concórdia-Tomé Açú Road, km 12 from PA-15 Highway: MPEG 11721, 11725; Curuá: Road to Maú: MPEG 1881; Curuçá: Candeua: MPEG 21284; Marauá: MPEG 5879; Dom Eliseu: Sítio Bela Vista, BR-222 Highway: MPEG 14274; Engenho Santo Antonio: Furo do Panaquera: MZUSP 5118; Faro: Sítio Céu Estrelado, Nhamundá River, 15 km from Faro: MPEG 18352, 18355; Fordlândia: MZUSP 5127; Oriximiná: INPA 31468, MHNCI 16472, Floresta Nacional Saracá-Taquera: Igarapé do Moura: MZUSP 15277, Platô Saracá: MHNCI 15226, MNRJ 16818, Platô Bacaba: Porto Trombetas: MHNCI 13446, MPEG 22304–05, Road Porto Trombetas-Oriximiná: MNRJ 14919; Igarapé-Açu: MPEG 947; Itaituba: MCN 12624; Amazônia National Park: Tapajós River: MPEG 21221, 25049, 25051; Itupiranga: IBSP 33335; Juruti: Capiranga: MPEG 24428; Sapupara: INPA 35687, MPEG 22423; Marabá: MPEG 25341, 25827, 26021; Maracanã: Km 23 from Road to Maracanã: MPEG 4863; Marajó: Igarapé Taperebá: MZUSP 3653; Marituba: IBSP 68864; Melgaço: Floresta Nacional de Caxiuanã: Marinau: MPEG 21584, 21585; Moju: Santa Helena Farm: MPEG 25151;

Monte Alegre: Flexal: MCP 7624; Monte Cristo: Tapajós River: MZUSP 5149; Mosqueiro: IBSP 54146; Muaná: Ilha do Marajó: MPEG 15187; Obidos: MCZ R-1222; Ourilândia do Norte: MCN 5883, MPEG 24468, 25307; Palestina do Pará: Araguaia River: Posto Jarbas Passarinho: Transamazônica: MPEG 12934; Paragominas: IBSP 46157, MHNCI 3070; Peixe-Boi: MPEG 671, 1389; Poção: MZUSP 10791; Porto de Moz: Xingu Electric Ramal Branch: MZUSP 17759; Santa Bárbara do Pará: MPEG 21516, Parque Ecológico de GUNMA: MPEG 21327; Santa Cruz do Arari: Ilha do Marajó: MPEG 19634–35; Santana do Araguaia: IBSP 49108, 49974; Santarém: MCP 10624, 11189, 11456, 11805, 15449, 18140; Alter do Chão: MPEG 19037; Curua-Una Hydroepectric Power Plant: MCP 7920; Santo Antônio do Tauá: IBSP 76144, MPEG 6975, 7556; São Domingos do Capim: MPEG 8460; Taperinha: MZUSP 4807; Tomé-Açu: Vila Nova (Paraíso): Tomé Açú-Paragominas Road: MPEG 11746–47; Tucuruí: Tocantins River, right bank 2 Km S of Jacundá: MPEG 16727, 16755; Vai-Quem-Quer: MZUSP 10789; Vigia: Santa Rosa: road to Vigia: MPEG 6893, 6899; Viseu: Km 220 from BR-316 Highway: Contagem de Capanema: MPEG 8052, 3254. Paraíba: Cabedelo: Praia do Poço: MZUSP 7816; Campina Grande: IBSP 85057, São José da Mata: MNRJ 20335; Mamanguape: Sítio Caiama: ZUEC 1737; Souza: Acauã Farm: MNRJ 2007. Piauí: Unknown municipality: Uruçuí-Una Ecological Station: MZUSP 17279; Bom Jesus: IBSP 81301; Brejo do Piauí: UFMT 8604; Ilha Grande: CZDP (J2) 49; Parnaíba: CZDP (J2) 148; Piracuruca: Sete Cidades National Park: MPEG 22853; Piripiri: Road to Dom Pedro: MNRJ 11369; São Raimundo Nonato: MNRJ 7600–02, Serra da Capivara National Park: MNRJ 8343, 8397; Sebastião Leal: Faveira Farm: MHNCI 14379; Teresina: MBML 1223; Valença: MZUSP 5791. Rondônia: Alto Paraíso: Porto Velho-Araraquara Electric Ramal Branch: UFMT 10603; Espigão D'Oeste: Jaburi Farm: INPA 12150; Mutum Paraná: Jirau Hydroelectric Power Plant:

MZUSP 20582; Porto Velho: MZUSP 3141–42, UFRO 211–13, Madeira River, left bank: UFRO 2558–60, MZUSP 20806, 19082, right bank: INPA 32048, MZUSP 18575, 20842, Santo Antônio Hydroelectric Power Plant, right bank: INPA 27841, Jací-Paraná River: Santo Antônio Hydroelectric Power Plant: MPEG 23986, MZUSP, Vila Cachoeira do Samuel: MNRJ 3036. Roraima: Unknown municipality: BR-174 Highway, frontier landmark BV-8: MZUSP 9673, 9861; Cachoeira do Cujubim: Catrimani River: MZUSP 6399; Apiaú: MZUSP 9770; Boa Vista: MZUSP 9106; Caracaraí: Caicubi Village: Juvari River: MZUSP 19665; Catrimani Mission: MZUSP 10400, Catrimani: BR-210 Highway: 4 Km E of Ajarani River: MPEG 19006, BR-174 Highway: 44km W of Catrimani: MPEG 19416; Ilha de Maracá: MZUSP 10693; Santa Maria do Boiaçu: MZUSP 10326. Tocantins Babaçulândia: MZUSP 13013, Estreito Hydroelectric Power Plant: MPEG 24716; Lajeado: Lajeado Hydroelectric Power Plant: IBSP 64246, 64587; Palmas: Luís Eduardo Magalhães Hydroelectric Power Plant: MHNCI 10692, 10719; Palmeirante: MZUSP 18729. **COLOMBIA:** Bolivar: near Santa Rosa: MZUSP 6136; Caquetá: Morelia: ANSP 25655; Meta: Guacalito: CM R2022, Villavicencio: MZUSP 6051, 6053, MCZ R-183274; Putumayo: near Putumayo River: MVZ 33698. **ECUADOR:** Sucumbíos: Santa Cecilia: KU 148279–81, 152749, 7 km E Agrio Lake on the road to Coca: KU 158787. **FRENCH GUIANA:** 4.8 km SW Maripasoula-Lumber Road: MCZ R-77588. **GUYANA:** Unknown locality: AMNH R-61079, BYU 42811; Barima-Waini: Mabaruma: USNM 164195, cemetery: USNM 164196; Cuyuni-Mazaruni: Kartabu: CM S4101; Essequibo Islands-West Demerara: Dunoon Demerara River: UMMZ 53975, 53977. Upper-Demerara-Berbice: Dubulay Ranch, next to the ranch house: USNM 497803, USNM 16482; Upper-Takutu-Upper-Essequibo: Aishalton: ca. 24 km NE: USNM 566251. **PERU:** Amazonas: Najem River [Tributary of Huampami River, Cenepa River Drainage]: MVZ 163374; Caterpiza:

Caterpiza River tributary of Santiago River: USNM 566532–33; La Poza: Santiago River: USNM 566536; Puerto Galilea: Santiago River: USNM 566534–35; Loreto: high bank of Ucayali River: AMNH R- 71131; Samiria River: Parinari Cañon: AMNH R-57259; Iquitos: AMNH R- 56118, TCWC 42059–60; Itaya River: AMNH R- 54537; Pampa Hermosa: Cushabatay River: AMNH R- 55958; Pebas: ANSP 11474–75; Requena: Monte Carmelo: AMNH R- 53484. Madre De Dios: Puerto Maldonado: ca. 30 km SSW of Tambopata Reserve, Explorer's Inn: USNM 222347. Ucayali: 60 Km SW Pucallpa: ANSP 34345; Coengua River, High Ucayali River: AMNH R- 53125. **SURINAME:** Unknown locality: ANSP 6716–17; Commewijne: Plantation Ma Retraite, in the other side of Paramaribo River: AMNH R- 130484; Paramaribo: Paramaribo: AMNH R- 130481, IBSP 18456, 2 Rijweg (Kwatta): CM 44286, Weg naar Zee: AMNH R- 130483. **TRINIDAD & TOBAGO:** Tobago: Stant John: ca. 1 km N of Speyside on Windward Road: USNM 228014, ca. 1 km SW of Speyside, on Windward Road: USNM 228013; Trinidad: AMNH R- 3038, 98165, MCZ R-6105, USNM 17759–60, 50686; Arima: Sant George: USNM 166678; Diego Martin Regional Corporation: Maqueripe: AMNH R- 64531; Tunapuna/Piarco: Sant George, Mount Saint Benedict: CM S6531, S6541; San Rafael: FMNH 49927. **VENEZUELA:** Amazonas: San Carlos: Negro River: 3 km NE San Carlos: AMNH R- 127825, Tamatama: Orinoco River: USNM 217145; Apure: San Fernando de Apure: 6.0 km W: TCWC 47880; Aragua: Camatague: Route 2: 10 Km S Camatague: TCWC 47882; Bolivar: Arabopó: UMMZ 85270; Capital District: Caracas: ANSP 6709, 6718; Fálcon: El Mamon: MCZ R-133907, Urumaco: MCZ R-133955; Guárico: Altagracia De Orituco: 13 km N of Altagracia de Orituco: TCWC 58702; Neblina Base camp in Mawarinuma River: AMNH R- 129247; Miranda: Quiripital: Parque Guatopo: TCWC 58731; Santa Lucia: 11 Km E on Quebrada Siquire: CM S7453; Nueva Esparta: Boca del Rio: Parguaza

River, ca. 25 km of Boca del Rio: UCM 65259; Sucre: Cumanacoa: CM S7891, El Yaque: next to Turumiquiri: CM S7968, Pantanillo: road to Cumanacoa: KU 117030, San Antonio del Golfo: KU 117031; Zulia: Maracaibo: AMNH R-94291.

Boa constrictor melanogaster. **ECUADOR**: Morona-Santiago: Yaupi Mission: Yaupi River: Upper Santiago River: KU 192081–83 (**paratypes**); UMMZ 172676–79 (**paratypes**), 172680 (**holotype**), 172681 (**paratype**).

Boa nebulosa. **DOMINICA:** unknown locality: UMMZ 203783, 145440. Sant Andrew Parish: Caye en Bouc: KU 260004; Marigot: USNM 161004; Woodford Hill: MCZ R-65493 (**holotype**), 65494 (**paratype**), 176903. Sant David Parish: Concord: KU 260001. Sant George Parish: Bellevue Chopin: KU 260005, Governor: USNM 161009, Near Roseau: MVZ 211940–41, Trafalgar Falls: MCZ R-58772 (**paratype**), 74371 (**paratype**). Sant John Parish: Conner: USNM 161005, Moore Park State: MCZ R-65492 (**paratype**), Portsmouth: MCZ R-6106 (**paratype**). Sant Joseph Parish: Brantridge: CAS 113542, Clarke Hall: USNM 153847–50, 156875, 160620, Clarke Hall: Layou River Valley: USNM 160226, Layou Park: MCZ R-65495 (**paratype**). Sant Patrick Parish: Norway: KU 260006. Sant Paul Parish: 11 km NE Roseau: KU 260007, 9.6 km NE Roseau: KU 260008, Springfield: USNM 161007, USNM 161008.

Boa occidentalis. Unknown locality: IBSP 10139, 72731, 78246, 78325, 78352, 83333, MCN 10046. **ARGENTINA**: unknown locality: MCP 17643, 18303. Chaco: Almirante Brown: Concepción del Bermejo: MACN 34425, El Zapallar: UMMZ 94098, between Três Isletas and Castelli: FML 1905, Nueve de Julio: Las Breñas: MACN 35449, Pampa

del Infierno: MACN 47709, 48670. Córdoba: Deán Funes: MACN 48669, Lucio V. Mansilla: MACN 48671, Quinta Jaime Roca, near los Reartes: MACN 47710, Río Seco: Paraje "La estancia", W of Villa de Maria: MACN 40026, Valle de La Punilla: MACN 48676. Corrientes: Ituzaingó: MACN 7612. Formosa: Bermejo: near Guadalcazar: MACN 39749, near Lamadrid: MACN 39744, Pozo de Maza: MACN 9452. Patiño: Ruta Provincial Nº 25, 15 Km of Subteniente Perín, way to Colonia Alto Alegre: MACN 39850. La Rioja: MACN 39720, 48675. General Belgrano: Ruta Nacional 38. near Córdoba: FML 24503, Vera Peñaloza. Ruta Nacional 79, km 105 and 33km S of Desiderio Tello FML 9470. Salta: Anta: 10 km S of General Pizarro: FML 2210, Finca Pozo Largo. 12 km E Finca. San Javier: FML 7360, Ruta Nacional 16. 17 km E of Tolloche: FML 17929, Ruta 41, 20 km E of Joaquim V. González: FML 2196. Chaguaral: MACN 48667, General José de San Martín: Tartagal: MACN 23731, General Guemes: Ruta Nacional 9. km 1529. Past Cruz Quemada before Guemes: FML 14303, Orán: between Saucelito and Santa Rosa: MACN 34421. San Luis: Belgrano: 40 km NW San Luis from 147 Route: MVZ 128174, near Villa General Roca: MACN 40025, Junín: Quebrada de Cautana: MACN 45425. Santa Fé: Nueve de Júlio: Tostado: MACN 8444. Santiago del Estero: Aguirre: Malbrán: MACN 34347, Banda: Huyamampa: FML 40, Costa del Salado: FML 63, El Mojón: FML 6527, Gramilla: MACN 48674. Loreto: 30 km S of Loreto: FML 15889, R. N. 9. at the entrance of Villa Atamisqui: FML 23193. Pampa de los Guanacos: MACN 47708, Pellegrini: FML 2278–79, 2290, near Nueva Esperanza: FML 2261-1–3, 2262-2–4, R. N. 176. 80 km S of Nueva Esperanza: FML 28405. Tucumán: Burruyacú: Finca la Virginia: FML 17629, La Zanja: FML 2227, Punta del Água: FML 1700, Ruta Nacional 34. between Las Cejas and General Piedrabuena: FML 6410, La Capital: Campo norte: FML 9614, San Miguel de Tucumán: FML 225, Jujuy 400: FML 18372, Trancas: Obispo Piedrabuena: FML

14016. **BOLIVIA:** Santa Cruz: San Antonio de Parapetí: AMNHR- 141664. **PARAGUAY:** unknown locality: MNHNP 3979, 9434. Alto Paraguay: Mayor Pablo Lagerenza: Parque Nacional Defensores de Chaco: MNHNP 7241. Boquerón: 14 km S of Filadelfia: MNHNP 5135, Filadelfia: 20 km east of Filadelfia: MNHNP 246, Parque Nacional Teniente Enciso: MNHNP 3789. Filadelfia: 48 km S of Filadelfia: MNHNP 5134, Presidente Hayes: Estancia Pozo Azul: MNHNP 2986, Parque Nacional Tinfunqué: MNHNP 4045, Reserva Indígena ″Casanillo″: MNHNP 5132.

Boa orophias. **SANTA LUCIA:** unknown locality: NMR uncatalogued (**holotype**), MCZ R-6711, USNM 418. Anse-La-Raye: "Adjodha": MCZ R-75842–43, 75847. Venus Estate: KU 260009–13. Praslin: Praslin: MCZ R-75848. **SANTA KITTS & NEVIS**: Santa Kitts: MCZ R-6659.
